# The Exploration of TPhos as a Monodentate P‑Ligand for Palladium-Catalyzed Regioselective Hydrothiocarbonylation of Styrenes under Neutral Conditions

**DOI:** 10.1021/acs.orglett.6c02424

**Published:** 2026-06-29

**Authors:** Chang-Sheng Kuai, Wen Che, Shou-Fei Zhu, Xiao-Feng Wu

**Affiliations:** † Dalian National Laboratory for Clean Energy, 58279 Dalian Institute of Chemical Physics, Chinese Academy of Sciences, Dalian 116023 China; ‡ Academy for Advanced Interdisciplinary Studies, Frontiers Science Center of New Organic Matters, State Key Laboratory and Institute of Elemento Organic Chemistry, College of Chemistry, 12538 Nankai University, Tianjin 300071, China; § 28392 Leibniz-Institut für Katalyse e. V., Albert-Einstein-Straβe 29a, 18059 Rostock, Germany

## Abstract

A palladium-catalyzed nonacidic hydrothiocarbonylation of styrenes with CO and thiols has been developed. Utilizing a cyclopropane-based monophosphine ligand (TPhos), this protocol enables the efficient and branched-selective synthesis of alkyl thioesters under mild and neutral conditions with broad substrate scope and excellent functional group tolerance.

## Graphical abstract

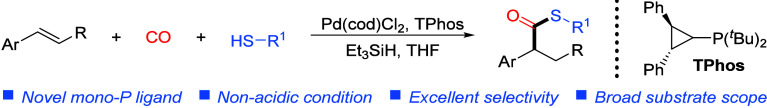

Thioesters are privileged motifs widely embedded in various bioactive molecules,^1^ pharmaceuticals^2^ (e.g., Dalcetrapib, Stepronin, Formicin A), and functional materials.^3^ Owing to the inferior orbital overlap of the carbon–sulfur bond compared to that of conventional esters, these motifs exhibit distinctive thermodynamic reactivity, positioning them as indispensable acyl donors in both biochemical pathways and organic synthesis.^4^ Consequently, thioesters serve as versatile and adaptable synthetic handles, enabling divergent functional group transformations to efficiently access ketones, amides, and alcohols via cross-coupling or nucleophilic acyl substitution reactions.^5^ Consequently, the development of efficient strategies for thioester synthesis remains highly desirable. Traditional methods mainly rely on the condensation^6^ or oxidative acylation of thiols with carboxylic acids derivatives, aldehydes, as well as transition-metal-catalyzed carbonylative coupling of thiols with aryl halides.^7^ Although widely used, these approaches often require stoichiometric activators or oxidants, harsh conditions, and generate significant waste. Therefore, these drawbacks have stimulated considerable interest in developing more efficient, atom-economical, and selective methods.

In particular, transition-metal-catalyzed hydrothiocarbonylation of olefins with carbon monoxide and thiols provides a straightforward and regioselectively tunable route to thioesters from readily available feedstocks. However, compared to well-established carbonylations such as hydroesterification, hydroamidation, and hydroformylation,^8^ the development of hydrothiocarbonylation has lagged significantly behind, primarily due to the severe poisoning effects of sulfur on metal catalysts and the relatively slow development of suitable ligands. Early pioneering studies,^9^ mainly from Alper and co-workers, were primarily limited to activated olefins, including allenes, vinylcyclopropanes, allylic alcohols, and conjugated dienes. Driven by recent advent of advanced ligand design, significant progress has been achieved in controlling the regioselectivity of unactivated or less activated alkenes. For instance, Fleischer and co-workers reported a palladium-catalyzed branched-selective hydrothiocarbonylation of styrene derivatives using a bisphosphine ligand under in situ generated CO (Scheme 1A).^10^ Subsequently, the Liao group developed an asymmetric version employing chiral sulfoxide-(P-dialkyl)­phosphine (SOP) ligands.^11^ Our group also achieved linear-selective hydrothiocarbonylation of olefins using bisphosphine ligands.^12^

**Scheme 1:**
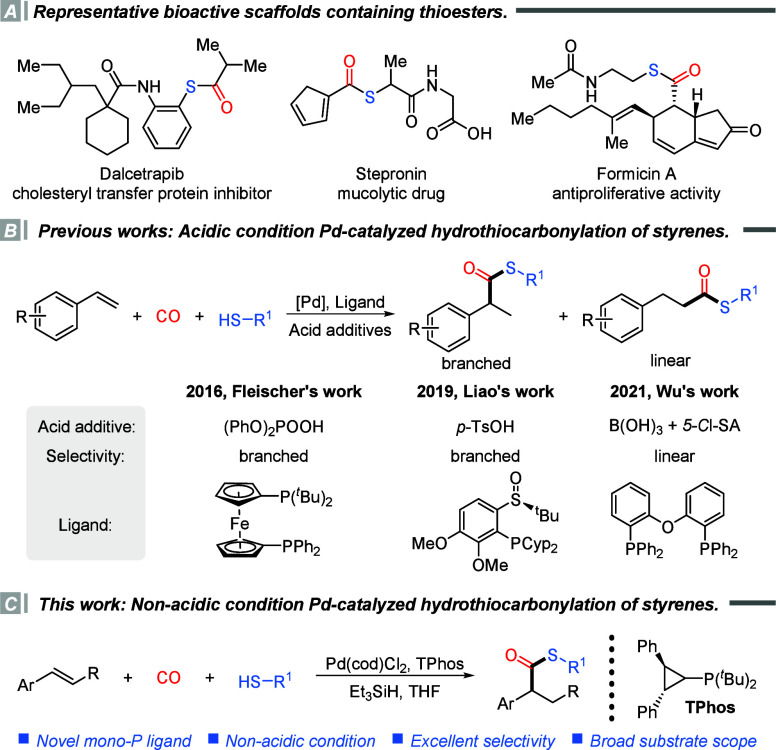
Importance of Thioesters and Hydrothiocarbonylation Strategies for Their Synthesis

Despite these elegant achievements, however, the aforementioned catalytic systems are all based on acidic conditions, typically employing acidic additives to facilitate the generation of the active Pd–H species. Consequently, the indispensable requirement for acid significantly compromises substrate scope and catalytic efficiency. For example, basic substrates or functional groups (strongly electron-deficient olefins exhibit markedly reduced reactivity) often lead to poor yields due to unwanted protonation, and challenging internal olefins exhibit minimal reactivity under these regimes. Therefore, the development of nonacidic and highly efficient catalytic systems that function under mild, neutral conditions is highly desirable to overcome these limitations and fully unlock the synthetic potential of hydrothiocarbonylation.

Building on our continued interest in carbonylation chemistry, and also inspired by the newly developed monophosphine ligands by Zhu and co-workers,^13^ we herein report a palladium-catalyzed nonacidic hydrothiocarbonylation of styrenes with CO and thiols. Utilizing a novel TPhos ligand, this protocol enables the efficient and branched-selective synthesis of aliphatic thioesters under mild and neutral conditions. Notably, the avoidance of acidic additives significantly broadens the substrate scope, allowing the successful transformation of strongly electron-deficient olefins and challenging internal alkenes, which are typically problematic in previously reported acidic systems.

At the outset, we initiated our investigation using styrene (**1a**) and 4-methylbenzenethiol (**2a**) as model substrates. The reaction was conducted under 10 bar of CO at 30 °C, employing Pd­(cod)­Cl_2_ as the catalyst precursor, triethylsilane a sacrificial hydride source to initiate the Pd–H cycle, and tetrahydrofuran (THF) as the solvent. We first examined the influence of ligands on the reaction outcome (Table 1, entries 1–5). While commonly used ligands such as PPh_3_ and PCy_3_ failed to deliver any desired product, the electron-rich ligand BuPAd_2_ afforded the branched aliphatic thioester in 47% yield with high regioselectivity. Remarkably, our newly designed cyclopropane mono-P ligand, TPhos, which features rotatable aryl substituents that dynamically modulate the catalytic cavity to adapt to the shifting steric demands of oxidative addition and reductive elimination, smoothly furnished the target product in near-quantitative yield (99%). Encouraged by this optimal ligand profile, we next examined the role and loading of the silane additive (Table 1, entries 6–8). In the absolute absence of Et_3_SiH, the transformation was completely suppressed, thereby substantiating our hypothesis that the silane plays a pivotal role in generating the active Pd–H catalyst species. Altering the loading of Et_3_SiH from the standard protocol, either by increasing or decreasing the amount, failed to improve the efficiency. Subsequently, solvent screening revealed that THF was uniquely effective, whereas highly polar solvents profoundly inhibited the reaction (Table 1, entries 9–15). Finally, the effect of palladium precursors was examined (Table 1, entries 16–19). Interestingly, nonhalogenated palladium complexes showed no catalytic activity, implying that the halide counteranion also plays an indispensable role in stabilizing the active catalytic intermediates or facilitating the turning over of the cycle.

**Table 1:**
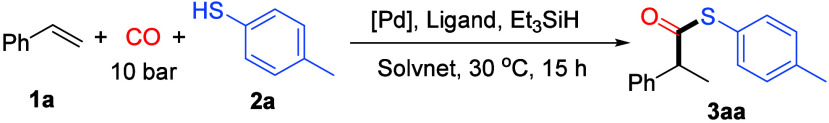
Optimization of Reaction Conditions^a^

Entry	Catalyst	Ligand	Solvent	Yield **3aa** (%)^b^
1	Pd(cod)Cl_2_	PPh_3_	THF	ND
2	Pd(cod)Cl_2_	PCy_3_	THF	ND
3	Pd(cod)Cl_2_	BuPAd_2_	THF	47
4	Pd(cod)Cl_2_	TPhos	THF	99
5	Pd(cod)Cl_2_	TPhos-Ph	THF	ND
6	Pd(cod)Cl_2_	TPhos	THF	ND^c^
7	Pd(cod)Cl_2_	TPhos	THF	80^d^
8	Pd(cod)Cl_2_	TPhos	THF	98^e^
9	Pd(cod)Cl_2_	TPhos	dioxane	93
10	Pd(cod)Cl_2_	TPhos	toluene	82
11	Pd(cod)Cl_2_	TPhos	MBTE	ND
12	Pd(cod)Cl_2_	TPhos	DCE	ND
13	Pd(cod)Cl_2_	TPhos	DMF	ND
14	Pd(cod)Cl_2_	TPhos	DMSO	ND
15	Pd(cod)Cl_2_	TPhos	CH_3_CN	ND
16	PdBr_2_	TPhos	THF	82
17	Pd(OAc)_2_	TPhos	THF	ND
18	Pd_2_(dba)_3_	TPhos	THF	ND
19	Pd/C	TPhos	THF	ND

a Reaction conditions: **1a** (0.2 mmol), **2a** (0.24 mmol), [Pd] (5 mol %), Ligand (10 mol %), Et_3_SiH (40 mol %), CO (10 bar), Solvent (1.0 mL), 30 °C, 15 h.

b Yield was determined by GC using ^*n*^Hexadecane as the internal standard.

c Without Et_3_SiH.

d Et_3_SiH (30 mol %).

e Et_3_SiH (50 mol %). MBTE: methyl-*tert*-butylether. BuPAd_2_: di­(1-adamantyl)-*n*-butylphosphine. TPhos-Ph: (2,3-diphenylcyclopropyl)­diphenylphosphane.

To evaluate the generality of the novel TPhos ligand in palladium-catalyzed hydrothiocarbonylation of styrenes, we next examined the substrate scope with respect to thiols under the optimized conditions (Scheme 2). A wide range of thiophenols bearing electronically diverse para-substituents were first investigated. Substrates possessing electron-donating groups (such as *tert*-butyl and methoxy), halogens (fluorine, bromine), and a strongly electron-withdrawing trifluoromethyl moiety were all well-tolerated, smoothly delivering the corresponding aliphatic thioesters in good to excellent yields (**3aa**–**3af**). Notably, the bromo-substituted thiophenol proceeded smoothly to afford the desired product **3ae** in 81% yield, providing a valuable handle for late-stage functionalization. Additionally, naphthalene-2-thiol was compatible, furnishing the target product in a good yield. We then evaluated the steric impact of *ortho*-monosubstituted thiophenols, all of which successfully delivered the target products in excellent yields (**3ah**–**3ak**). However, further investigation into highly sterically hindered di-*ortho*-substituted thiophenols revealed complete inhibition of the transformation, presumably due to severe steric hindrance that impedes the nucleophilic attack step­(**3al**). Furthermore, various *meta*-substituted thiophenols were examined and found to react efficiently, consistently affording the desired thioesters in good yields (**3am**–**3ao**). Finally, we investigated aliphatic thiols. Primary, secondary, and tertiary thiols were all found to be competent substrates in this catalytic system. Notably, tertiary thiols exhibited superior reactivity, affording the corresponding thioesters in excellent yields (**3ap**–**3ar**). It is worth mentioning that phenol, aniline, and butylamine were also tested under our standard conditions, but no desired product was detected.

**Scheme 2:**
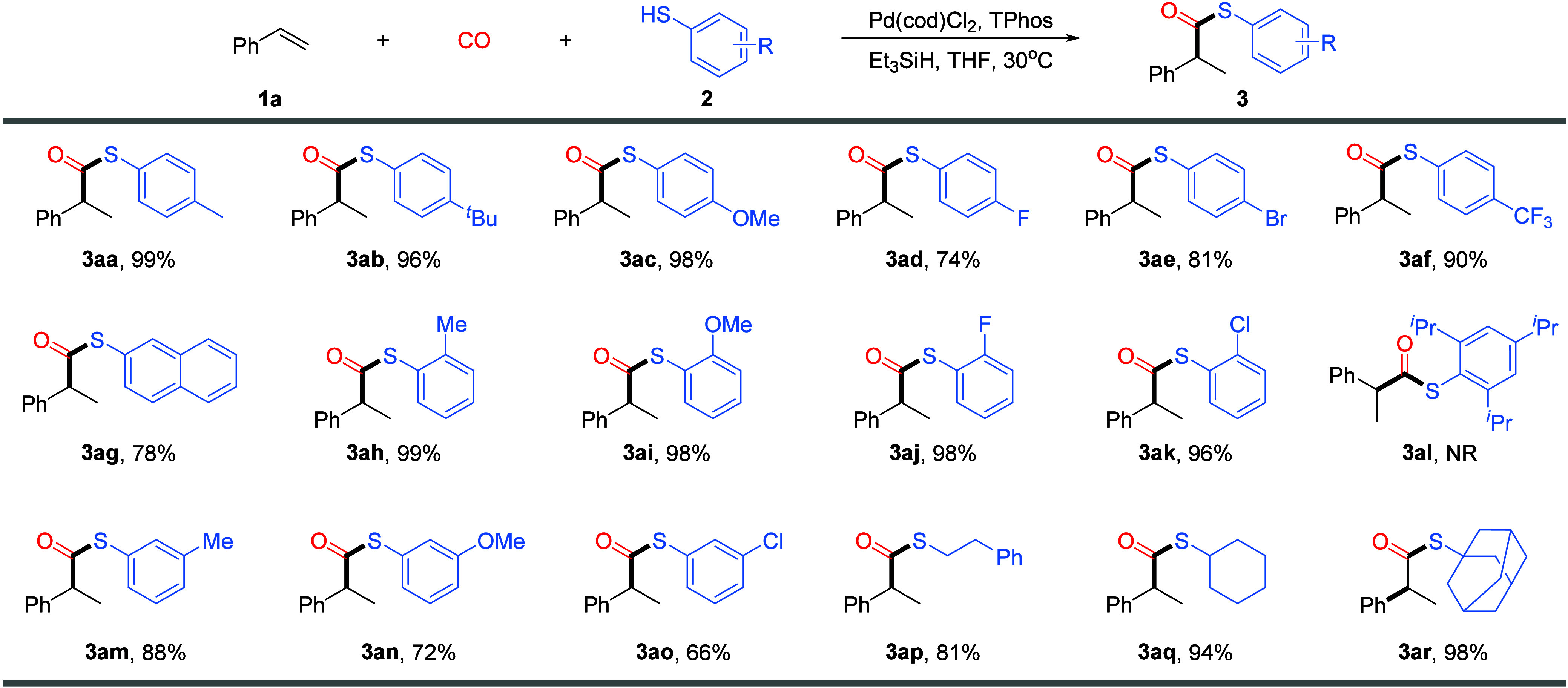
Scope of Thiophenols and Thiols^a^ ^,^ ^b^

a Reaction conditions: **1a** (0.2 mmol), **2a** (0.24 mmol), Pd­(cod)­Cl_2_ (5 mol %), TPhos (10 mol %), Et_3_SiH (40 mol %), CO (10 bar), THF (1.0 mL), 30 °C, 15 h.
b Isolated yields.

Having established the scope of thiols, we further evaluated the generality of this protocol with respect to the styrenes component (Scheme 3). Styrene derivatives possessing electron-donating groups at the *para*-position, such as *tert*-butyl and methoxy groups, smoothly engaged in the transformation to deliver the corresponding thioesters in excellent yields (**3ba**, **3ca**). *Para*-halogenated styrenes (F, Cl, and Br) were also competent coupling partners, furnishing the desired products in moderate to good yields (**3da**–**3fa**). Remarkably, styrenes equipped with strongly electron-withdrawing groups, including trifluoromethyl, ester, and even the nitro group — which is often incompatible with palladium hydride catalysis — all proceeded efficiently to afford the target architectures in excellent yields (**3ga**–**3ia**). Furthermore, the pinacol boronate ester (-Bpin) functionality, which offers potential for further derivatization, was compatible with the catalytic system, affording the thioester **3ja** in 72% yield. Subsequently, the spatial and electronic effects of *ortho*- and *meta*-substituted styrenes were scrutinized. Variations including *ortho*-methyl, chloro, and bromo groups, as well as *meta*-chloro and bromo substituents, were fully compatible with this catalytic platform (**3ka**–**3oa**). Although a slight decrease in efficiency was observed for the bromo-substituted substrates, the retention of the C–Br bond provides an invaluable molecular handle for late-stage diversification. Other classes of aromatic alkenes, such as 2-vinylnaphthalene and 2-vinylthiophene, were similarly accommodated, cleanly delivering the expected alkyl thioesters (**3pa**, **3qa**). Intriguingly, when challenging asymmetric internal alkenes were subjected to the optimized conditions, the reaction rate expectedly slowed down, yet the excellent branched regioselectivity was rigorously maintained, with no other regioisomers detected (**3ra**). Finally, we tested the reactivity of electron-rich, unactivated aliphatic alkenes; however, no desired products were detected. This limitation is presumably due to the absence of aryl conjugation, which may be crucial for facilitating migratory insertion or stabilizing key intermediates in the catalytic cycle.

**Scheme 3:**
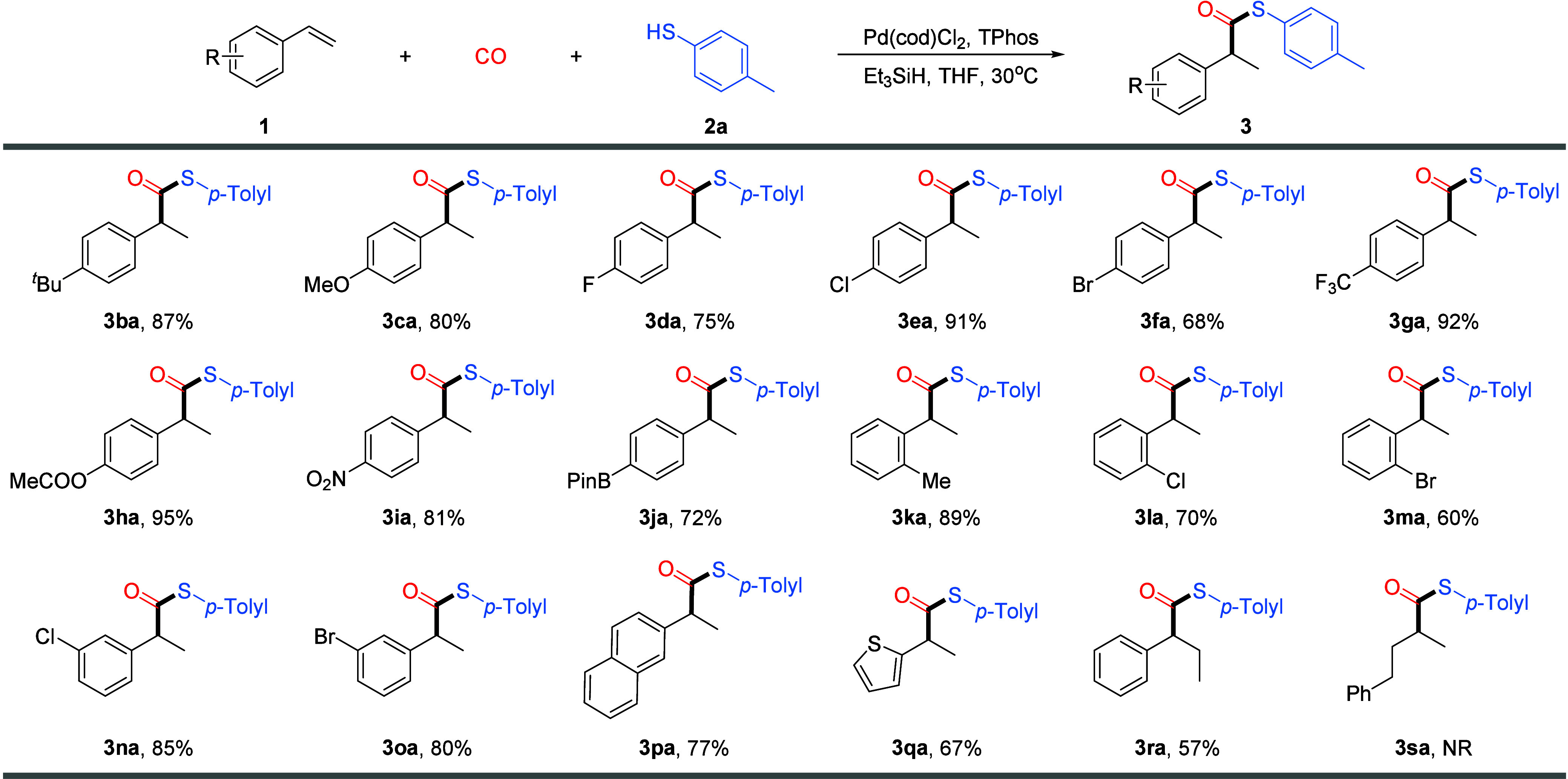
Scope of Styrenes^a^ ^,^ ^b^

a Reaction conditions: **1a** (0.2 mmol), **2a** (0.24 mmol), Pd­(cod)­Cl_2_ (5 mol %), TPhos (10 mol %), Et_3_SiH (40 mol %), CO (10 bar), THF (1.0 mL), 30 °C, 15 h.
b Isolated yields.

To demonstrate the practical utility and scalability of this newly developed methodology, a scaled-up reaction was performed (Scheme 4A). When the reaction was escalated to a 2.0 mmol scale, the catalytic efficiency remained robust without any noticeable loss in reactivity, smoothly furnishing the target thioester in an excellent 95% yield. This successful amplification underscores the high reproducibility and significant potential of this protocol for large-scale synthetic applications.

**Scheme 4:**
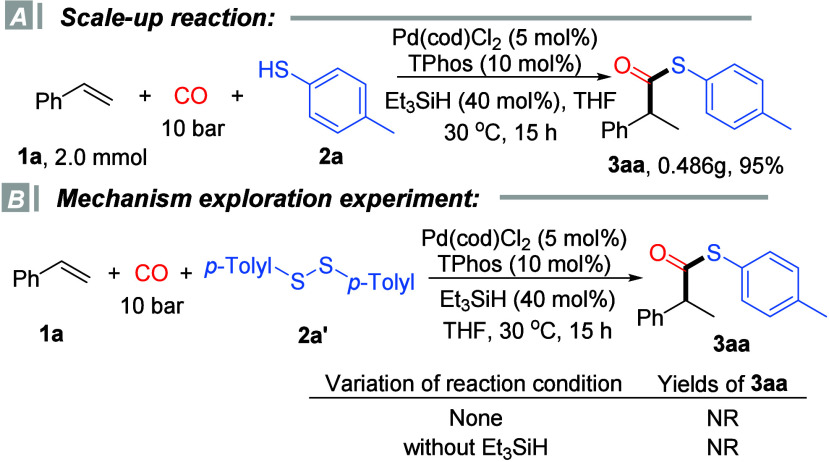
Scale-Up Reaction and Mechanistic Studies

Subsequently, we turned our attention to elucidating the underlying mechanism of this novel TPhos/palladium-catalyzed hydrothiocarbonylation. During our initial condition optimization, we occasionally observed small quantities of diaryl disulfide as a byproduct, presumably arising from the oxidative dimerization of thiols. This observation prompted us to hypothesize whether the disulfide could act as a key reactive intermediate involved in the catalytic cycle. To test this possibility, control experiments were designed using *p*-tolyl disulfide as the alternative sulfur source instead of the 4-methylbenzenethiol (Scheme 4B). However, no desired thioester product was detected, regardless of the presence or absence of the triethylsilane additive. These definitive outcomes clearly rule out the disulfide-mediated pathway, validating that the disulfide is merely an inactive byproduct rather than a competent intermediate in this hydrothiocarbonylation process.

Based on previous literature precedents on related carbonylative reactions and our experimental observations,^14^ a plausible catalytic cycle for the present palladium-catalyzed hydrothiocarbonylation is proposed in Scheme 5. As illustrated, the active L–Pd-H species is initially generated in situ with the assistance of the TPhos ligand, triethylsilane, and the thiol. This palladium hydride complex then coordinates with styrene (**1**) to form intermediate complex which subsequent regioselective migratory insertion of the styrene into the Pd–H bond affords the branched benzylpalladium intermediate **B**. Insertion of carbon monoxide into the Pd–C bond of **B** then generates the key acylpalladium species **C**. Finally, nucleophilic attack of the thiol (**2a**) on the acylpalladium intermediate **C** delivers the desired branched thioester product **3** and simultaneously regenerates Pd^0^ after reductive elimination which then reacts with PhSH via oxidative addition to give the active L–Pd-H species.

**Scheme 5:**
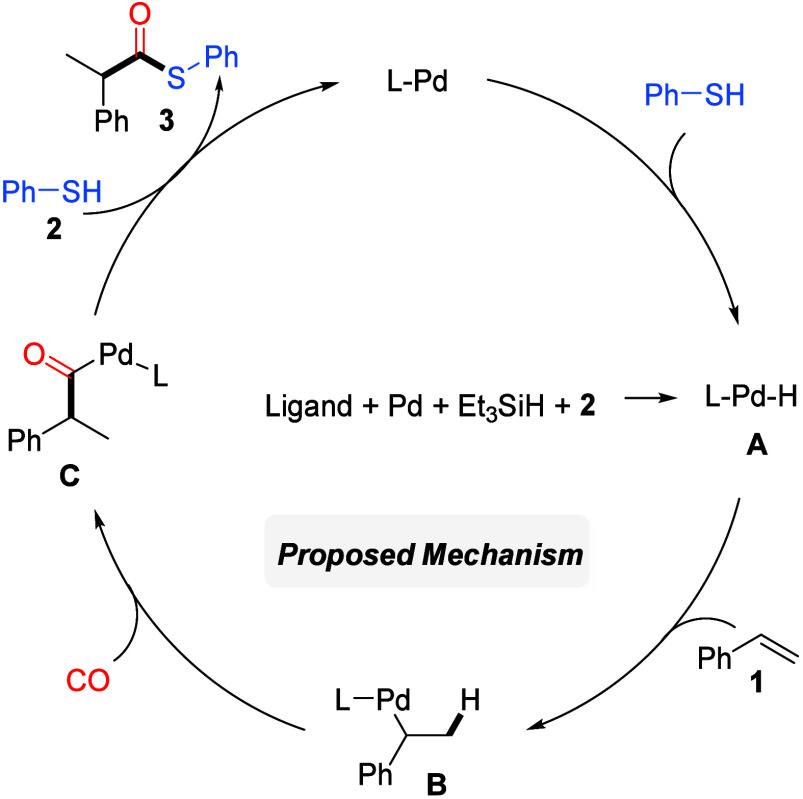
Proposed Mechanism

In summary, we have developed a novel palladium-catalyzed hydrothiocarbonylation of styrenes with carbon monoxide and thiols using a newly designed cyclopropane monophosphine ligand (TPhos). Operating under mild, nonacidic, and neutral conditions, this method enables the efficient and branched-selective synthesis of aliphatic thioesters with a significantly broadened substrate scope. The protocol exhibits excellent functional group tolerance toward both styrenes and thiols. This work advances the field of carbonylative chemistry and provides a practical and general approach to valuable thioesters for applications in organic synthesis, medicinal chemistry, and materials science. Further exploration of the unique properties of TPhos in other carbonylation reactions is currently underway in our laboratory.

## Supplementary Material



## Data Availability

The data underlying this study are available in the published article and its Supporting Information.

## Notes

Experimental details, characterization data and NMR spectra (PDF)
