# Supplementary material for: The Exploration of TPhos as a Monodentate P‑Ligand for Palladium-Catalyzed Regioselective Hydrothiocarbonylation of Styrenes under Neutral Conditions
Source: Org Lett. 2026 Jun 29;28(27):8718–23. doi: 10.1021/acs.orglett.6c02424 (PMC13366686; doi:10.1021/acs.orglett.6c02424)

Supporting Information

The Exploration of TPhos as a Monodentate P-Ligand for Palladium-Catalyzed Regioselective Hydrothiocarbonylation of Styrenes under Neutral Conditions

Chang-Sheng Kuai,[a] Wen Che,[b] Shou-Fei Zhu,[b]* and Xiao-Feng Wu[a,c]*

[a] Dalian National Laboratory for Clean Energy, Dalian Institute of Chemical Physics, Chinese Academy of Sciences, Dalian 116023 China, E-mail: xwu2020@dicp.ac.cn

[b] Academy for Advanced Interdisciplinary Studies, Frontiers Science Center of New Organic Matters, State Key Laboratory and Institute of Elemento Organic Chemistry, College of Chemistry, Nankai University, Tianjin 300071, China, E-mail: sfzhu@nankai.edu.cn

[c] Leibniz-Institut für Katalyse e. V., Albert-Einstein-Straβe 29a, 18059 Rostock, Germany

Table of Contents

1. General experimental details. 2

2. Typical procedure for Pd-catalyzed hydrothiocarbonylation of styrenes. 3

3. Scale up reaction. 3

4. Mechanistic studies. 3

5. Spectroscopic Data of Products. 5

6. NMR Spectra of the Products. 14

1. General experimental details.

Unless otherwise noted, all reactions were carried out under a carbon monoxide or nitrogen atmosphere. The reagents were ordered from Adamas-beta®, Energy Chemical Sigma-Aldrich, Bidepharm and used without purification. All solvents were dried by standard techniques and distilled prior to use. Column chromatography was performed on silica gel (200-300 meshes). All NMR spectra were recorded at ambient temperature using Bruker Avance III 400 MHz NMR (^{1}H, 400 MHz; ^{13}C {^{1}H}, 101 MHz, ^{19}F 376 MHz), Bruker AVANCE III HD 700MHz NMR spectrometers (^{1}H, 700 MHz; ^{13}C{^{1}H}, 100 MHz). ^{1}H NMR chemical shifts are reported relative to TMS and were referenced via residual proton resonances of the corresponding deuterated solvent ($CDCl_3$: 7.26 ppm) whereas ^{13}C{^{1}H} NMR spectra are reported relative to TMS via the carbon signals of the deuterated solvent ($CDCl_3$: 77.0 ppm). Data for ^{1}H are reported as follows: chemical shift (δ ppm), multiplicity (s = singlet, d = doublet, t = triplet, q = quartet, quint = quintet, m = multiplet, br = broad), coupling constant (Hz), and integration. All ^{13}C NMR spectra were broad-band ^{1}H decoupled. All reactions were monitored by GC-FID or NMR analysis. HRMS data was obtained with Micromass HPLC-Q-TOF mass spectrometer (ESI) or Agilent 6540 Accurate-MS spectrometer (Q-TOF).

Because of the high toxicity of carbon monoxide, all the reactions should be performed in an autoclave. The laboratory should be well-equipped with a CO detector and alarm system.

2. Typical procedure for Pd-catalyzed hydrothiocarbonylation of styrenes.

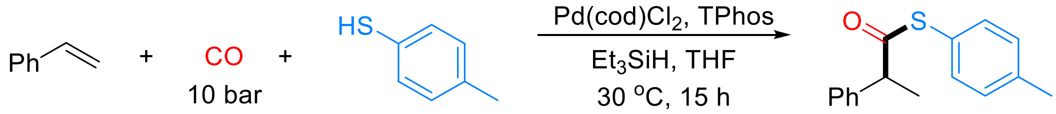

1a **2a** **3aa**

A 4 mL screw-cap vial was charged with Pd(cod)Cl_2 (5 mol%), Tphos (10 mol%), Et_3SiH (40 mol%), styrene (**1a**, 0.20 mmol), 4-methylbenzenethiol (**2a**, 0.24 mmol), THF (1.0 mL) and an oven-dried stir bar. The vial was closed with a Teflon septum and cap and connected to the atmosphere via a needle. The vial was moved to an alloy plate and put into a Parr 4560 series autoclave (300 mL) under an argon atmosphere. At room temperature, the autoclave was flushed with CO three times and charged with 10 bar of CO. The autoclave was placed on a heating plate equipped with a magnetic stirrer and an aluminum block. The reaction mixture was heated to 30 ^{o}C for 15h. After the reaction was complete, the autoclave was cooled down with ice water to room temperature and the pressure was released carefully. The reaction mixture concentration under reduced pressure, the crude product was purified by column chromatography on silica gel to afford the corresponding product **3aa**.

3. Scale up reaction.

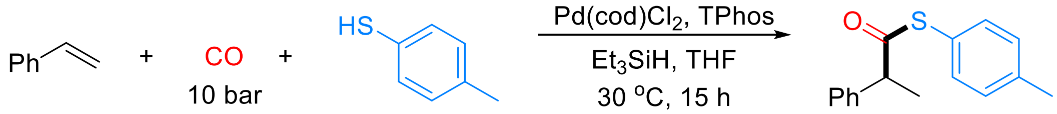

1a **2a** **3aa**

A 20 mL screw-cap vial was charged with Pd(cod)Cl_2 (5 mol%), Tphos (10 mol%), Et_3SiH (40 mol%), styrene (**1a**, 2.0 mmol), 4-methylbenzenethiol (**2a**, 2.4 mmol), THF (5.0 mL) and an oven-dried stir bar. The vial was closed with a Teflon septum and cap and connected to the atmosphere via a needle. The vial was moved to an alloy plate and put into a Parr 4560 series autoclave (300 mL) under an argon atmosphere. At room temperature, the autoclave was flushed with CO three times and charged with 10 bar of CO. The autoclave was placed on a heating plate equipped with a magnetic stirrer and an aluminum block. The reaction mixture was heated to 30 ^{o}C for 15h. After the reaction was complete, the autoclave was cooled down with ice water to room temperature and the pressure was released carefully. The reaction mixture was concentrated under reduced pressure, and the crude product was purified by column chromatography on silica gel to afford the corresponding product **3aa** (486.4 mg, 95% yield).

4. Mechanistic studies.

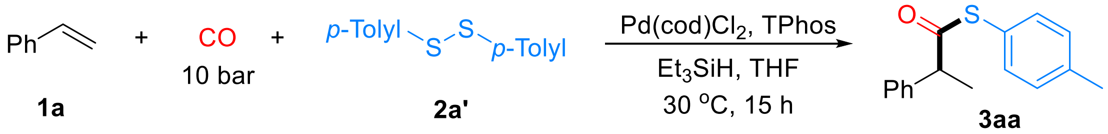

Variation of reaction condition	Yields of **3aa**
None	NR
without Et_3SiH	NR

A 4 mL screw-cap vial was charged with $Pd(cod)Cl_2$ (5 mol%), Tphos (10 mol%), Et_3SiH (40 mol%, with or without), styrene (**1a**, 0.20 mmol), *p*-tolyl disulfide (**2a'**, 0.24 mmol), THF (1.0 mL) and an oven-dried stir bar. The vial was closed with a Teflon septum and cap and connected to the atmosphere via a needle. The vial was moved to an alloy plate and put into a Parr 4560 series autoclave (300 mL) under an argon atmosphere. At room temperature, the autoclave was flushed with CO three times and charged with 10 bar of CO. The autoclave was placed on a heating plate equipped with a magnetic stirrer and an aluminum block. The reaction mixture was heated to 30 ^{o}C for 15h. After the reaction was complete, the autoclave was cooled down with ice water to room temperature and the pressure was released carefully. The result was determined by GC using ^{n}Hexadecane as the internal standard.

5. Spectroscopic Data of Products.

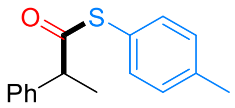

S-(*p*-tolyl)-2-phenylpropanethioate (3aa):

Colorless oil, 50.7 mg, 99 % yield, R_f=0.2 (PE/EtOAc 40/1).

^{1}H NMR (400 MHz, $CDCl_3$) δ 7.35 (d, J = 3.9 Hz, 4H), 7.32 – 7.26 (m, 1H), 7.22 (d, J = 7.5 Hz, 2H), 7.17 (d, J = 7.9 Hz, 2H), 3.99 (q, J = 7.0 Hz, 1H), 2.34 (s, 3H), 1.56 (d, J = 7.1 Hz, 3H).

^{13}C NMR (100 MHz, $CDCl_3$) δ 199.5, 139.7, 139.6, 134.5, 130.0, 128.8, 128.1, 127.6, 124.4, 54.0, 21.4, 18.7.

HRMS (ESI-TOF) m/z: $[M + H]^+$ calculated for $C_{16}H_{17}OS$ 256.0995; Found 256.0999.

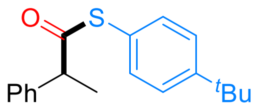

S-(4-(*tert*-butyl)phenyl)-2-phenylpropanethioate (3ab):

Colorless oil, 57.2 mg, 96% yield, R_f=0.2 (PE/EtOAc 40/1).

^{1}H NMR (400 MHz, $CDCl_3$) δ 7.38 (d, J = 8.4 Hz, 2H), 7.35 (d, J = 4.2 Hz, 4H), 7.31 – 7.25 (m, 3H), 3.99 (q, J = 7.1 Hz, 1H), 1.56 (d, J = 7.1 Hz, 3H), 1.30 (s, 9H).

^{13}C NMR (100 MHz, $CDCl_3$) δ 199.6, 152.6, 139.7, 134.2, 128.8, 128.1, 127.6, 126.3, 124.5, 54.0, 34.8, 31.3, 18.8.

HRMS (ESI-TOF) m/z: $[M + H]^+$ calculated for $C_{19}H_{23}OS$ 299.1464; Found 299.1466.

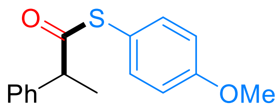

S-(4-methoxyphenyl)-2-phenylpropanethioate (3ac):

Brown oil, 53.2 mg, 98% yield, R_f=0.2 (PE/EtOAc 20/1).

^{1}H NMR (400 MHz, $CDCl_3$) δ 7.34 (d, J = 4.3 Hz, 4H), 7.31 – 7.27 (m, 1H), 7.24 (d, J = 8.7 Hz, 2H), 6.88 (d, J = 8.7 Hz, 2H), 3.98 (q, J = 7.0 Hz, 1H), 3.78 (s, 3H), 1.56 (d, J = 7.1 Hz, 3H).

^{13}C NMR (100 MHz, $CDCl_3$) δ 200.0, 160.6, 139.7, 136.1, 128.8, 128.1, 127.6, 118.6, 114.8, 55.4, 53.8, 18.7.

HRMS (ESI-TOF) m/z: $[M + H]^+$ calculated for $C_{16}H_{17}O_2S$ 273.0944; Found 273.0944.

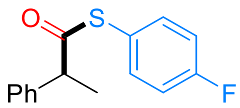

S-(4-fluorophenyl)-2-phenylpropanethioate (3ad):

Colorless oil, 38.4 mg, 74% yield, R_f=0.2 (PE/EtOAc 40/1).

^{1}H NMR (400 MHz, $CDCl_3$) δ 7.34 (d, J = 4.7 Hz, 4H), 7.32 – 7.25 (m, 3H), 7.05 (t, J = 8.6 Hz, 2H), 3.98 (q, J = 7.0 Hz, 1H), 1.57 (d, J = 7.1 Hz, 3H).

^{13}C NMR (100 MHz, $CDCl_3$) δ 199.1, 163.4 (d, J = 249.9 Hz), 139.4, 136.6 (d, J = 8.5 Hz), 128.9, 128.1, 127.7, 123.2 (d, J = 3.4 Hz), 116.4 (d, J = 22.1 Hz), 54.1, 18.6.

^{19}F NMR (376 MHz, $CDCl_3$) δ -111.29.

HRMS (ESI-TOF) m/z: $[M + H]^+$ calculated for $C_{15}H_{14}FOS$ 261.0744; Found 261.0747.

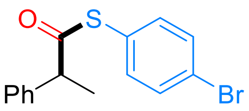

S-(4-bromophenyl)-2-phenylpropanethioate (3ae):

Colorless oil, 51.6 mg, 81% yield, R_f=0.2 (PE/EtOAc 40/1).

^{1}H NMR (400 MHz, $CDCl_3$) δ 7.48 (d, J = 8.5 Hz, 2H), 7.39 – 7.27 (m, 5H), 7.19 (d, J = 8.5 Hz, 2H), 3.98 (q, J = 7.1 Hz, 1H), 1.57 (d, J = 7.1 Hz, 3H).

^{13}C NMR (100 MHz, $CDCl_3$) δ 198.4, 139.3, 135.9, 132.3, 128.9, 128.1, 127.8, 127.0, 124.0, 54.2, 18.6.

HRMS (ESI-TOF) m/z: $[M + H]^+$ calculated for $C_{15}H_{14}B_rOS$ 320.9943; Found 320.9943.

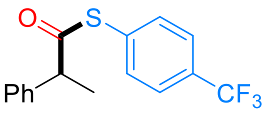

S-(4-(trifluoromethyl)phenyl)-2-phenylpropanethioate (3af):

Light yellow oil, 55.9 mg, 90% yield, R_f=0.2 (PE/EtOAc 40/1).

^{1}H NMR (400 MHz, $CDCl_3$) δ 7.53 (d, *J* = 8.1 Hz, 2H), 7.39 (d, *J* = 8.0 Hz, 2H), 7.33 – 7.20 (m, 5H), 3.92 (q, *J* = 7.0 Hz, 1H), 1.51 (d, *J* = 7.0 Hz, 3H).

^{13}C NMR (100 MHz, $CDCl_3$) δ 197.9, 139.1, 134.5, 132.7, 131.2 (q, J = 32.8 Hz), 128.9, 128.1, 127.9, 125.9 (q, J = 3.7 Hz), 123.8 (q, J = 272.5 Hz), 54.5, 18.5.

^{19}F NMR (376 MHz, $CDCl_3$) δ -62.85.

HRMS (ESI-TOF) m/z: $[M + H]^+$ calculated for $C_{16}H_{14}F_3OS$ 311.0712; Found 311.0716.

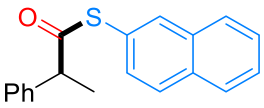

S-(naphthalen-2-yl)-2-phenylpropanethioate (3ag):

Light yellow oil, 45.3 mg, 78% yield, R_f=0.2 (PE/EtOAc 40/1).

^{1}H NMR (400 MHz, $CDCl_3$) δ 7.88 (s, 1H), 7.81 (d, J = 8.5 Hz, 2H), 7.79 – 7.75 (m, 1H), 7.52 – 7.44 (m, 2H), 7.41 – 7.34 (m, 5H), 7.34 – 7.27 (m, 1H), 4.04 (q, J = 7.1 Hz, 1H), 1.60 (d, J = 7.1 Hz, 3H).

^{13}C NMR (100 MHz, $CDCl_3$) δ 199.3, 139.6, 134.3, 133.5, 133.3, 130.9, 128.9, 128.7, 128.1, 128.0, 127.8, 127.7, 127.1, 126.5, 125.3, 54.2, 18.7.

HRMS (ESI-TOF) m/z: $[M + H]^+$ calculated for $C_{19}H_{17}OS$ 293.0995; Found 293.0998.

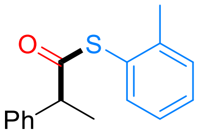

S-(*o*-tolyl)-2-phenylpropanethioate (3ah):

Light yellow oil, 50.8 mg, 99% yield, R_f=0.2 (PE/EtOAc 40/1).

^{1}H NMR (400 MHz, $CDCl_3$) δ 7.33 (dd, J = 10.5, 6.0 Hz, 5H), 7.30 – 7.22 (m, 3H), 7.16 (t, J = 7.3 Hz, 1H), 4.02 (q, J = 7.1 Hz, 1H), 2.19 (s, 3H), 1.56 (d, J = 7.1 Hz, 3H).

^{13}C NMR (100 MHz, $CDCl_3$) δ 198.6, 142.1, 139.7, 136.0, 130.7, 130.1, 128.8, 128.0, 127.6, 127.4, 126.6, 54.1, 20.6, 18.6.

HRMS (ESI-TOF) m/z: $[M + H]^+$ calculated for $C_{16}H_{17}OS$ 257.0995; Found 257.0995.

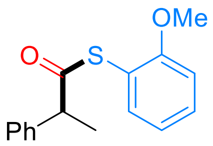

S-(2-methoxyphenyl) 2-phenylpropanethioate (3ai):

Yellow oil, 53.4 mg, 98% yield, R_f=0.2 (PE/EtOAc 20/1).

^{1}H NMR (400 MHz, $CDCl_3$) δ 7.39 – 7.25 (m, 7H), 6.97 – 6.88 (m, 2H), 4.02 (q, J = 7.1 Hz, 1H), 3.74 (s, 3H), 1.56 (d, J = 7.1 Hz, 3H).

^{13}C NMR (100 MHz, $CDCl_3$) δ 198.3, 159.2, 139.9, 136.6, 131.5, 128.7, 128.1, 127.5, 121.0, 116.3, 111.5, 55.9, 53.9, 18.8.

HRMS (ESI-TOF) m/z: $[M + H]^+$ calculated for $C_{16}H_{17}O_2S$ 273.0944; Found 273.0946.

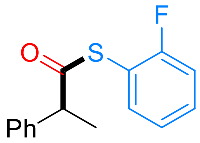

S-(2-fluorophenyl) -2-phenylpropanethioate (3aj):

Light yellow oil, 51.0 mg, 98% yield, R_f=0.2 (PE/EtOAc 40/1).

^{1}H NMR (400 MHz, $CDCl_3$) δ 7.41 – 7.29 (m, 7H), 7.12 (t, J = 8.0 Hz, 2H), 4.02 (q, J = 7.0 Hz, 1H), 1.58 (d, J = 7.1 Hz, 3H).

^{13}C NMR (100 MHz, $CDCl_3$) δ 197.3, 162.1 (d, J = 249.6 Hz), 139.3, 136.6, 132.0 (d, J = 8.1 Hz), 128.9, 128.1, 127.8, 124.6 (d, J = 3.8 Hz), 116.2 (d, J = 22.7 Hz), 115.5 (d, J = 18.6 Hz), 54.1, 18.7.

^{19}F NMR (376 MHz, $CDCl_3$) δ -106.47.

HRMS (ESI-TOF) m/z: $[M + H]^+$ calculated for $C_{15}H_{14}FOS$ 261.0744; Found 261.0747.

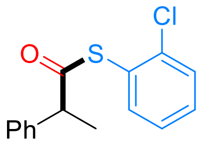

S-(2-chlorophenyl)-2-phenylpropanethioate (3ak):

Light yellow oil, 53.1 mg, 96% yield, R_f=0.2 (PE/EtOAc 40/1).

^{1}H NMR (400 MHz, $CDCl_3$) δ 7.46 (d, J = 8.0 Hz, 1H), 7.42 (d, J = 7.6 Hz, 1H), 7.36 (d, J = 4.1 Hz, 4H), 7.33 – 7.27 (m, 2H), 7.23 (t, J = 7.5 Hz, 1H), 4.02 (q, J = 7.1 Hz, 1H), 1.59 (d, J = 7.1 Hz, 3H).

^{13}C NMR (100 MHz, $CDCl_3$) δ 197.2, 139.3, 138.6, 137.1, 131.1, 130.2, 128.8, 128.1, 127.7, 127.5, 127.2, 54.2, 18.6.

HRMS (ESI-TOF) m/z: $[M + H]^+$ calculated for $C_{15}H_{14}ClOS$ 277.0448; Found 277.0450.

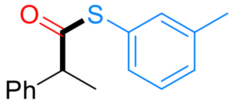

S-(*m*-tolyl)-2-phenylpropanethioate (3am):

Light yellow oil, 45.3 mg, 88% yield, R_f=0.2 (PE/EtOAc 40/1).

^{1}H NMR (400 MHz, $CDCl_3$) δ 7.35 (d, J = 4.3 Hz, 4H), 7.32 – 7.27 (m, 1H), 7.24 (d, J = 7.9 Hz, 1H), 7.16 (t, J = 7.5 Hz, 3H), 3.99 (q, J = 7.1 Hz, 1H), 2.32 (s, 3H), 1.57 (d, J = 7.1 Hz, 3H).

^{13}C NMR (100 MHz, $CDCl_3$) δ 199.3, 139.7, 139.0, 135.0, 131.5, 130.2, 129.0, 128.8, 128.1, 127.6, 127.5, 54.1, 21.3, 18.7.

HRMS (ESI-TOF) m/z: $[M + H]^+$ calculated for $C_{16}H_{17}OS$ 257.0995; Found 257.0996.

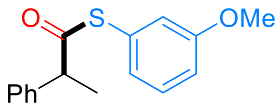

S-(3-methoxyphenyl)-2-phenylpropanethioate (3an):

Colorless oil, 39.4 mg, 72% yield, R_f=0.2 (PE/EtOAc 40/1).

^{1}H NMR (400 MHz, $CDCl_3$) δ 7.35 (d, J = 4.3 Hz, 4H), 7.32 – 7.22 (m, 2H), 6.96 – 6.88 (m, 3H), 3.99 (q, J = 7.1 Hz, 1H), 3.76 (s, 3H), 1.57 (d, J = 7.1 Hz, 3H).

^{13}C NMR (100 MHz, $CDCl_3$) δ 198.9, 159.8, 139.6, 129.9, 128.9, 128.8, 128.1, 127.6, 126.7, 119.5, 115.5, 55.4, 54.1, 18.7.

HRMS (ESI-TOF) m/z: $[M + H]^+$ calculated for $C_{16}H_{17}O_2S$ 273.0944; Found 273.0949.

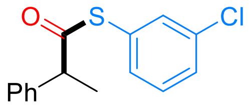

S-(3-chlorophenyl) -2-phenylpropanethioate (3ao):

Colorless oil, 36.5 mg, 66% yield, R_f=0.2 (PE/EtOAc 40/1).

^{1}H NMR (400 MHz, $CDCl_3$) δ 7.42 – 7.31 (m, 7H), 7.28 (d, J = 7.7 Hz, 1H), 7.22 (d, J = 7.5 Hz, 1H), 3.99 (q, J = 7.1 Hz, 1H), 1.58 (d, J = 7.1 Hz, 3H).

^{13}C NMR (100 MHz, $CDCl_3$) δ 198.2, 139.2, 134.6, 134.1, 132.6, 130.1, 129.7, 129.5, 128.9, 128.1, 127.8, 54.3, 18.6.

HRMS (ESI-TOF) m/z: $[M + H]^+$ calculated for $C_{15}H_{14}ClOS$ 277.0448; Found 277.0451.

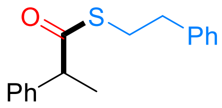

S-phenethyl-2-phenylpropanethioate (3ap):

Colorless oil, 43.5 mg, 81% yield, R_f=0.2 (PE/EtOAc 50/1).

^{1}H NMR (400 MHz, $CDCl_3$) δ 7.37 – 7.22 (m, 7H), 7.20 (d, J = 7.0 Hz, 1H), 7.15 (d, J = 7.3 Hz, 2H), 3.88 (q, J = 7.1 Hz, 1H), 3.06 (t, J = 7.8 Hz, 2H), 2.80 (t, J = 7.8 Hz, 2H), 1.52 (d, J = 7.1 Hz, 3H).

^{13}C NMR (100 MHz, $CDCl_3$) δ 201.0, 140.0, 139.9, 128.7, 128.6, 128.5, 127.9, 127.5, 126.5, 54.3, 35.8, 30.5, 18.4.

HRMS (ESI-TOF) m/z: $[M + H]^+$ calculated for $C_{17}H_{19}OS$ 271.1151; Found 271.1155.

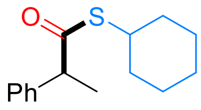

S-cyclohexyl-2-phenylpropanethioate (3aq):

Yellow oil, 46.5 mg, 94% yield, R_f=0.2 (PE/EtOAc 3/1).

^{1}H NMR (400 MHz, $CDCl_3$) δ 7.31 (d, J = 6.5 Hz, 4H), 7.28 – 7.23 (m, 1H), 3.84 (q, J = 7.1 Hz, 1H), 3.45 (td, J = 9.8, 4.8 Hz, 1H), 1.93 – 1.86 (m, 1H), 1.85 – 1.78 (m, 1H), 1.70 – 1.61 (m, 2H), 1.51 (d, J = 7.1 Hz, 3H), 1.45 – 1.31 (m, 4H), 1.31 – 1.13 (m, 2H).

^{13}C NMR (100 MHz, $CDCl_3$) δ 201.0, 140.1, 128.6, 127.9, 127.3, 54.3, 42.5, 33.1, 32.9, 26.0, 26.0, 25.6, 18.5.

HRMS (ESI-TOF) m/z: $[M + H]^+$ calculated for $C_{15}H_{21}OS$ 249.1308; Found 249.1312.

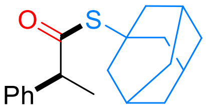

S-(adamantan-1-yl)-2-phenylpropanethioate (3ar):

Colorless oil, 58.6 mg, 98% yield, R_f=0.2 (PE/EtOAc 40/1).

^{1}H NMR (400 MHz, $CDCl_3$) δ 7.27 – 7.15 (m, 5H), 3.70 (q, J = 7.1 Hz, 1H), 2.01 (s, 6H), 1.93 (s, 3H), 1.67 – 1.58 (m, 6H), 1.39 (d, J = 7.1 Hz, 3H).

^{13}C NMR (100 MHz, $CDCl_3$) δ 201.5, 140.4, 128.6, 127.8, 127.2, 54.7, 50.9, 41.8, 36.3, 29.8, 18.6.

HRMS (ESI-TOF) m/z: $[M + H]^+$ calculated for $C_{19}H_{25}OS$ 301.1621; Found 301.1625.

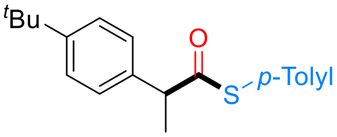

S-(*p*-tolyl)-2-(4-(tert-butyl)phenyl)propanethioate (3ba):

Colorless oil, 54.3 mg, 87% yield, R_f=0.2 (PE/EtOAc 40/1).

^{1}H NMR (400 MHz, $CDCl_3$) δ 7.36 (d, J = 8.3 Hz, 2H), 7.27 (d, J = 8.3 Hz, 2H), 7.23 (d, J = 8.1 Hz, 2H), 7.16 (d, J = 8.0 Hz, 2H), 3.96 (q, J = 7.1 Hz, 1H), 2.33 (s, 3H), 1.55 (d, J = 7.1 Hz, 3H), 1.32 (s, 9H).

^{13}C NMR (100 MHz, $CDCl_3$) δ 199.6, 150.4, 139.5, 136.5, 134.4, 129.9, 127.7, 125.7, 124.6, 53.6, 34.5, 31.4, 21.4, 18.8.

HRMS (ESI-TOF) m/z: $[M + H]^+$ calculated for $C_{20}H_{25}OS$ 313.1621; Found 313.1625.

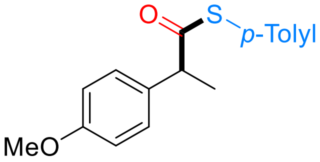

S-(*p*-tolyl)-2-(4-methoxyphenyl)propanethioate (3ca):

Yellow oil, 45.7 mg, 80% yield, R_f=0.2 (PE/EtOAc 20/1).

^{1}H NMR (400 MHz, $CDCl_3$) δ 7.26 (d, J = 8.6 Hz, 2H), 7.22 (d, J = 8.3 Hz, 2H), 7.16 (d, J = 8.1 Hz, 2H), 6.88 (d, J = 8.6 Hz, 2H), 3.93 (q, J = 7.1 Hz, 1H), 3.79 (s, 3H), 2.34 (s, 3H), 1.53 (d, J = 7.1 Hz, 3H).

^{13}C NMR (100 MHz, $CDCl_3$) δ 199.8, 159.1, 139.5, 134.5, 131.7, 129.9, 129.2, 124.6, 114.2, 55.3, 53.2, 21.3, 18.7.

HRMS (ESI-TOF) m/z: $[M + H]^+$ calculated for $C_{17}H_{19}O_2S$ 287.1100; Found 287.1102.

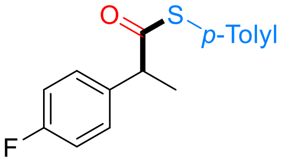

S-(*p*-tolyl)-2-(4-fluorophenyl)propanethioate (3da):

Colorless oil, 41.3 mg, 75% yield, R_f=0.2 (PE/EtOAc 40/1).

^{1}H NMR (400 MHz, $CDCl_3$) δ 7.31 (dd, J = 8.3, 5.5 Hz, 2H), 7.20 (q, J = 8.1 Hz, 4H), 7.03 (t, J = 8.6 Hz, 2H), 3.97 (q, J = 7.1 Hz, 1H), 2.34 (s, 3H), 1.55 (d, J = 7.1 Hz, 3H).

^{13}C NMR (100 MHz, $CDCl_3$) δ 199.4, 162.3 (d, J = 246.1 Hz), 139.7, 135.4 (d, J = 3.2 Hz), 134.4, 130.0, 129.6 (d, J = 8.1 Hz), 124.2, 115.6 (d, J = 21.4 Hz), 53.1, 21.3, 18.8.

^{19}F NMR (376 MHz, $CDCl_3$) δ -114.93.

HRMS (ESI-TOF) m/z: $[M + H]^+$ calculated for $C_{16}H_{16}FOS$ 275.0900; Found 275.0907.

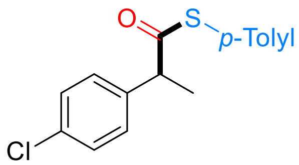

S-(*p*-tolyl)-2-(4-chlorophenyl)propanethioate (3ea):

Colorless oil, 52.6 mg, 91% yield, R_f=0.2 (PE/EtOAc 40/1).

^{1}H NMR (400 MHz, $CDCl_3$) δ 7.31 (d, J = 8.6 Hz, 2H), 7.27 (d, J = 8.6 Hz, 2H), 7.21 (d, J = 8.2 Hz, 2H), 7.17 (d, J = 8.1 Hz, 2H), 3.95 (q, J = 7.1 Hz, 1H), 2.34 (s, 3H), 1.54 (d, J = 7.1 Hz, 3H).

^{13}C NMR (100 MHz, $CDCl_3$) δ 199.1, 139.7, 138.1, 134.4, 133.5, 130.0, 129.4, 128.9, 124.1, 53.3, 21.4, 18.7.

HRMS (ESI-TOF) m/z: $[M + H]^+$ calculated for $C_{16}H_{16}ClOS$ 291.0605; Found 291.0607.

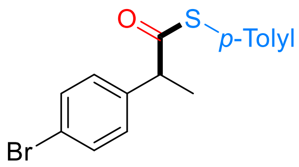

S-(*p*-tolyl)-2-(4-bromophenyl)propanethioate (3fa):

Yellow oil, 45.2 mg, 68% yield, R_f=0.2 (PE/EtOAc 40/1).

^{1}H NMR (400 MHz, $CDCl_3$) δ 7.47 (d, J = 8.4 Hz, 2H), 7.24 – 7.16 (m, 6H), 3.94 (q, J = 7.1 Hz, 1H), 2.35 (s, 3H), 1.54 (d, J = 7.1 Hz, 3H).

^{13}C NMR (100 MHz, $CDCl_3$) δ 199.0, 139.7, 138.7, 134.4, 131.9, 130.0, 129.7, 124.1, 121.6, 53.3, 21.4, 18.7.

HRMS (ESI-TOF) m/z: $[M + H]^+$ calculated for $C_{16}H_{16}B_rOS$ 335.0100; Found 335.0101.

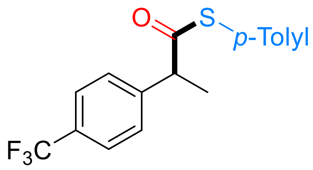

S-(*p*-tolyl)-2-(4-(trifluoromethyl)phenyl)propanethioate (3ga):

Yellow oil, 59.9 mg, 92% yield, R_f=0.2 (PE/EtOAc 40/1).

^{1}H NMR (400 MHz, $CDCl_3$) δ 7.60 (d, J = 8.2 Hz, 2H), 7.46 (d, J = 8.1 Hz, 2H), 7.22 (d, J = 8.1 Hz, 2H), 7.18 (d, J = 8.2 Hz, 2H), 4.05 (q, J = 7.1 Hz, 1H), 2.35 (s, 3H), 1.58 (d, J = 7.1 Hz, 3H).

^{13}C NMR (100 MHz, $CDCl_3$) δ 198.7, 143.6 (q, J = 1.1 Hz), 139.9, 134.4, 130.1, 129.8 (q, J = 32.5 Hz), 128.4, 125.7 (q, J = 3.7 Hz), 124.1 (q, J = 272.0 Hz), 123.9, 53.7, 21.3, 18.8.

^{19}F NMR (376 MHz, $CDCl_3$) δ -62.50.

HRMS (ESI-TOF) m/z: $[M + H]^+$ calculated for $C_{17}H_{16}F_3OS$ 325.0868; Found 325.0870.

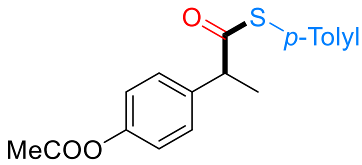

4-(1-oxo-1-(*p*-tolylthio)propan-2-yl)phenyl acetate (3ha):

Yellow oil, 59.7 mg, 95% yield, R_f=0.2 (PE/EtOAc 30/1).

^{1}H NMR (400 MHz, $CDCl_3$) δ 7.35 (d, J = 8.5 Hz, 2H), 7.22 (d, J = 8.2 Hz, 2H), 7.17 (d, J = 8.1 Hz, 2H), 7.07 (d, J = 8.5 Hz, 2H), 3.98 (q, J = 7.1 Hz, 1H), 2.34 (s, 3H), 2.28 (s, 3H), 1.55 (d, J =

7.1 Hz, 3H).

^{13}C NMR (100 MHz, $CDCl_3$) δ 199.3, 169.5, 150.1, 139.6, 137.1, 134.4, 130.0, 129.1, 124.2, 121.8, 53.3, 21.3, 21.2, 18.9.

HRMS (ESI-TOF) m/z: $[M + H]^+$ calculated for $C_{18}H_{19}O_3S$ 315.1049; Found 315.1054.

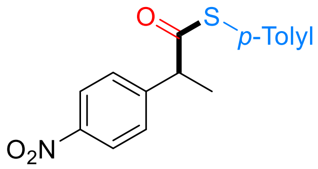

S-(*p*-tolyl)-2-(4-nitrophenyl)propanethioate (3ia):

Yellow oil, 48.5 mg, 81% yield, R_f=0.2 (PE/EtOAc 20/1).

^{1}H NMR (400 MHz, $CDCl_3$) δ 8.20 (d, J = 8.6 Hz, 2H), 7.52 (d, J = 8.7 Hz, 2H), 7.25 – 7.16 (m, 4H), 4.12 (q, J = 7.1 Hz, 1H), 2.35 (s, 3H), 1.61 (d, J = 7.1 Hz, 3H).

^{13}C NMR (100 MHz, $CDCl_3$) δ 198.2, 147.4, 146.9, 140.0, 134.4, 130.1, 128.9, 124.0, 123.5, 53.6, 21.3, 18.8.

HRMS (ESI-TOF) m/z: $[M + H]^+$ calculated for $C_{16}H_{16}NO_3S$ 302.0845; Found 302.0848.

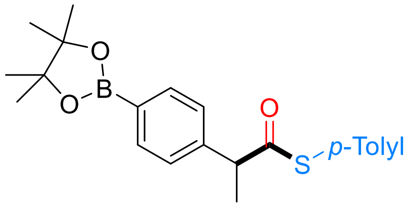

S-(*p*-tolyl)-2-(4-(4,4,5,5-tetramethyl-1,3,2-dioxaborolan-2-yl)phenyl)propanethioate (3ja):

Yellow oil, 54.7 mg, 72% yield, R_f=0.2 (PE/EtOAc 30/1).

^{1}H NMR (400 MHz, $CDCl_3$) δ 7.81 (d, J = 7.8 Hz, 2H), 7.36 (d, J = 7.8 Hz, 2H), 7.21 (d, J = 8.1 Hz, 2H), 7.16 (d, J = 8.1 Hz, 2H), 3.99 (q, J = 7.0 Hz, 1H), 2.33 (s, 3H), 1.55 (d, J = 7.0 Hz, 3H), 1.33 (s, 12H).

^{13}C NMR (100 MHz, $CDCl_3$) δ 199.2, 142.8, 139.6, 135.3, 134.5, 130.0, 127.5, 124.3, 83.8, 54.2, 24.9, 24.9, 21.3, 18.6.

HRMS (ESI-TOF) m/z: $[M + H]^+$ calculated for $C_{22}H_{28}BO_3S$ 382.1774; Found 382.1777.

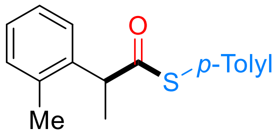

S-(*p*-tolyl)-2-(o-tolyl)propanethioate (3ka):

Colorless oil, 47.9 mg, 89% yield, R_f=0.2 (PE/EtOAc 40/1).

^{1}H NMR (400 MHz, $CDCl_3$) δ 7.32 (d, J = 5.7 Hz, 1H), 7.21 (d, J = 7.7 Hz, 5H), 7.18 – 7.14 (m, 2H), 4.20 (q, J = 7.0 Hz, 1H), 2.41 (s, 3H), 2.33 (s, 3H), 1.54 (d, J = 7.0 Hz, 3H).

^{13}C NMR (100 MHz, $CDCl_3$) δ 200.0, 139.5, 138.0, 136.3, 134.5, 130.7, 130.0, 127.5, 127.4, 126.6, 124.5, 50.0, 21.4, 19.9, 18.2.

HRMS (ESI-TOF) m/z: $[M + H]^+$ calculated for $C_{17}H_{19}OS$ 271.1151; Found 271.1154.

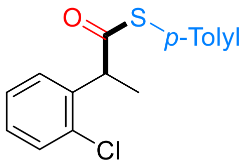

S-(*p*-tolyl)-2-(2-chlorophenyl)propanethioate (3la):

Colorless oil, 40.8 mg, 70% yield, R_f=0.2 (PE/EtOAc 40/1).

^{1}H NMR (400 MHz, $CDCl_3$) δ 7.44 – 7.36 (m, 2H), 7.26 (dd, J = 17.8, 7.3 Hz, 4H), 7.17 (d, J = 7.9 Hz, 2H), 4.52 (q, J = 7.1 Hz, 1H), 2.34 (s, 3H), 1.55 (d, J = 7.0 Hz, 3H).

^{13}C NMR (100 MHz, $CDCl_3$) δ 199.1, 139.6, 137.4, 134.5, 134.2, 130.0, 129.8, 129.0, 128.8, 127.3, 124.3, 50.0, 21.4, 17.9.

HRMS (ESI-TOF) m/z: $[M + H]^+$ calculated for $C_{16}H_{16}ClOS$ 291.0605; Found 291.0608.

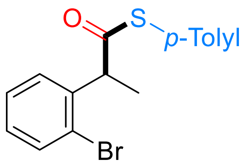

S-(*p*-tolyl)-2-(2-bromophenyl)propanethioate (3ma):

Colorless oil, 39.9 mg, 60% yield, R_f=0.2 (PE/EtOAc 40/1).

^{1}H NMR (400 MHz, $CDCl_3$) δ 7.60 (d, J = 8.0 Hz, 1H), 7.39 (dd, J = 7.8, 1.3 Hz, 1H), 7.31 (t, J = 7.5 Hz, 1H), 7.24 (d, J = 8.1 Hz, 2H), 7.20 – 7.12 (m, 3H), 4.52 (q, J = 7.0 Hz, 1H), 2.35 (s, 3H), 1.54 (d, J = 7.1 Hz, 3H).

^{13}C NMR (100 MHz, $CDCl_3$) δ 199.1, 139.6, 139.1, 134.5, 133.1, 130.0, 129.1, 127.9, 125.0, 124.2, 52.7, 21.4, 18.2.

HRMS (ESI-TOF) m/z: $[M + H]^+$ calculated for $C_{16}H_{16}B_rOS$ 335.0100; Found 335.0103.

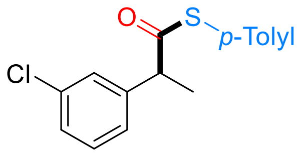

S-(*p*-tolyl)-2-(3-chlorophenyl)propanethioate (3na):

Colorless oil, 49.5 mg, 85% yield, R_f=0.2 (PE/EtOAc 40/1).

^{1}H NMR (400 MHz, $CDCl_3$) δ 7.34 (s, 1H), 7.26 (d, J = 4.6 Hz, 2H), 7.22 (d, J = 8.1 Hz, 3H), 7.18 (d, J = 8.1 Hz, 2H), 3.95 (q, J = 7.1 Hz, 1H), 2.34 (s, 3H), 1.55 (d, J = 7.1 Hz, 3H).

^{13}C NMR (100 MHz, $CDCl_3$) δ 198.9, 141.6, 139.8, 134.6, 134.4, 130.0, 130.0, 128.2, 127.8, 126.2, 124.0, 53.5, 21.4, 18.7.

HRMS (ESI-TOF) m/z: $[M + H]^+$ calculated for $C_{16}H_{16}ClOS$ 291.0605; Found 291.0606.

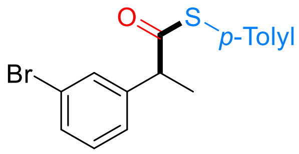

S-(*p*-tolyl)-2-(3-bromophenyl)propanethioate (3oa):

Colorless oil, 53.6 mg, 80% yield, R_f=0.2 (PE/EtOAc 40/1).

^{1}H NMR (400 MHz, $CDCl_3$) δ 7.49 (s, 1H), 7.41 (d, *J* = 7.8 Hz, 1H), 7.27 (d, *J* = 7.6 Hz, 1H), 7.24 – 7.16 (m, 5H), 3.94 (q, *J* = 7.1 Hz, 1H), 2.34 (s, 3H), 1.54 (d, *J* = 7.1 Hz, 3H).

^{13}C NMR (100 MHz, $CDCl_3$) δ 198.9, 141.9, 139.8, 134.4, 131.1, 130.7, 130.3, 130.0, 126.7, 124.0, 122.8, 53.5, 21.4, 18.8.

HRMS (ESI-TOF) m/z: $[M + H]^+$ calculated for $C_{16}H_{16}B_rOS$ 335.0100; Found 335.0104.

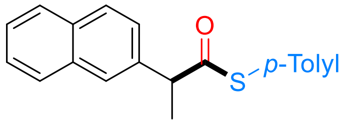

S-(*p*-tolyl)-2-(naphthalen-2-yl)propanethioate (3pa):

Colorless oil, 47.3 mg, 77% yield, R_f=0.2 (PE/EtOAc 40/1).

^{1}H NMR (400 MHz, $CDCl_3$) δ 7.89 – 7.75 (m, 4H), 7.52 – 7.39 (m, 3H), 7.21 (d, J = 8.1 Hz, 2H), 7.15 (d, J = 8.1 Hz, 2H), 4.15 (q, J = 7.0 Hz, 1H), 2.33 (s, 3H), 1.64 (d, J = 7.1 Hz, 3H).

^{13}C NMR (100 MHz, $CDCl_3$) δ 199.5, 139.6, 137.2, 134.5, 133.5, 132.8, 130.0, 128.5, 128.0, 127.7, 127.0, 126.3, 126.1, 126.0, 124.4, 54.2, 21.4, 18.8.

HRMS (ESI-TOF) m/z: $[M + H]^+$ calculated for $C_{20}H_{19}OS$ 307.1151; Found 307.1154.

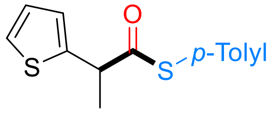

S-(*p*-tolyl)-2-(thiophen-2-yl)propanethioate (3qa):

Light yellow oil, 35.2 mg, 67% yield, R_f=0.2 (PE/EtOAc 30/1).

^{1}H NMR (400 MHz, $CDCl_3$) δ 7.25 (d, J = 8.0 Hz, 3H), 7.18 (d, J = 7.9 Hz, 2H), 7.03 (d, J = 2.6 Hz, 1H), 7.01 – 6.97 (m, 1H), 4.25 (q, J = 7.1 Hz, 1H), 2.35 (s, 3H), 1.65 (d, J = 7.1 Hz, 3H).

^{13}C NMR (100 MHz, $CDCl_3$) δ 198.7, 142.0, 139.7, 134.4, 130.0, 126.9, 125.9, 125.1, 124.2, 49.0, 21.4, 19.7.

HRMS (ESI-TOF) m/z: $[M + H]^+$ calculated for $C_{14}H_{15}OS_2$ 263.0559; Found 263.0561.

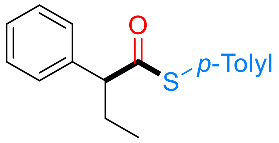

S-(*p*-tolyl)-2-phenylbutanethioate (3ra):

Colorless oil, 30.6 mg, 57% yield, R_f=0.2 (PE/EtOAc 40/1).

^{1}H NMR (400 MHz, $CDCl_3$) δ 7.33 (d, J = 4.2 Hz, 4H), 7.31 – 7.26 (m, 1H), 7.22 (d, J = 8.1 Hz, 2H), 7.17 (d, J = 8.0 Hz, 2H), 3.73 (t, J = 7.5 Hz, 1H), 2.34 (s, 3H), 2.23 – 2.15 (m, 1H), 1.91 – 1.81 (m, 1H), 0.93 (t, J = 7.4 Hz, 3H).

^{13}C NMR (100 MHz, $CDCl_3$) δ 198.9, 139.6, 138.3, 134.4, 129.9, 128.7, 128.4, 127.5, 124.4, 61.8, 26.9, 21.3, 12.1.

HRMS (ESI-TOF) m/z: $[M + H]^+$ calculated for $C_{17}H_{19}OS$ 271.1151; Found 271.1155.

6. NMR Spectra of the Products.

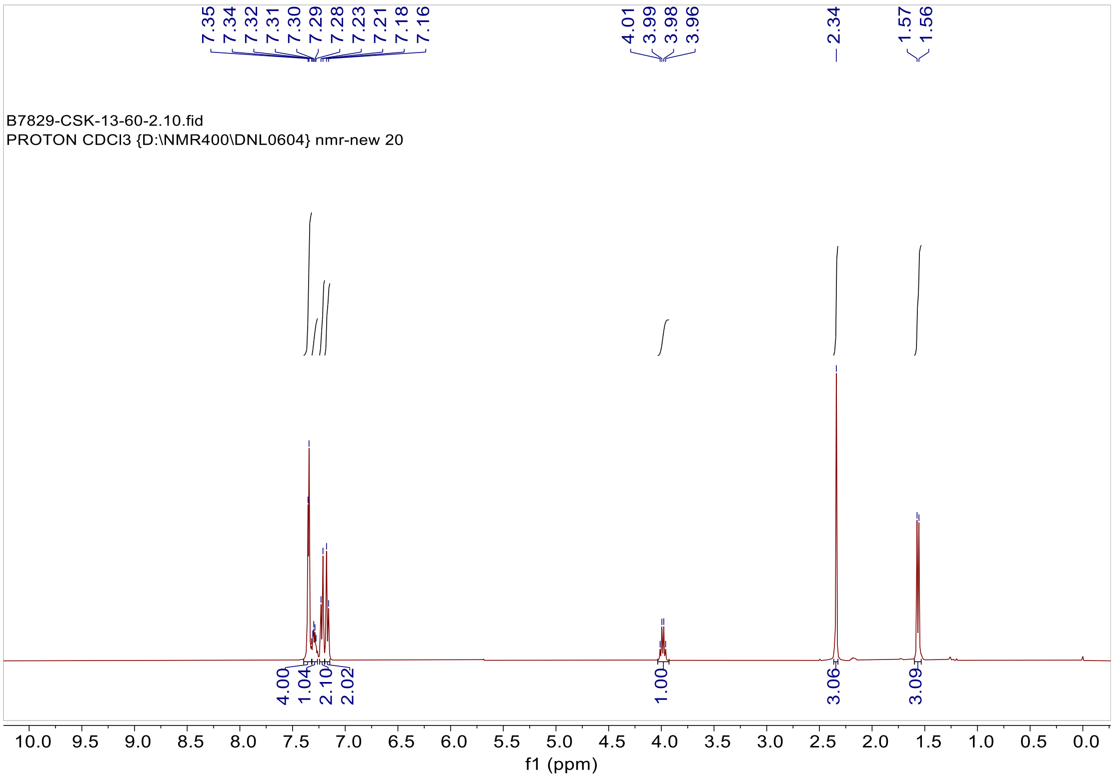

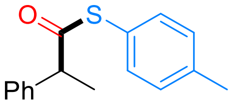

3aa

^{1}H NMR (400 MHz, $CDCl_3$)
^{13}C NMR (100 MHz, $CDCl_3$)

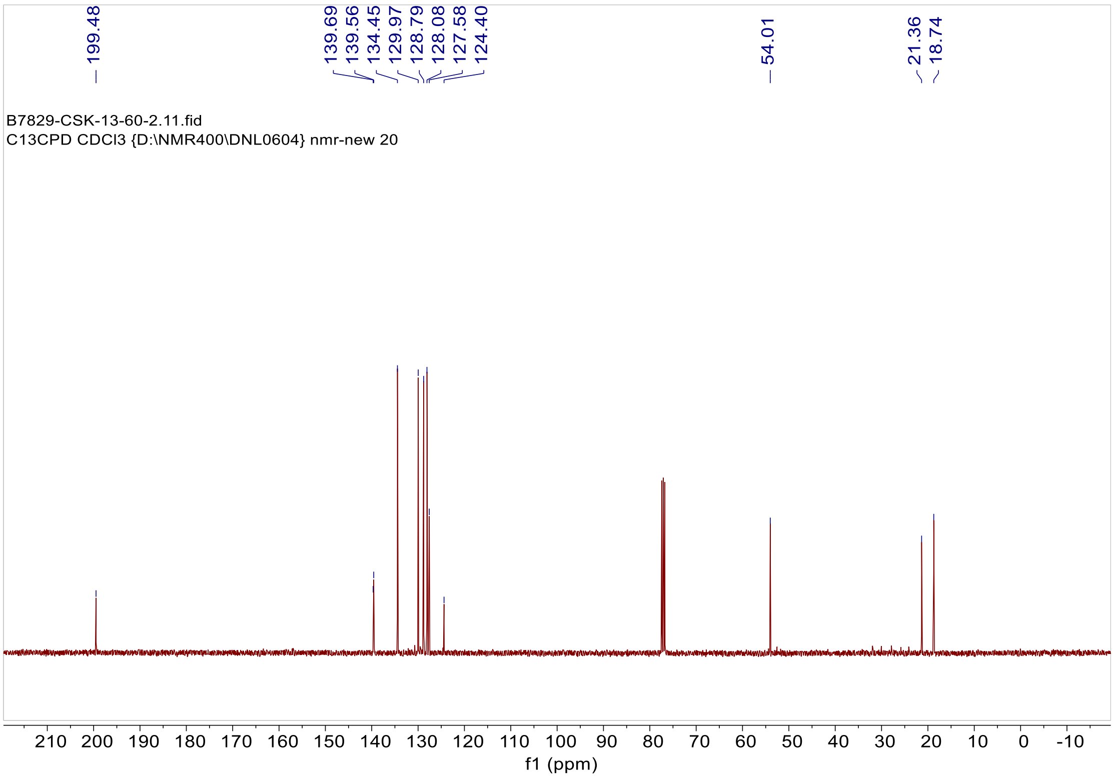

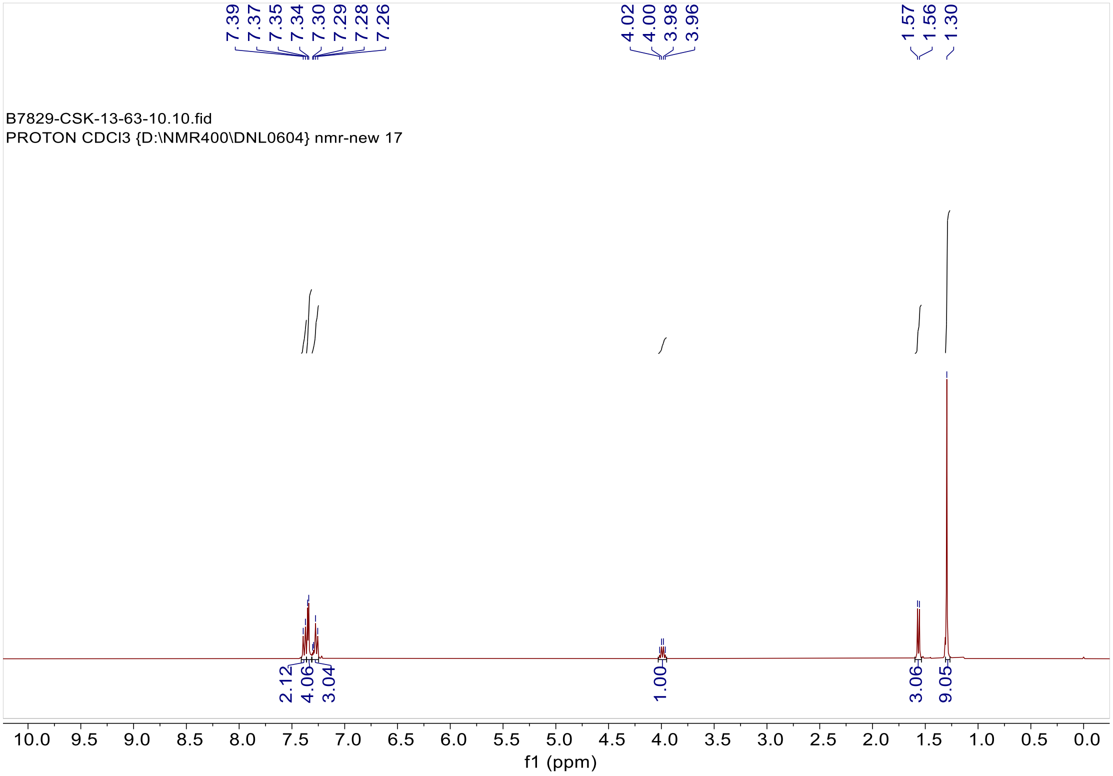

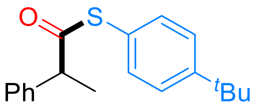

3ab

^{1}H NMR (400 MHz, $CDCl_3$)
^{13}C NMR (100 MHz, $CDCl_3$)

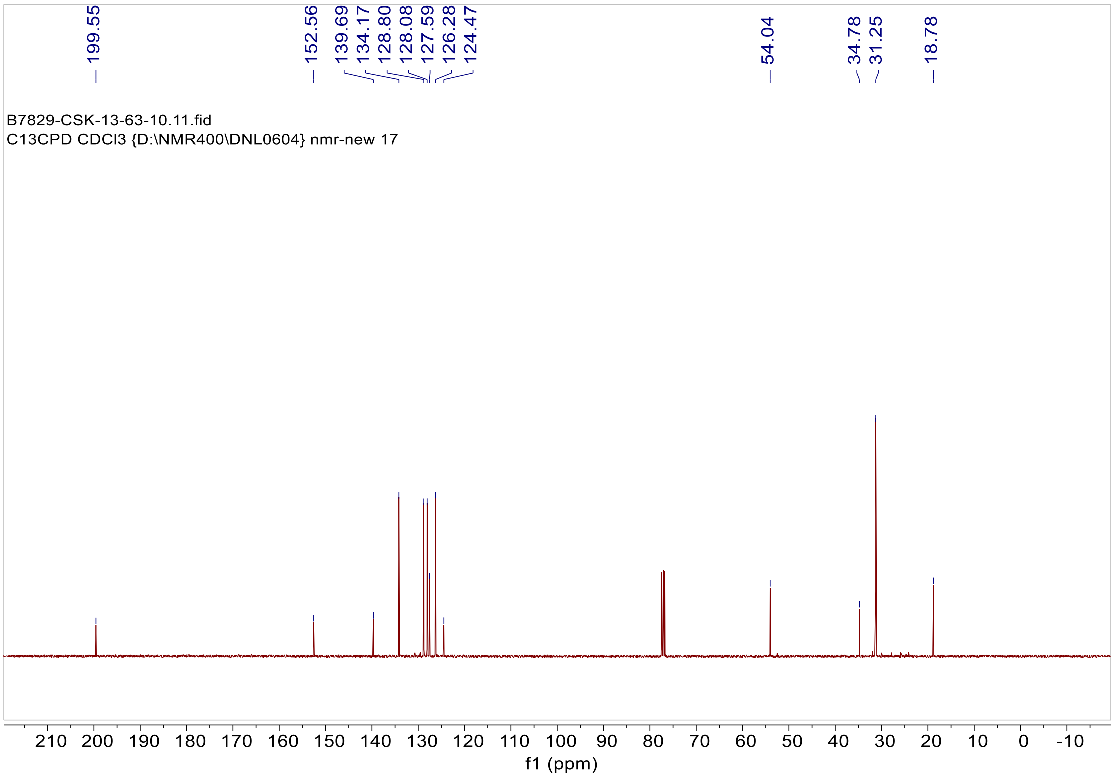

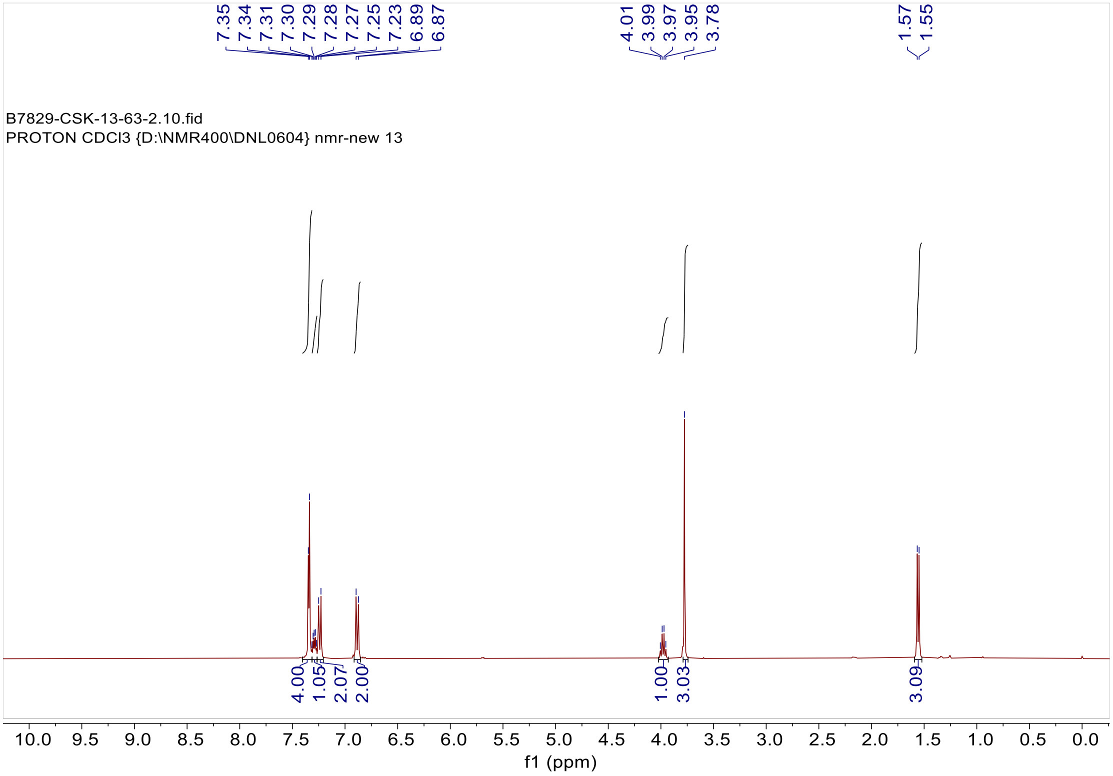

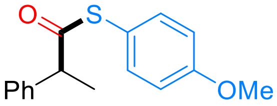

3ac

^{1}H NMR (400 MHz, $CDCl_3$)

^{13}C NMR (100 MHz, $CDCl_3$)

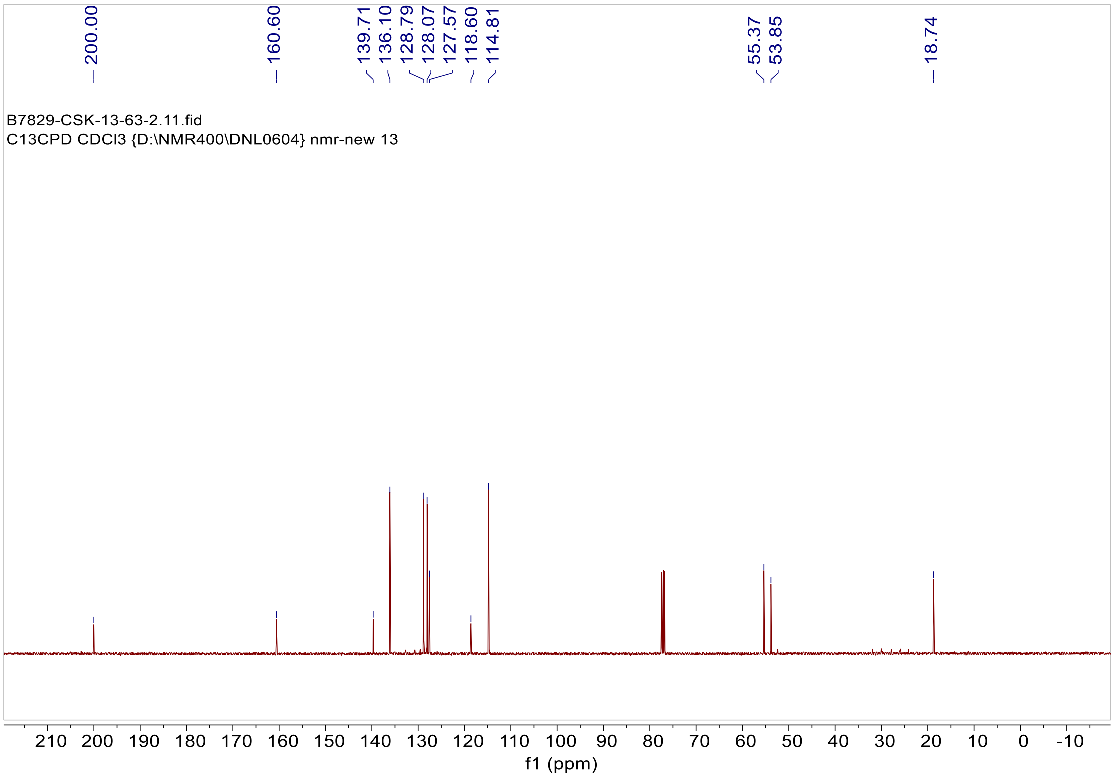

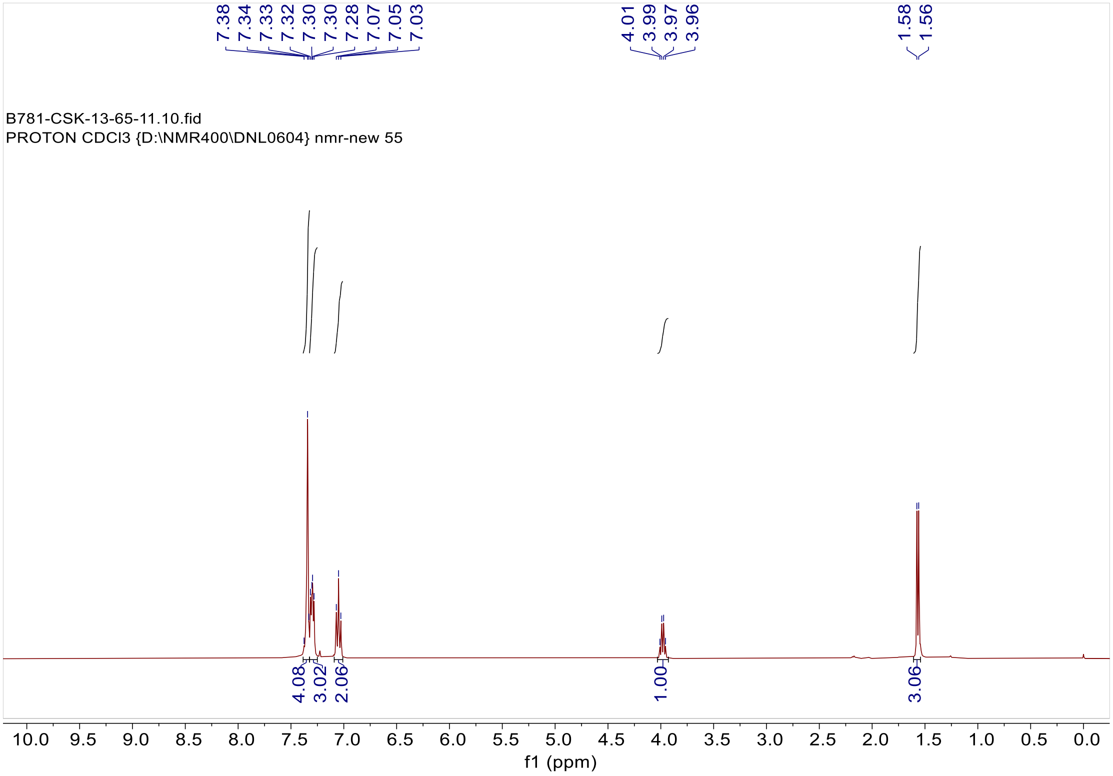

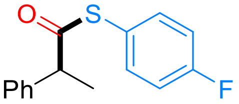

3ad

^{1}H NMR (400 MHz, $CDCl_3$)

^{13}C NMR (100 MHz, $CDCl_3$)

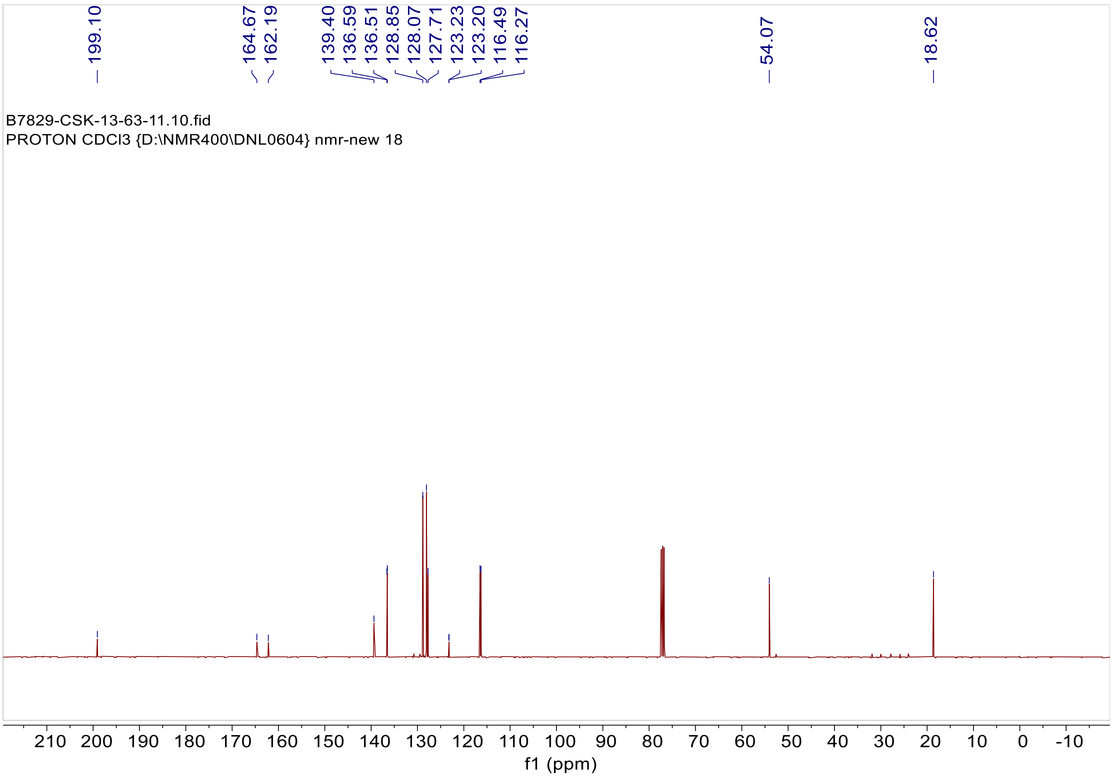

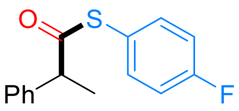

3ad

^{19}F NMR (376 MHz, $CDCl_3$)

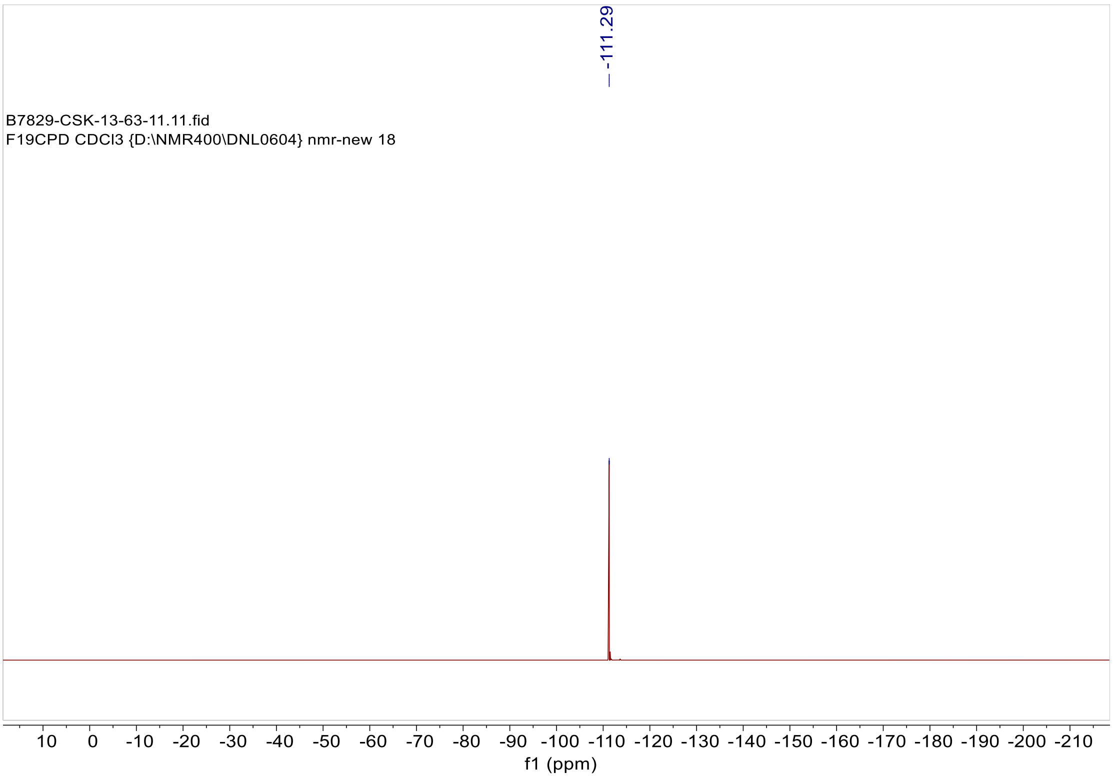

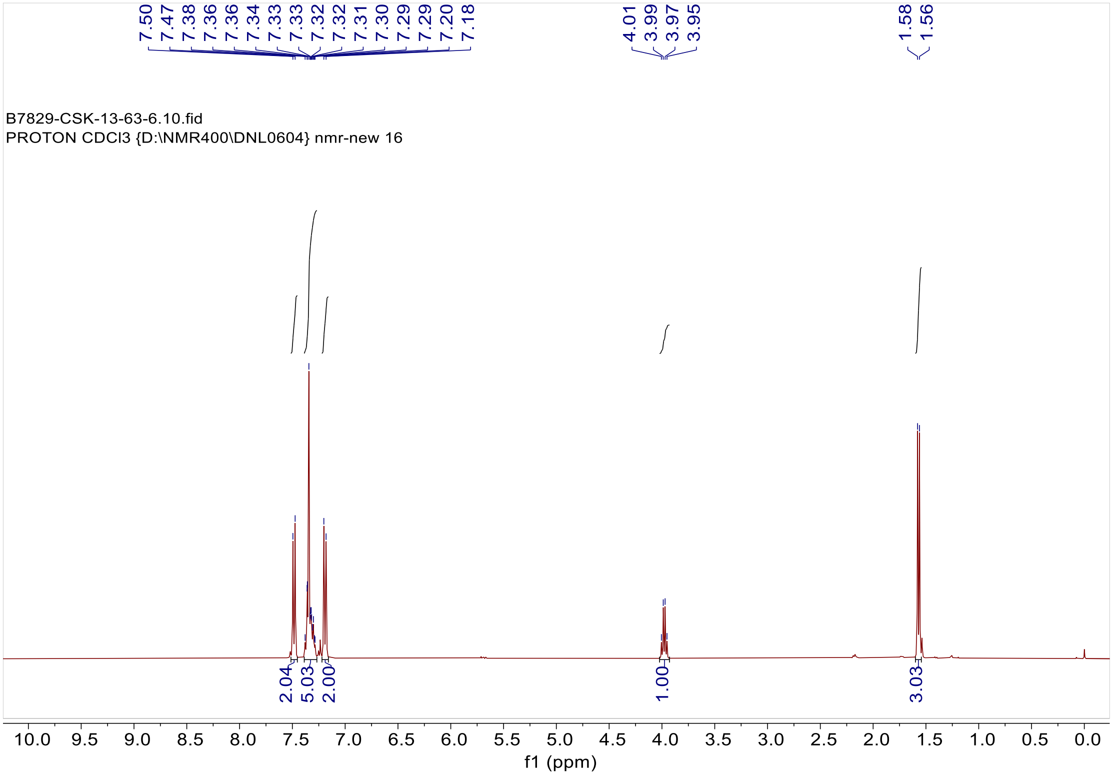

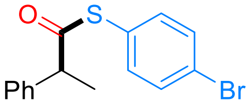

3ae

^{1}H NMR (400 MHz, $CDCl_3$)
^{13}C NMR (100 MHz, $CDCl_3$)

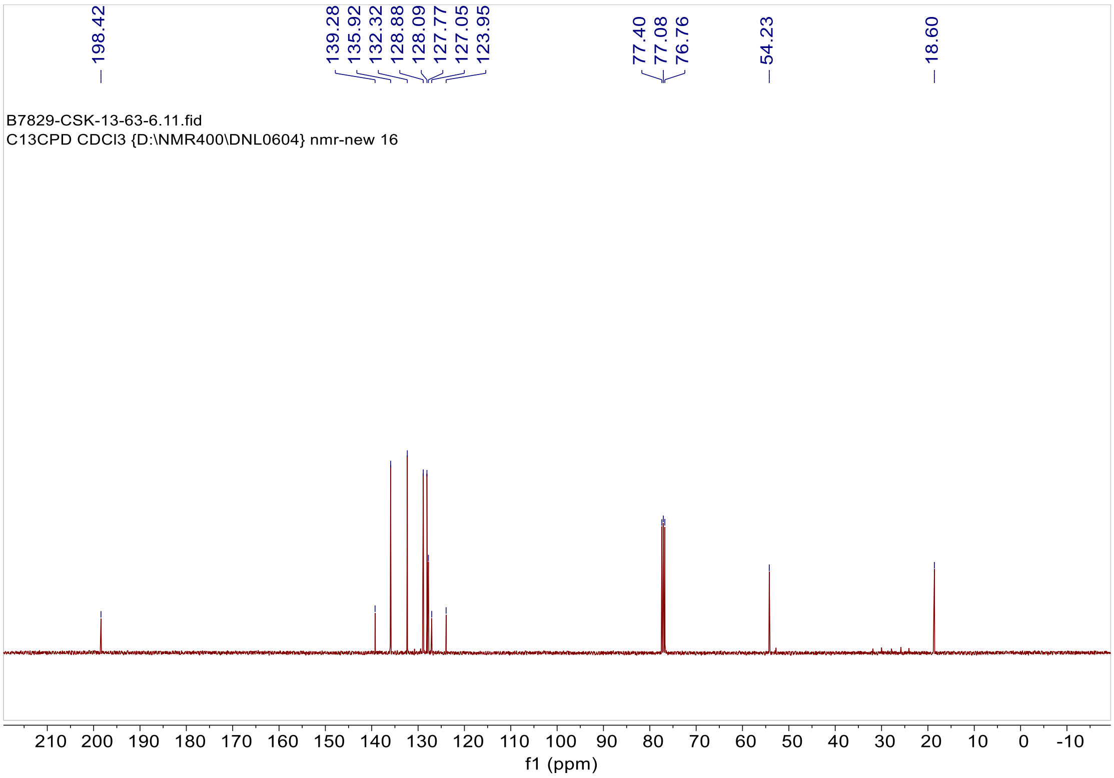

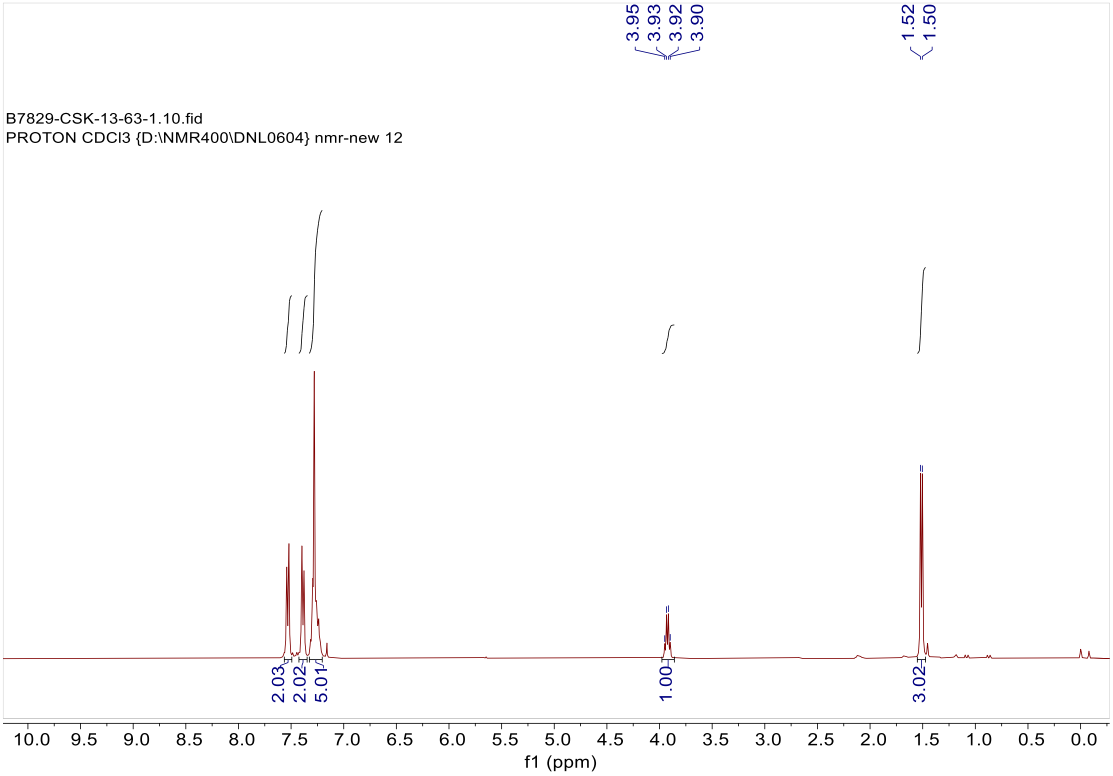

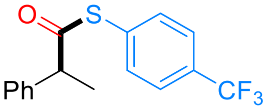

3af

^{1}H NMR (400 MHz, $CDCl_3$)

^{13}C NMR (100 MHz, $CDCl_3$)

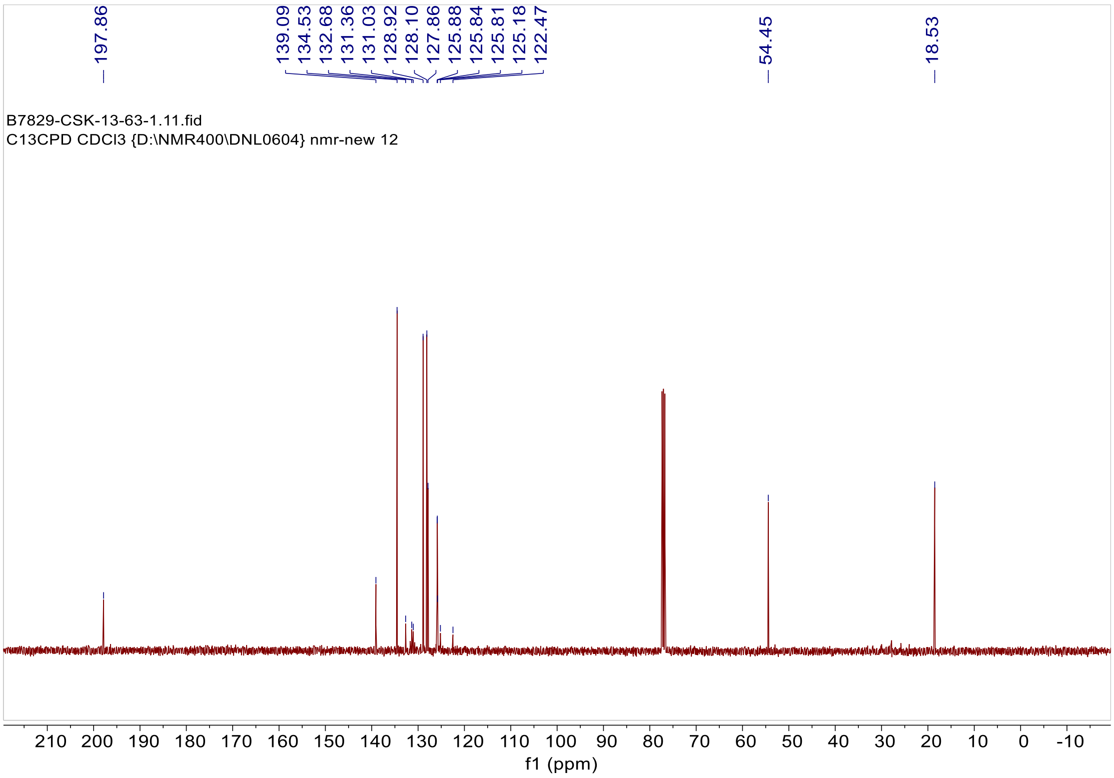

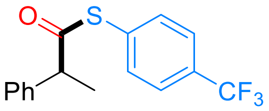

3af ^{19}F NMR (376 MHz, $CDCl_3$)

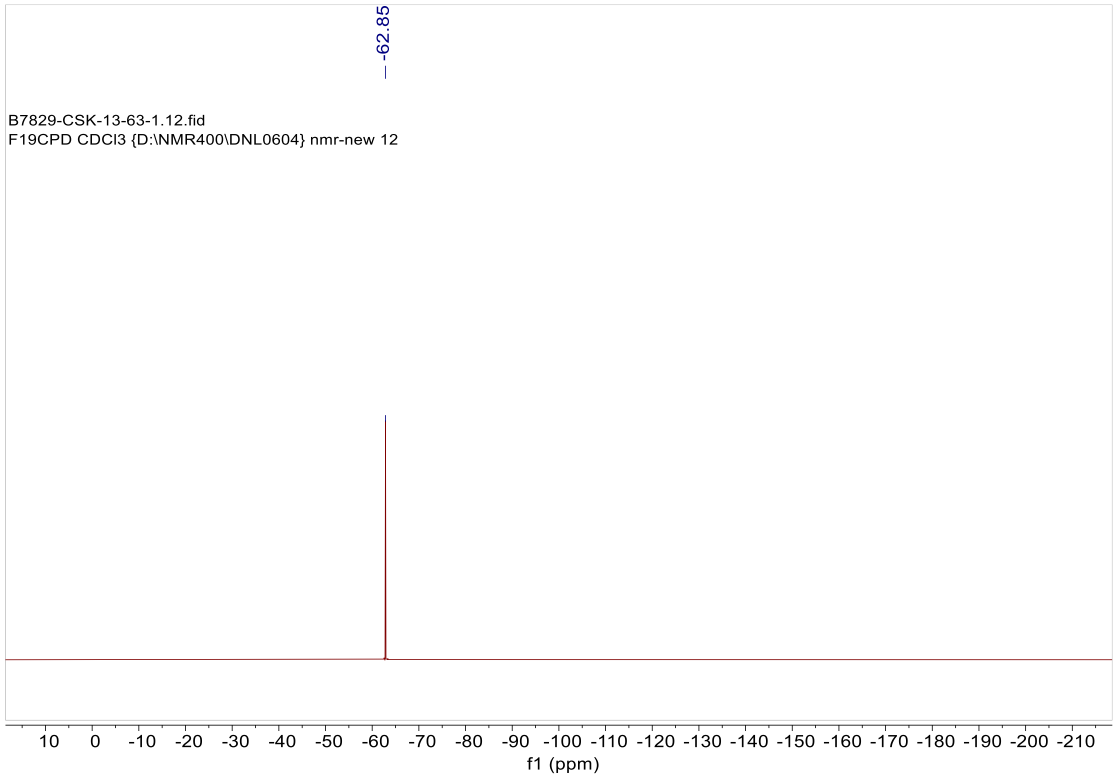

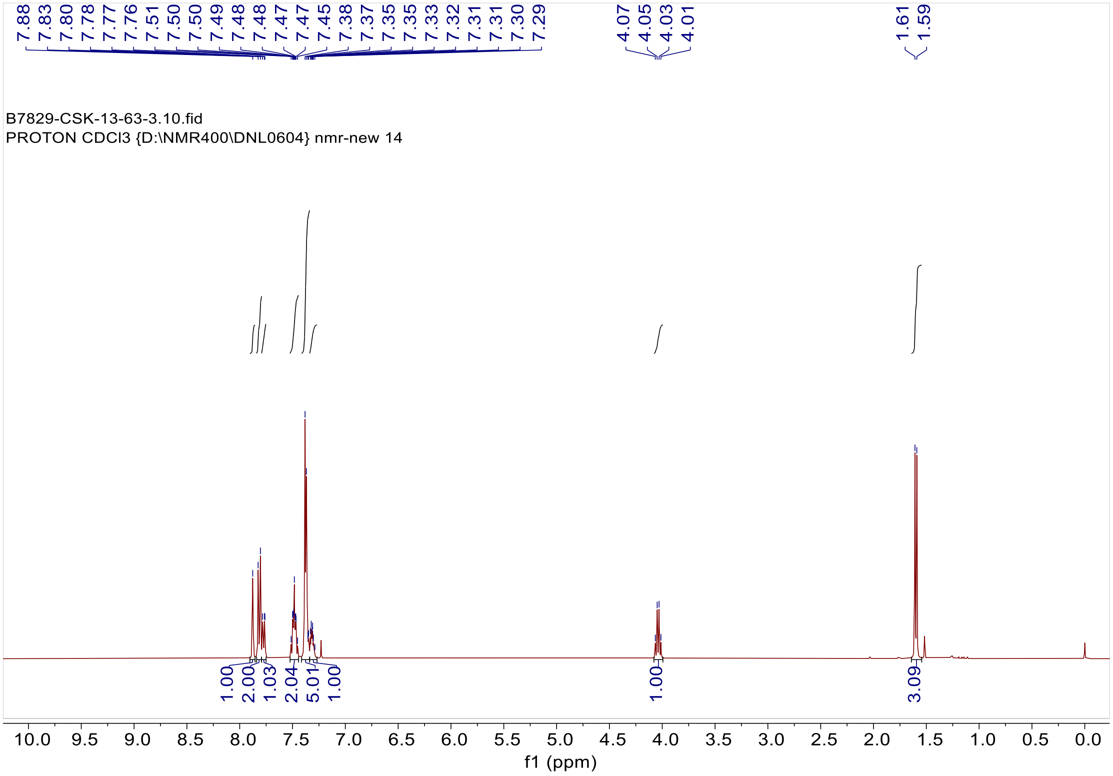

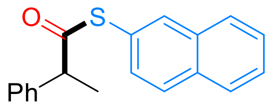

3ag

^{1}H NMR (400 MHz, $CDCl_3$)

^{13}C NMR (100 MHz, $CDCl_3$)

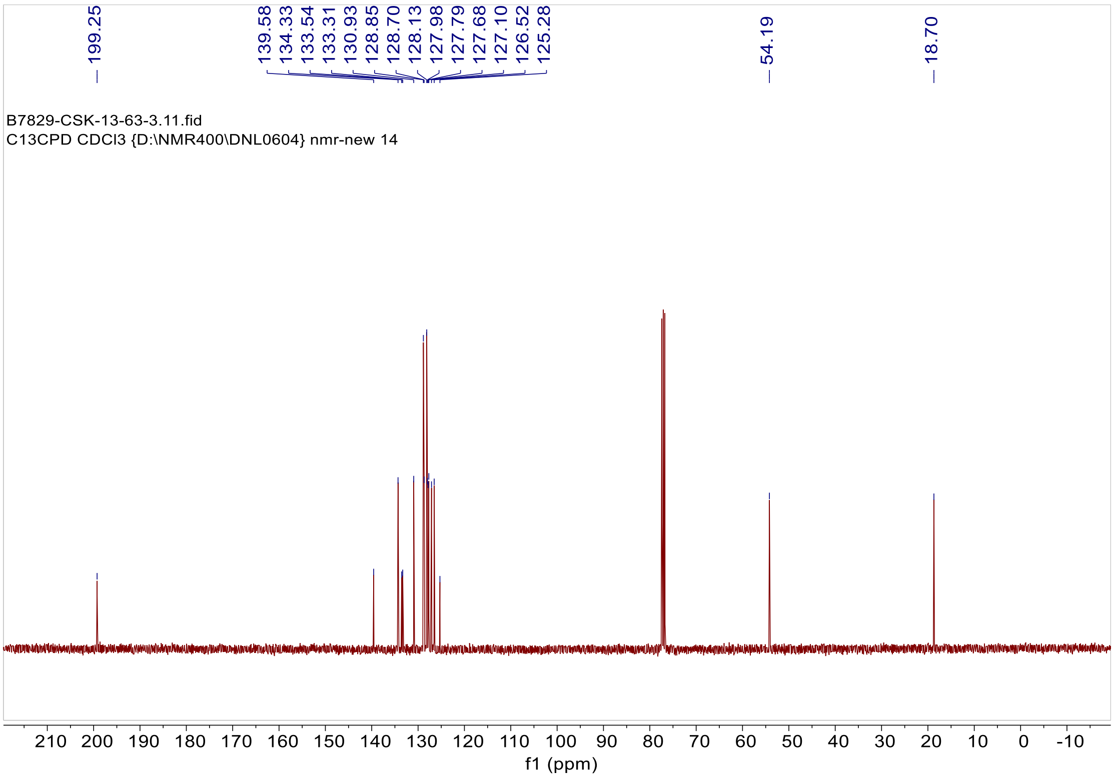

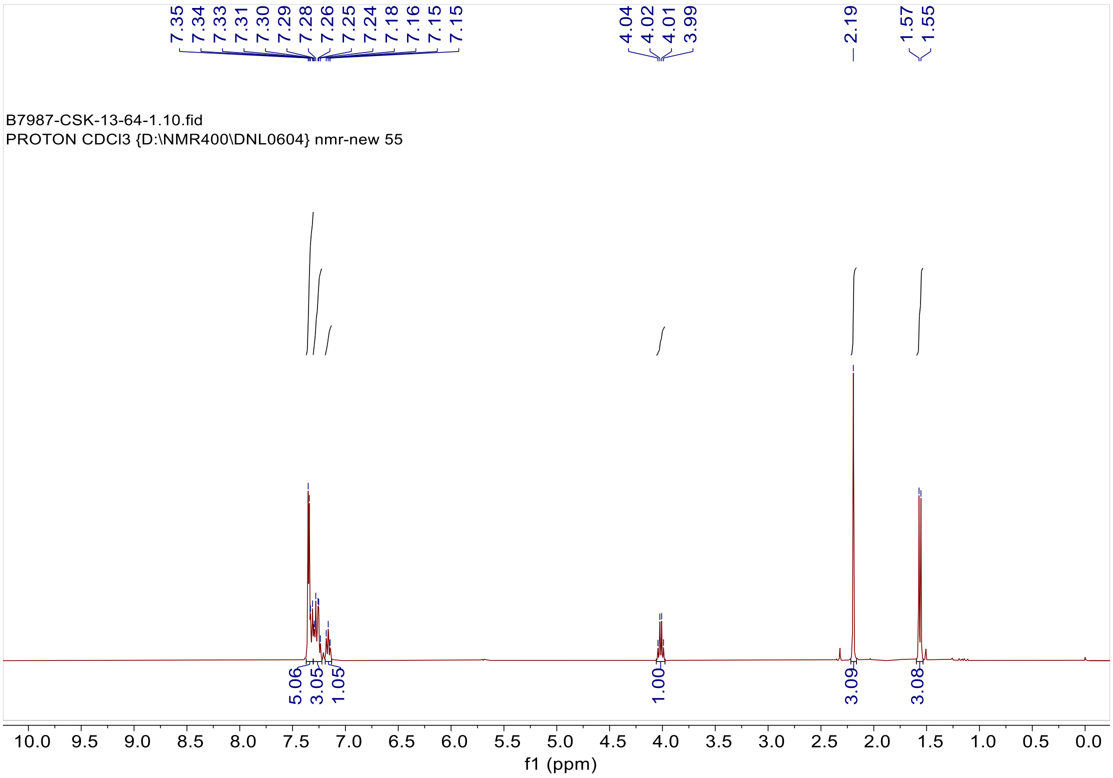

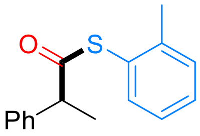

3ah

^{1}H NMR (400 MHz, $CDCl_3$)
^{13}C NMR (100 MHz, $CDCl_3$)

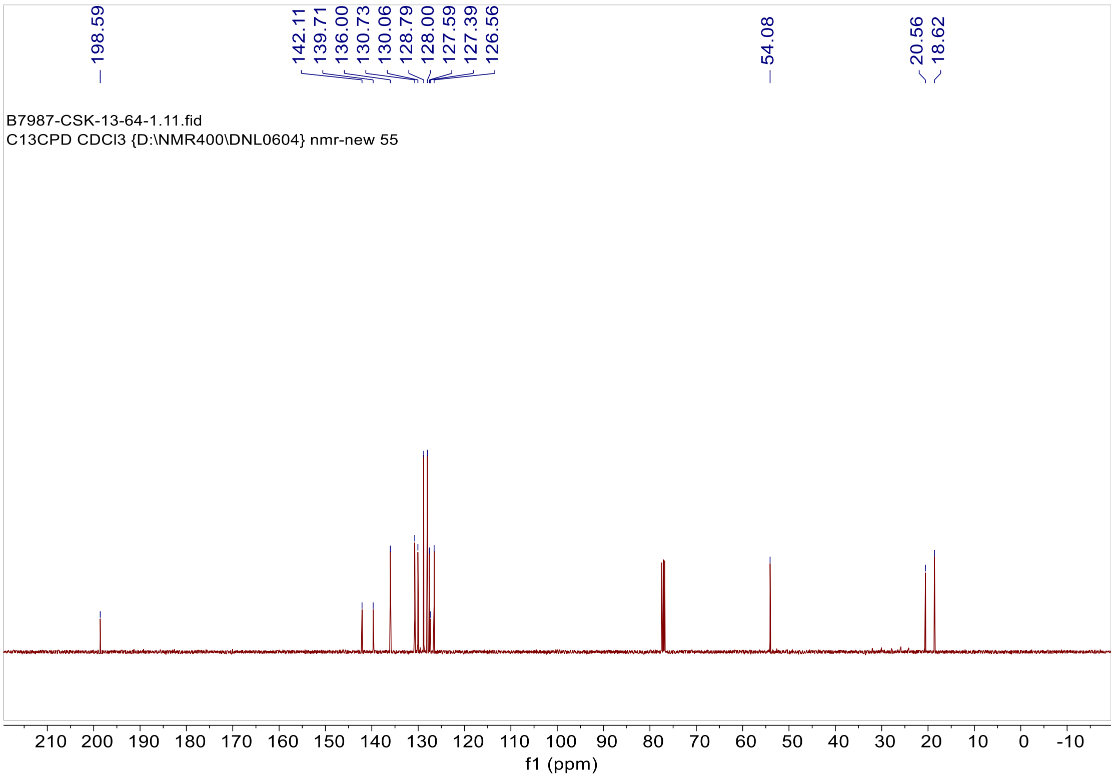

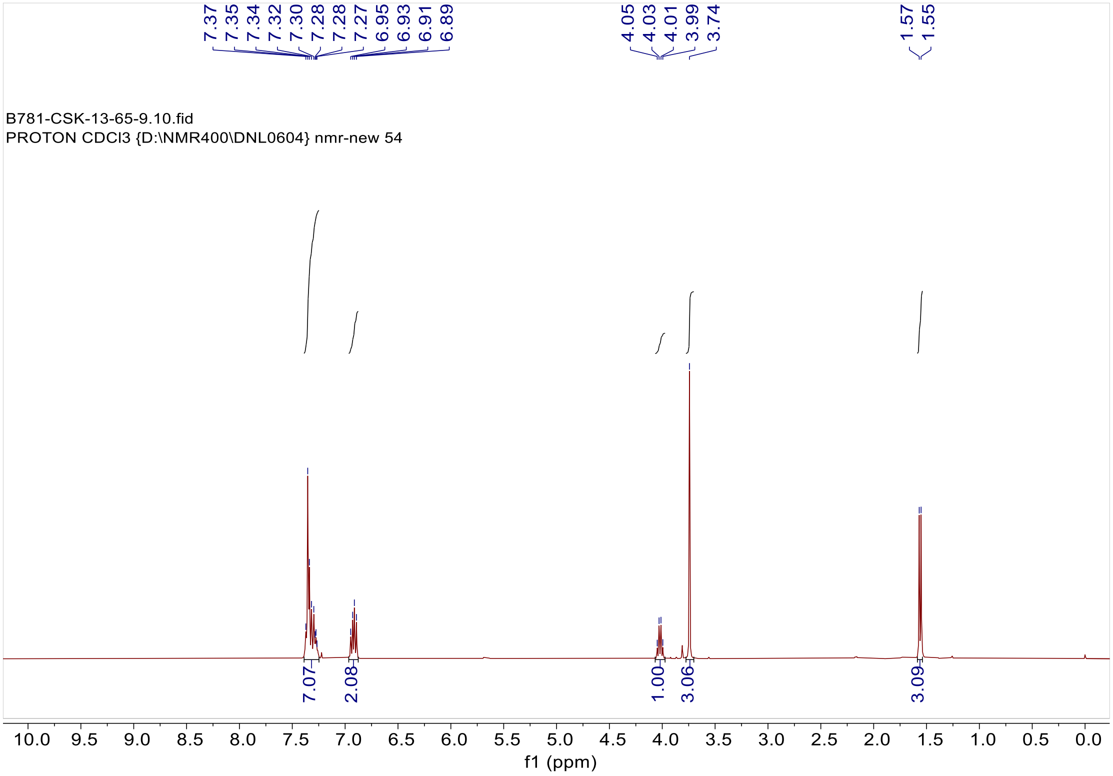

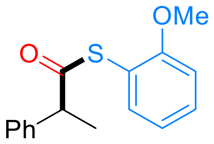

3ai

^{1}H NMR (400 MHz, $CDCl_3$)

^{13}C NMR (100 MHz, $CDCl_3$)

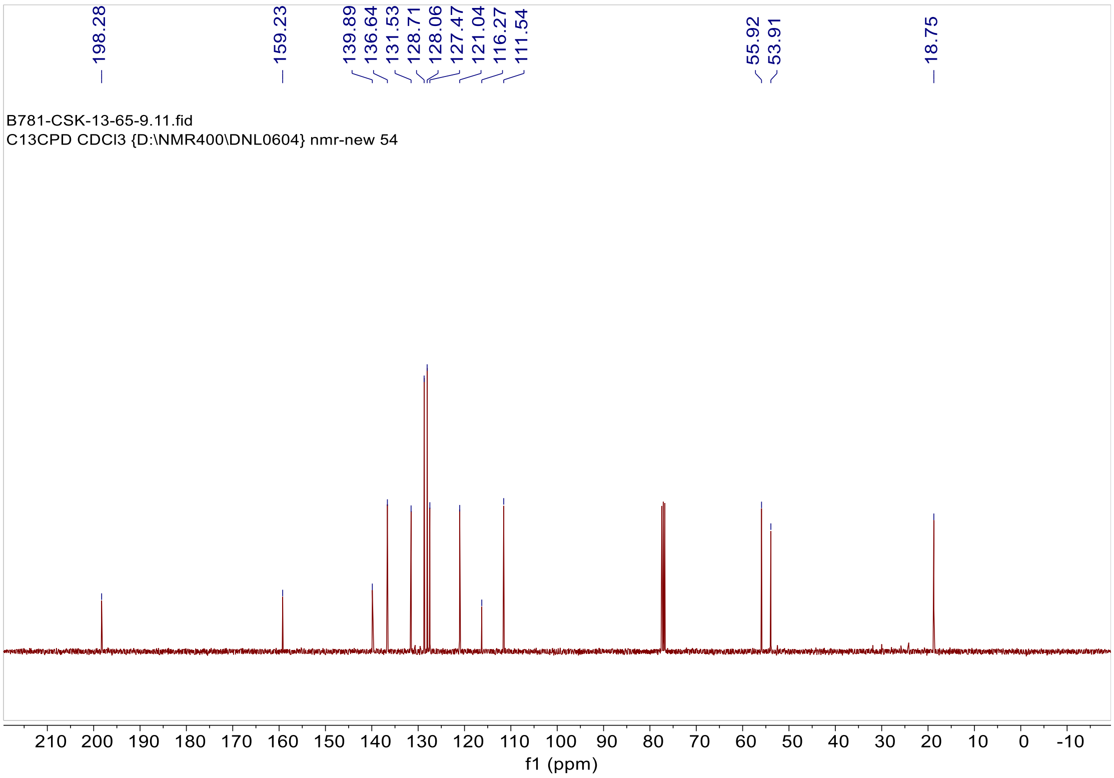

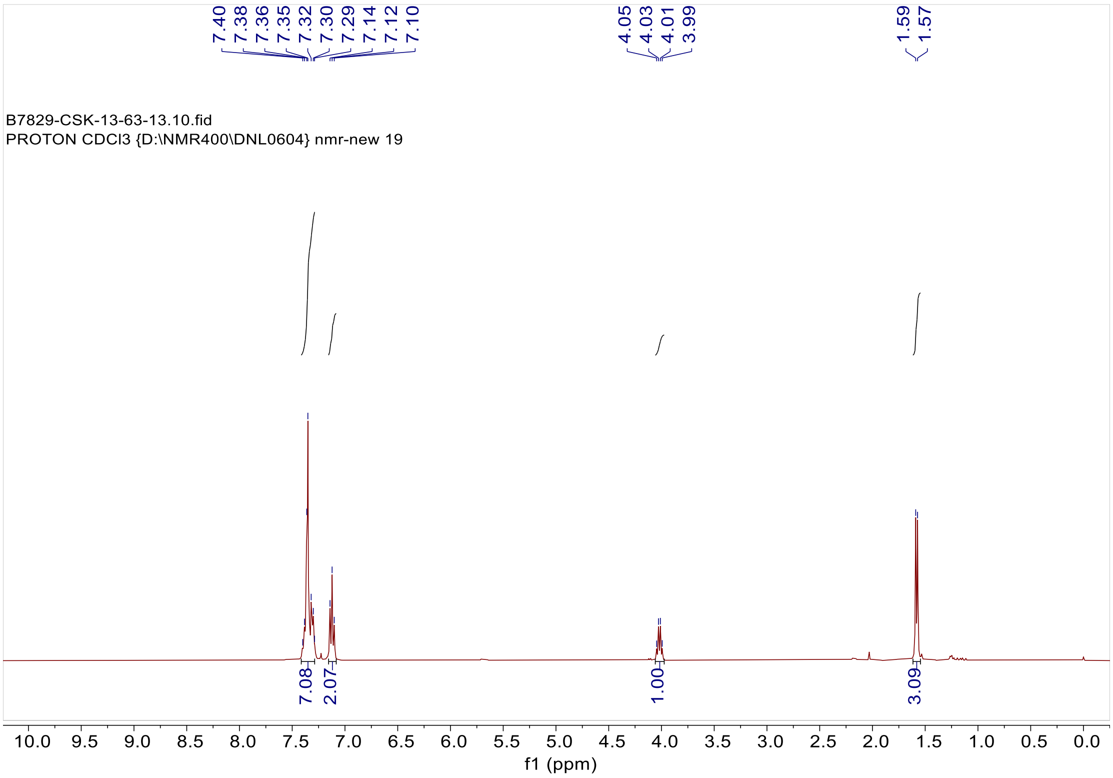

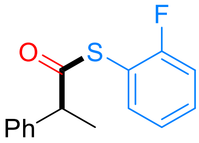

3aj

^{1}H NMR (400 MHz, $CDCl_3$)

^{13}C NMR (100 MHz, $CDCl_3$)

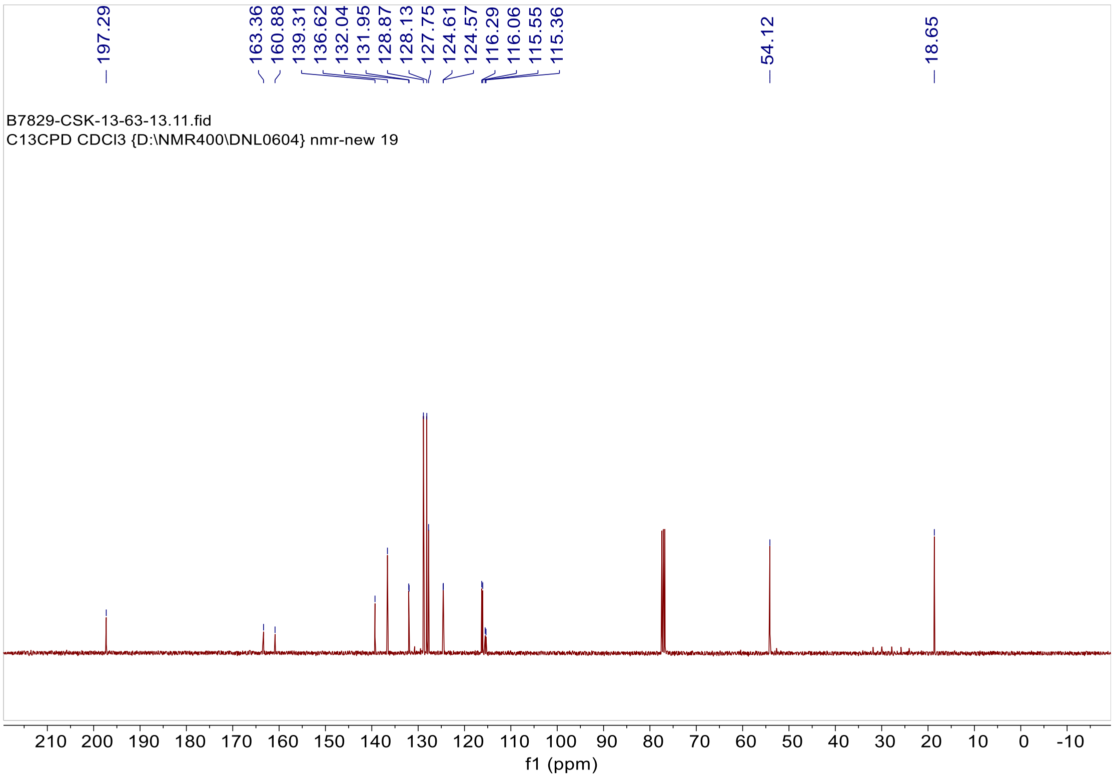

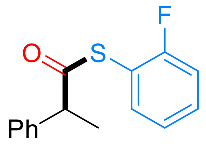

3aj ^{19}F NMR (376 MHz, $CDCl_3$)

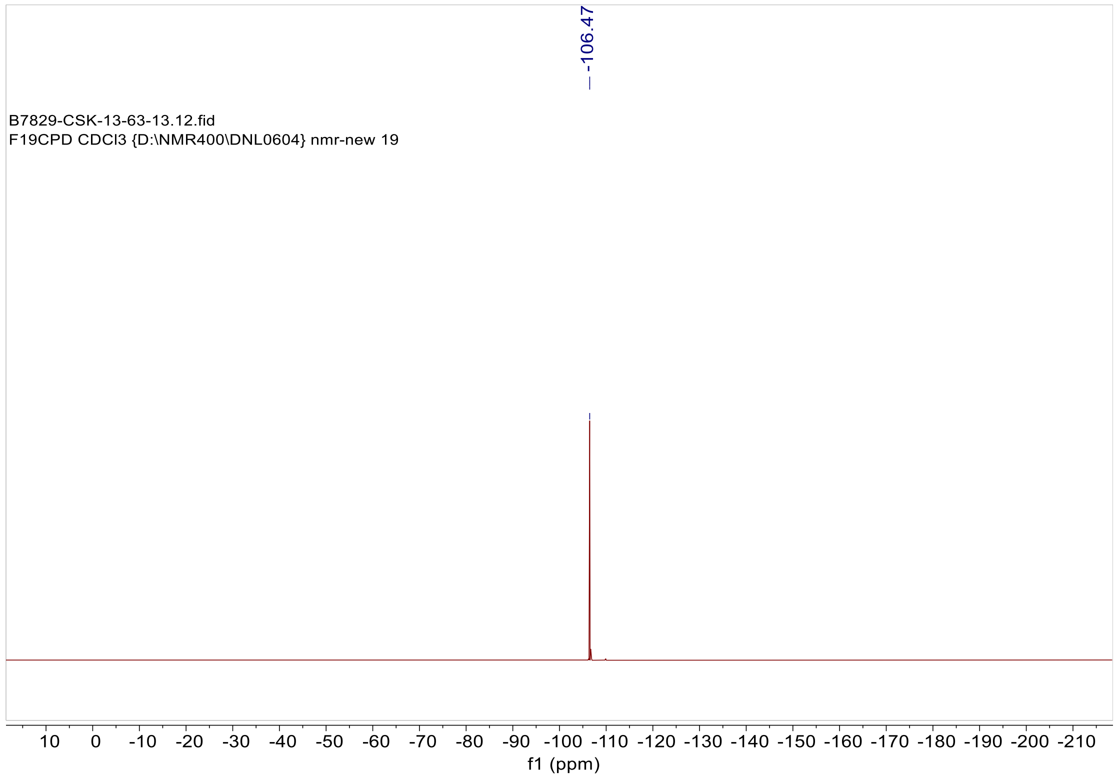

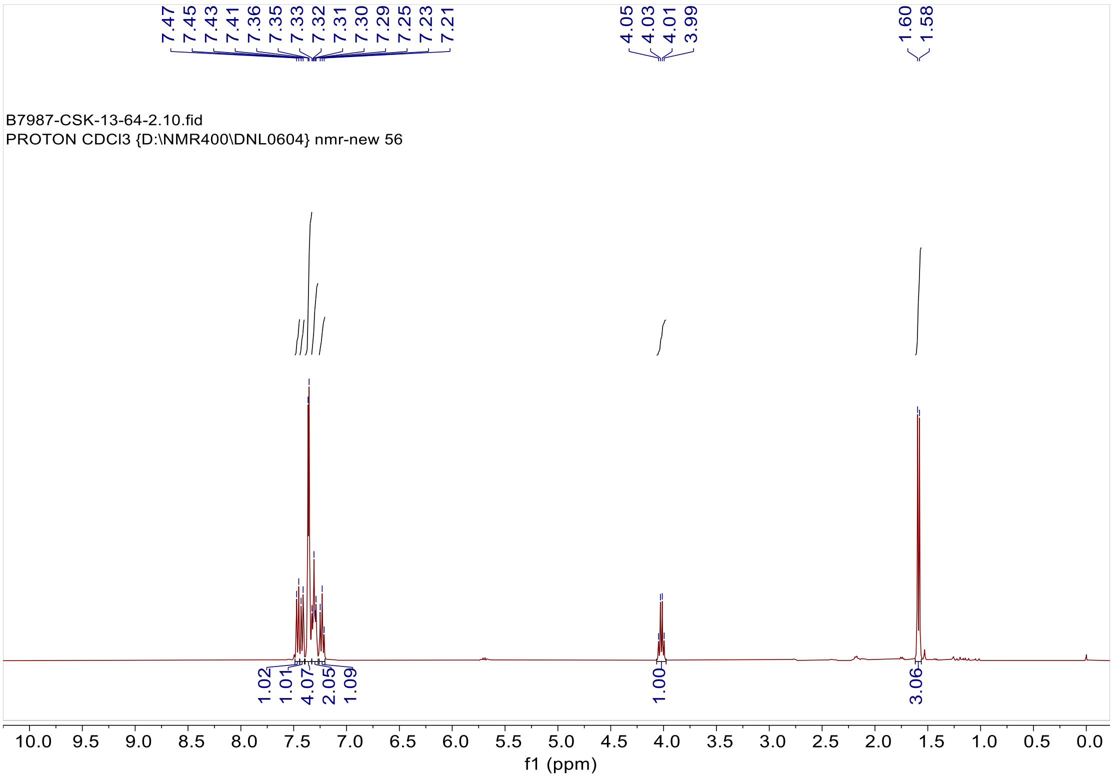

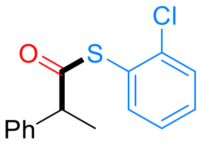

3ak

^{1}H NMR (400 MHz, $CDCl_3$)
^{13}C NMR (100 MHz, $CDCl_3$)

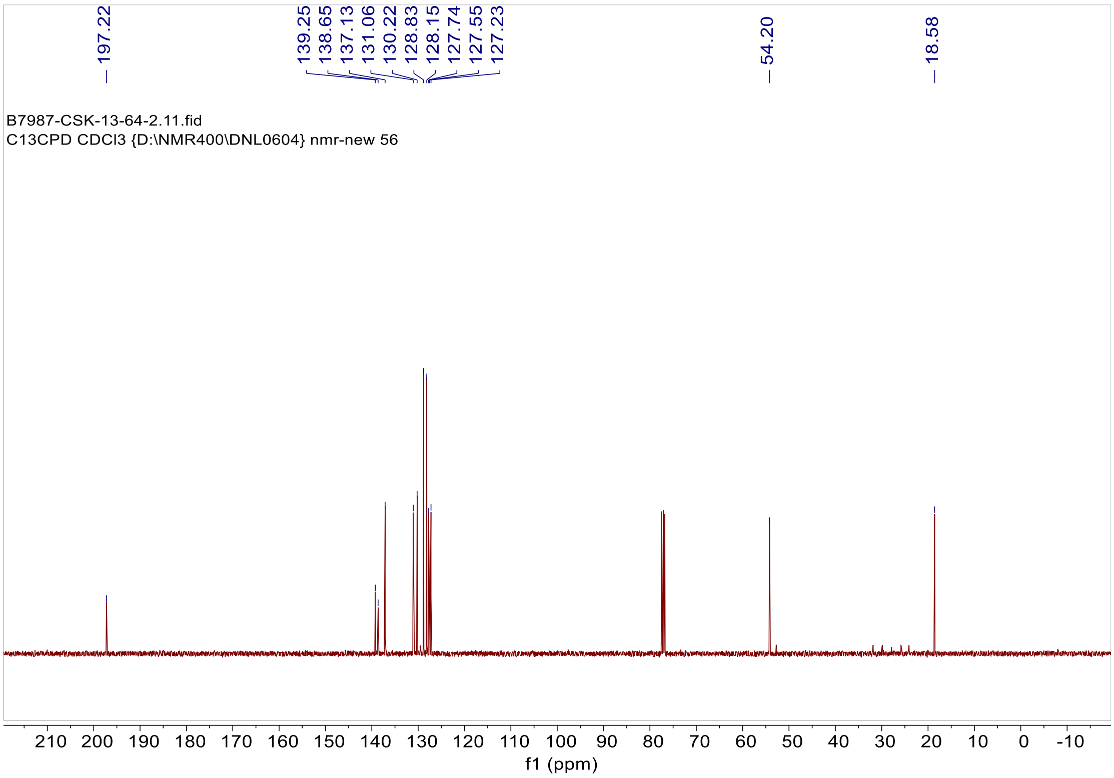

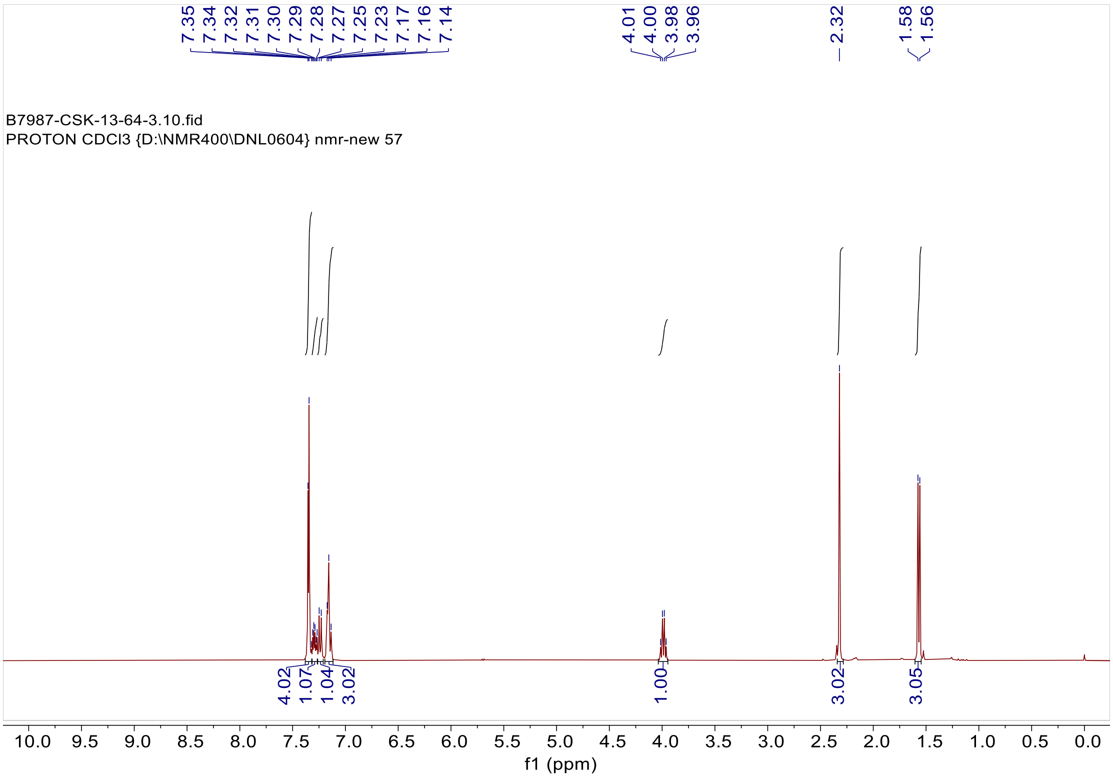

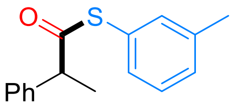

3am

^{1}H NMR (400 MHz, $CDCl_3$)

^{13}C NMR (100 MHz, $CDCl_3$)

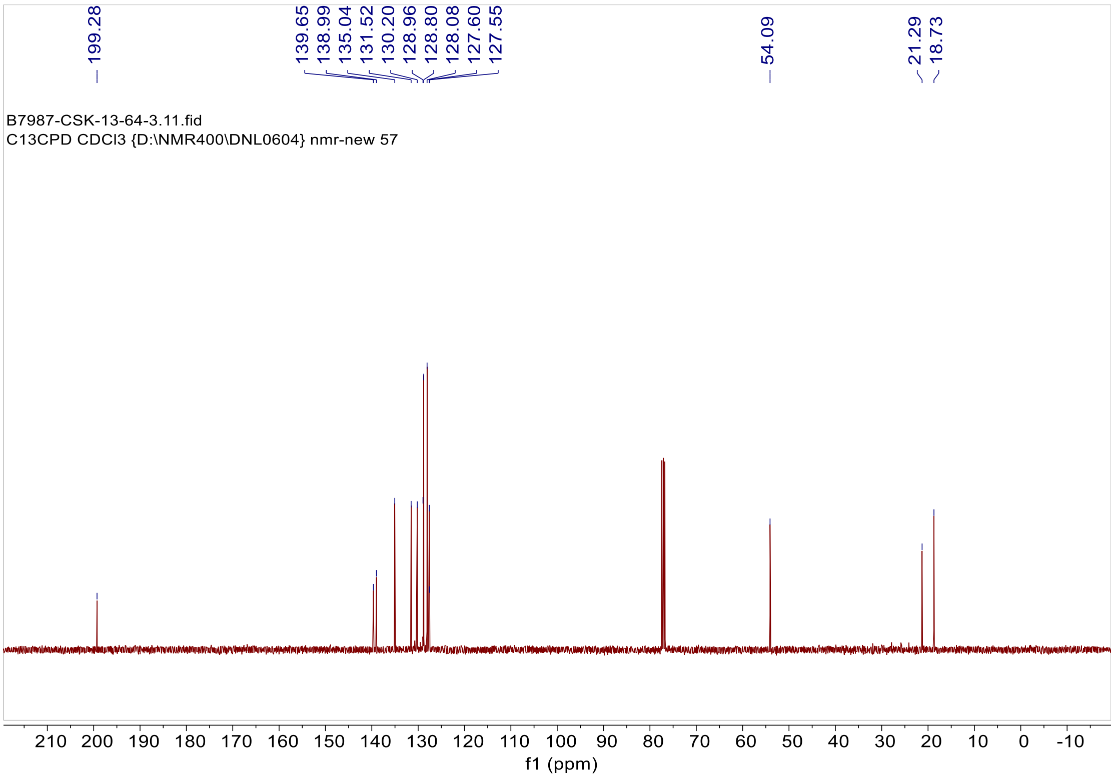

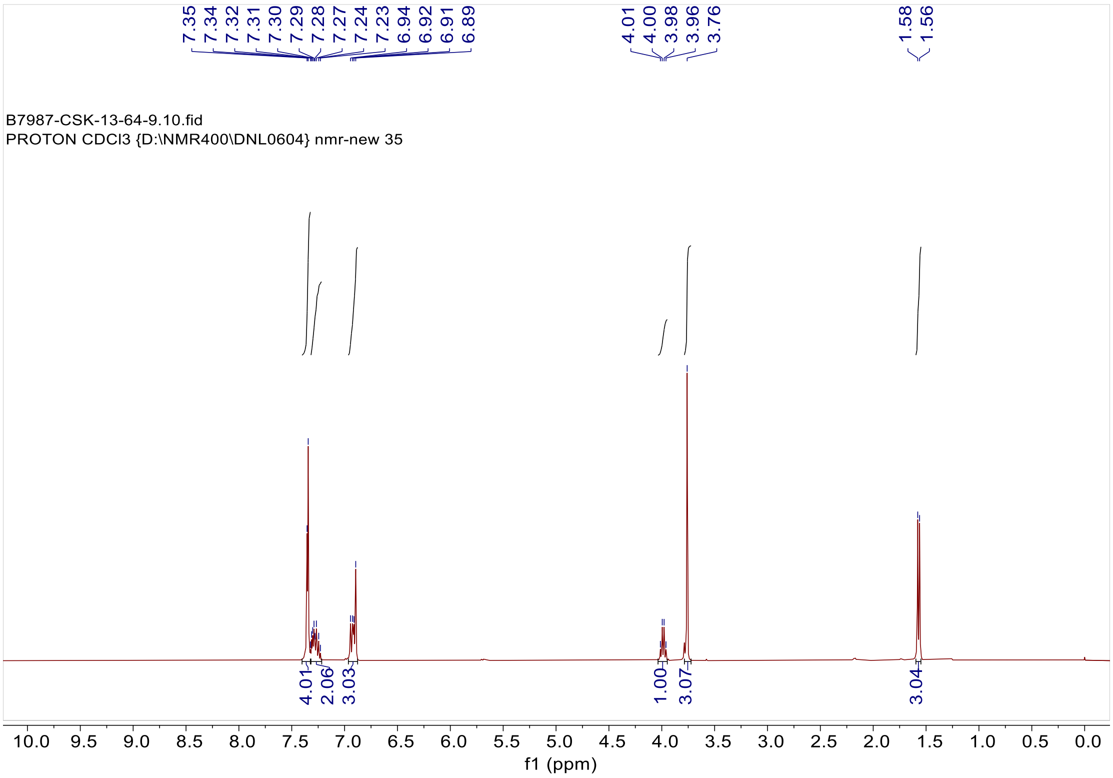

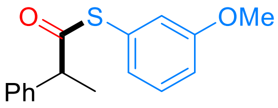

3an

^{1}H NMR (400 MHz, $CDCl_3$)
^{13}C NMR (100 MHz, $CDCl_3$)

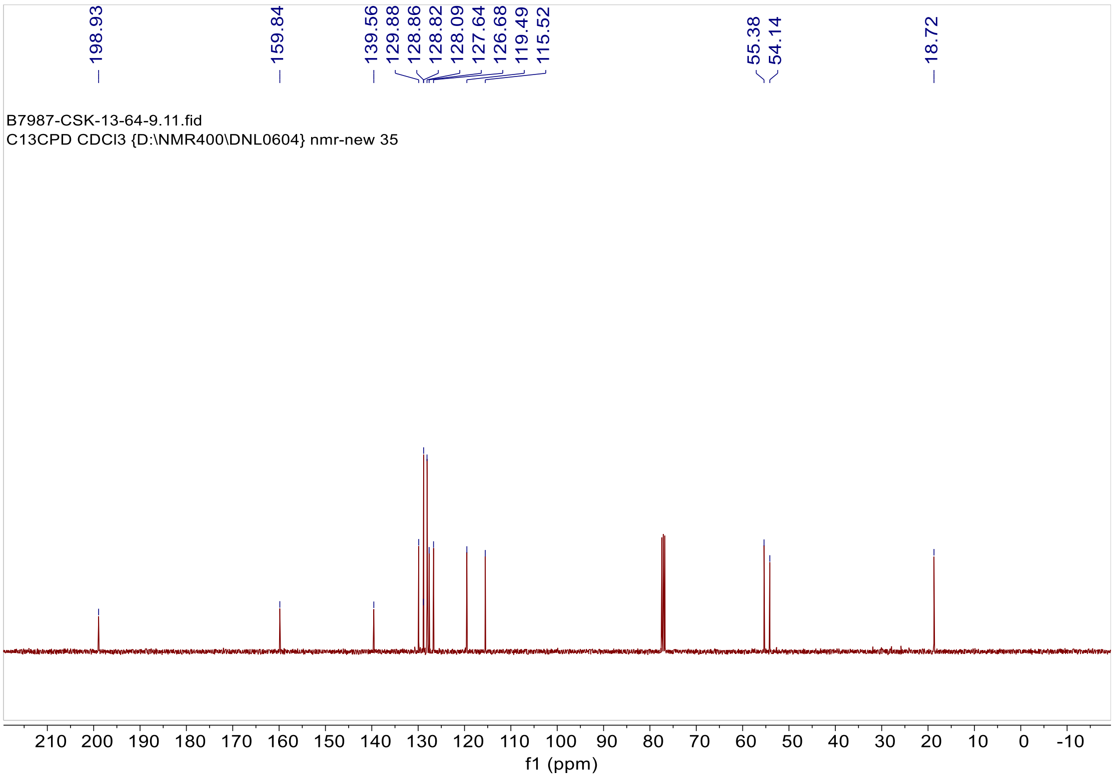

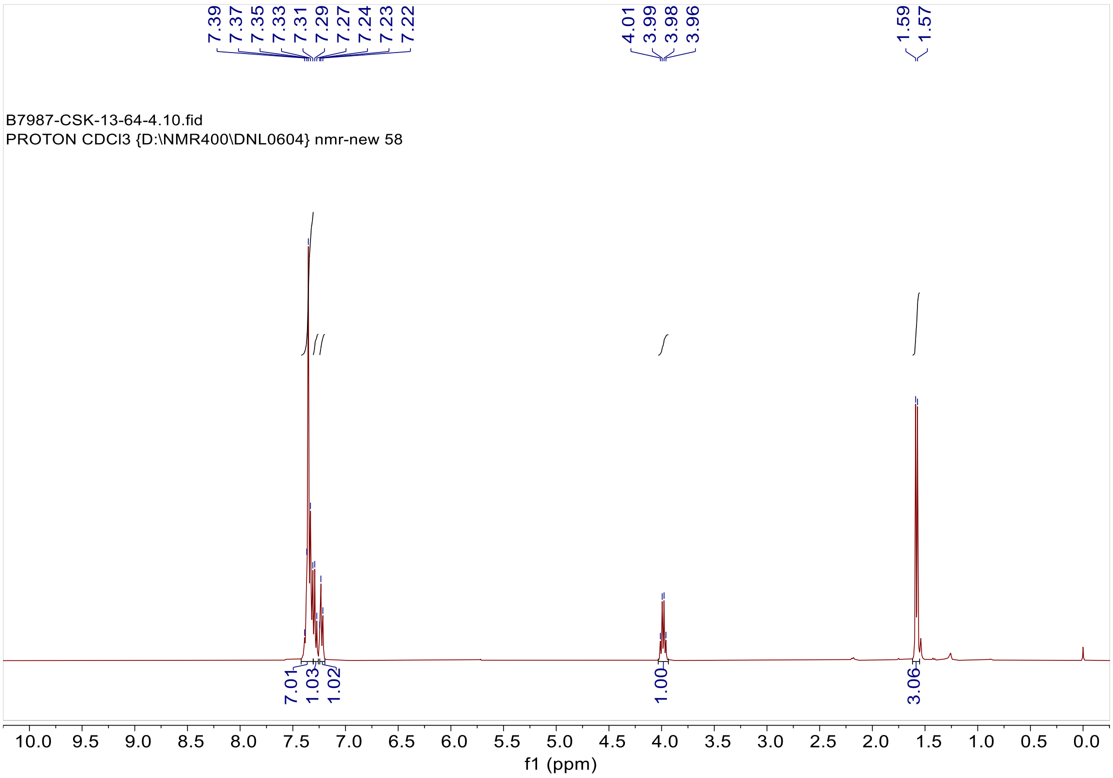

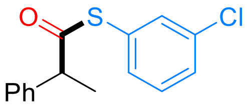

3ao

^{1}H NMR (400 MHz, $CDCl_3$)

^{13}C NMR (100 MHz, $CDCl_3$)

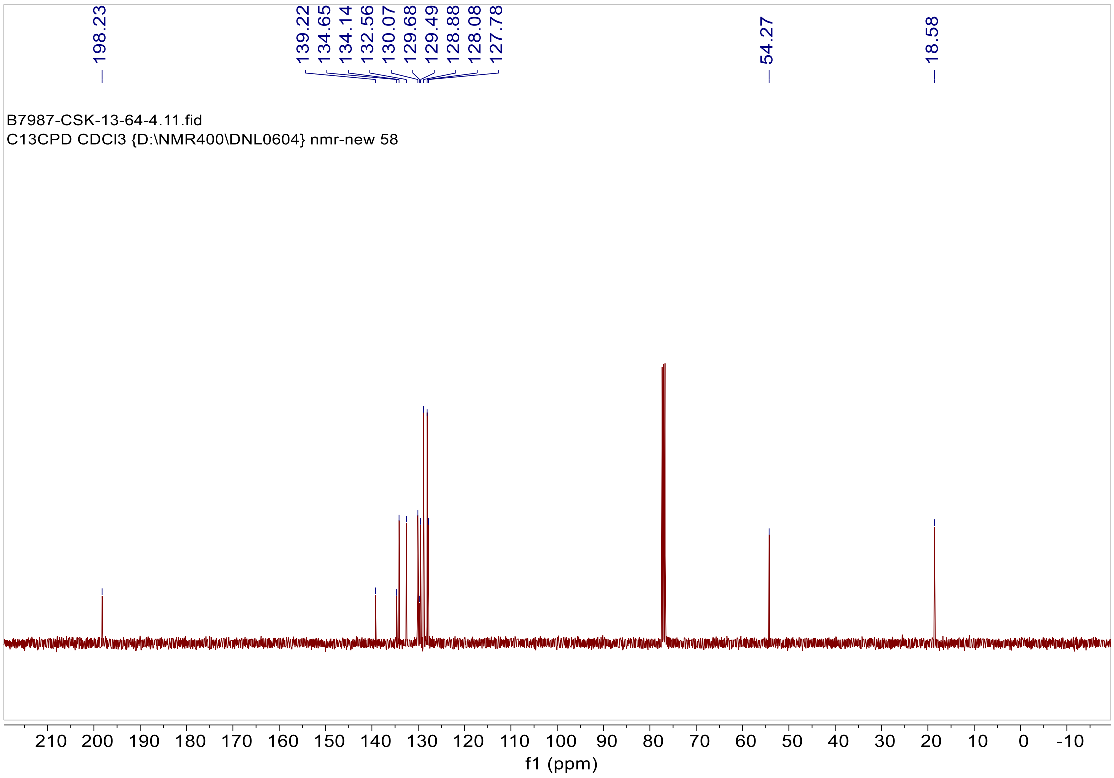

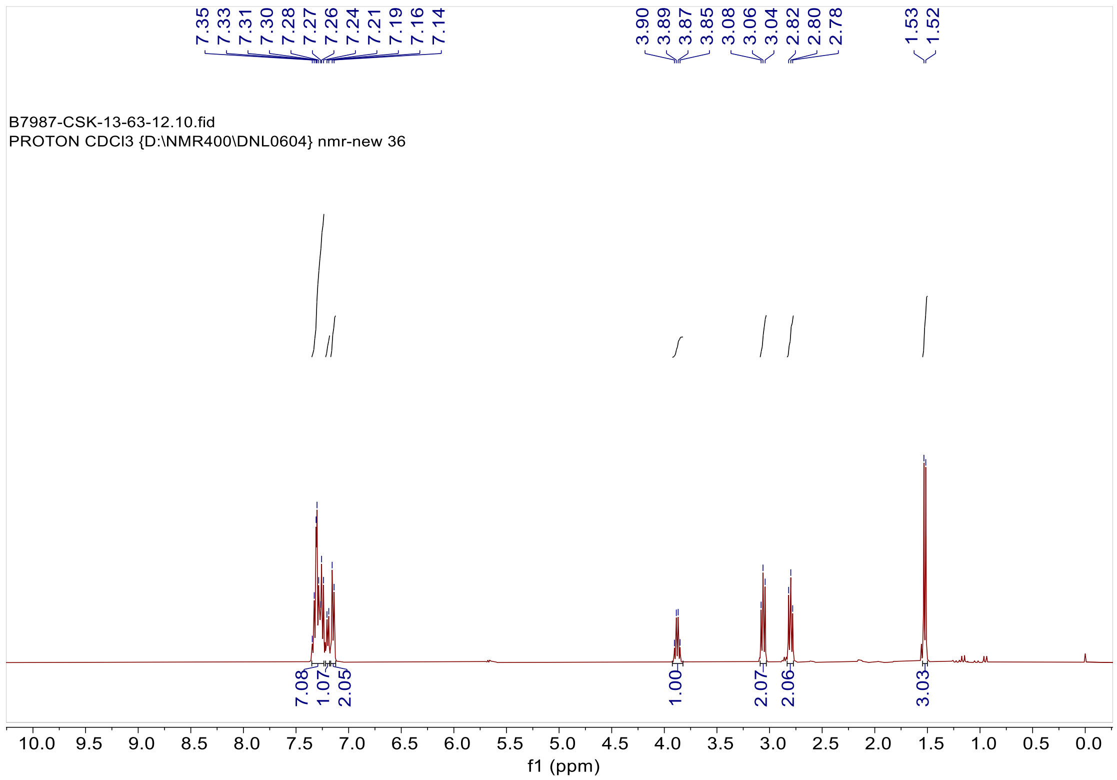

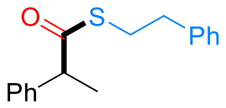

3ap

^{1}H NMR (400 MHz, $CDCl_3$)
^{13}C NMR (100 MHz, $CDCl_3$)

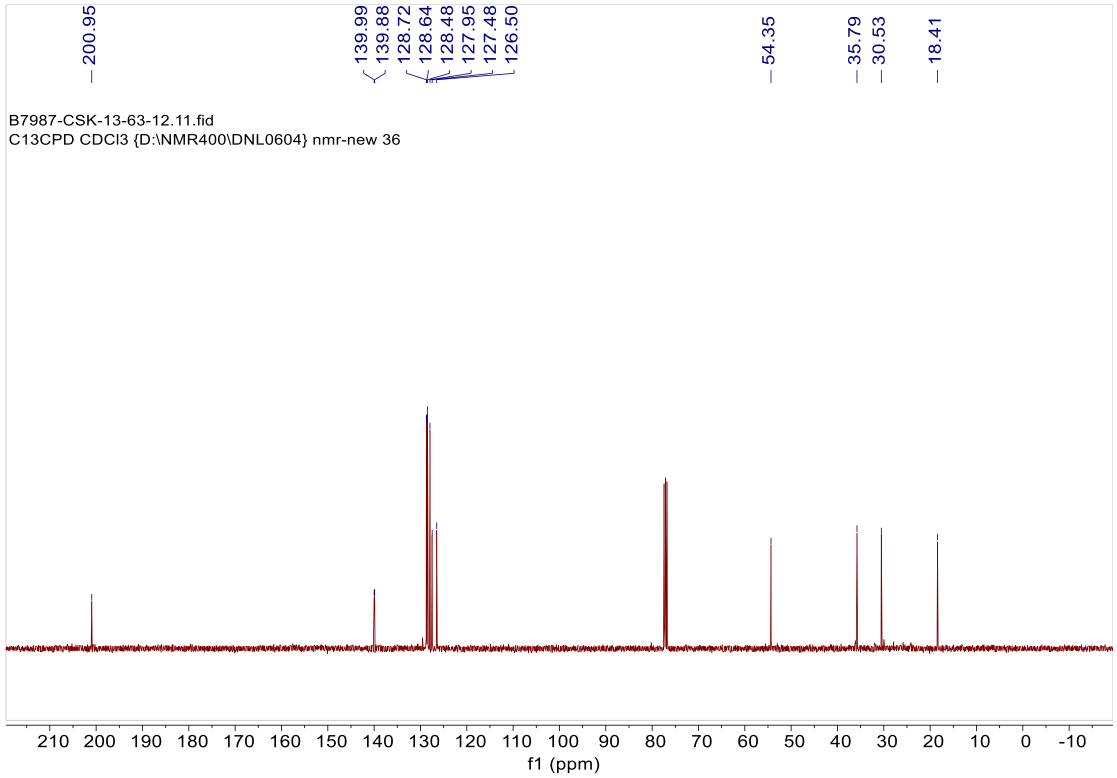

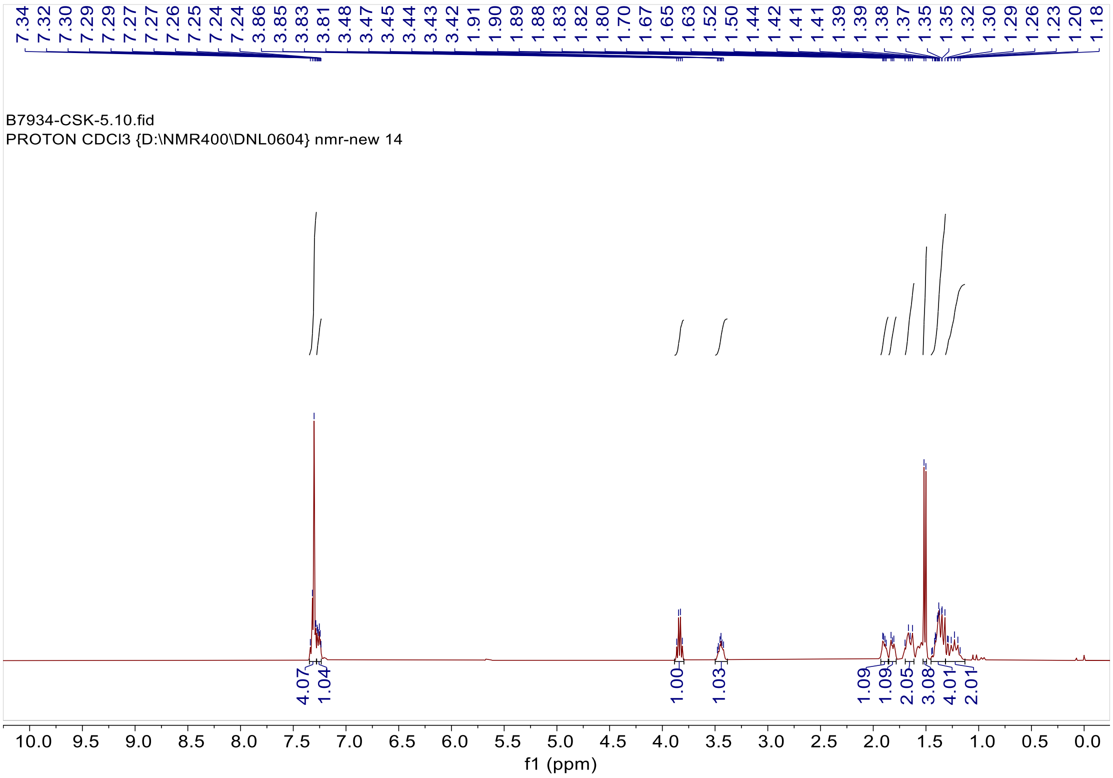

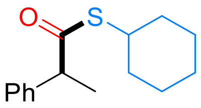

3aq

^{1}H NMR (400 MHz, $CDCl_3$)

^{13}C NMR (100 MHz, $CDCl_3$)

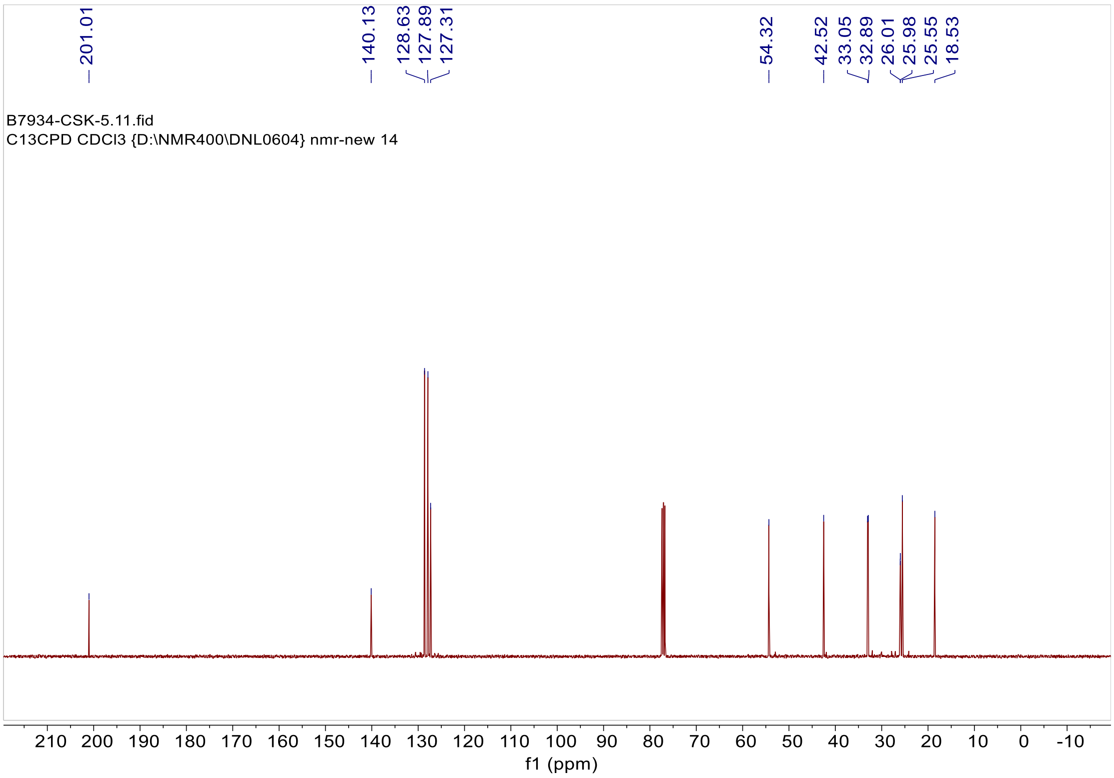

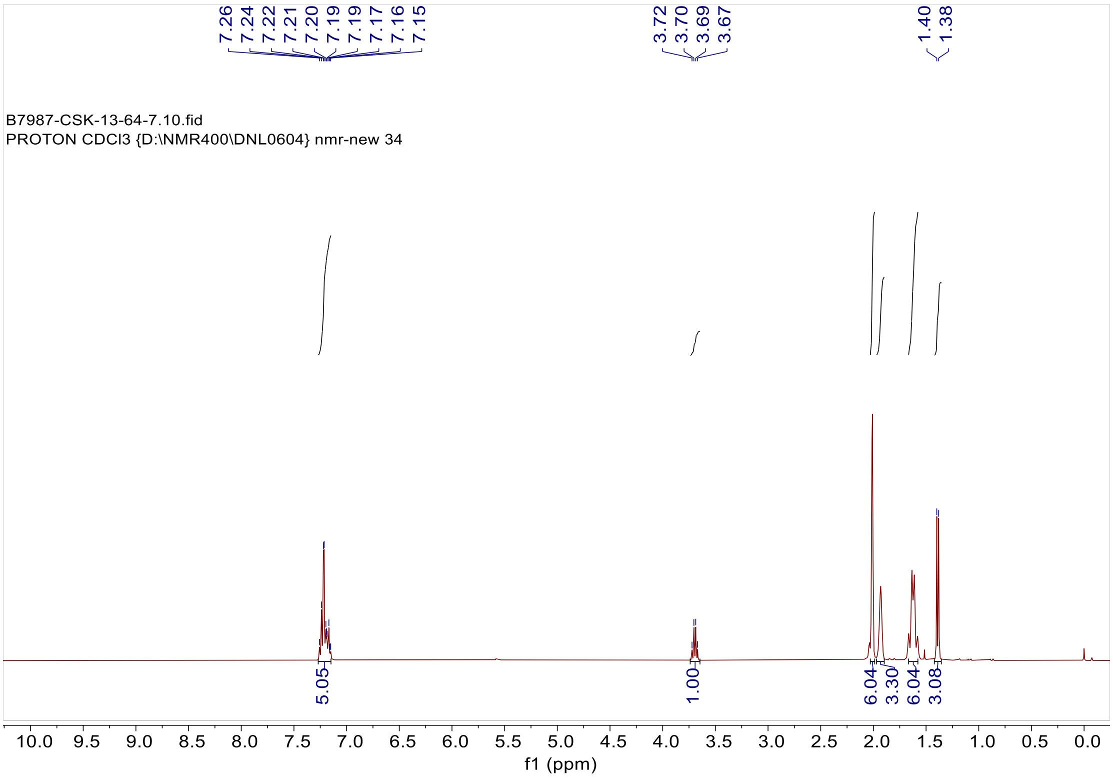

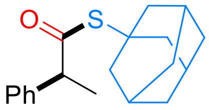

3ar

^{1}H NMR (400 MHz, $CDCl_3$)
^{13}C NMR (100 MHz, $CDCl_3$)

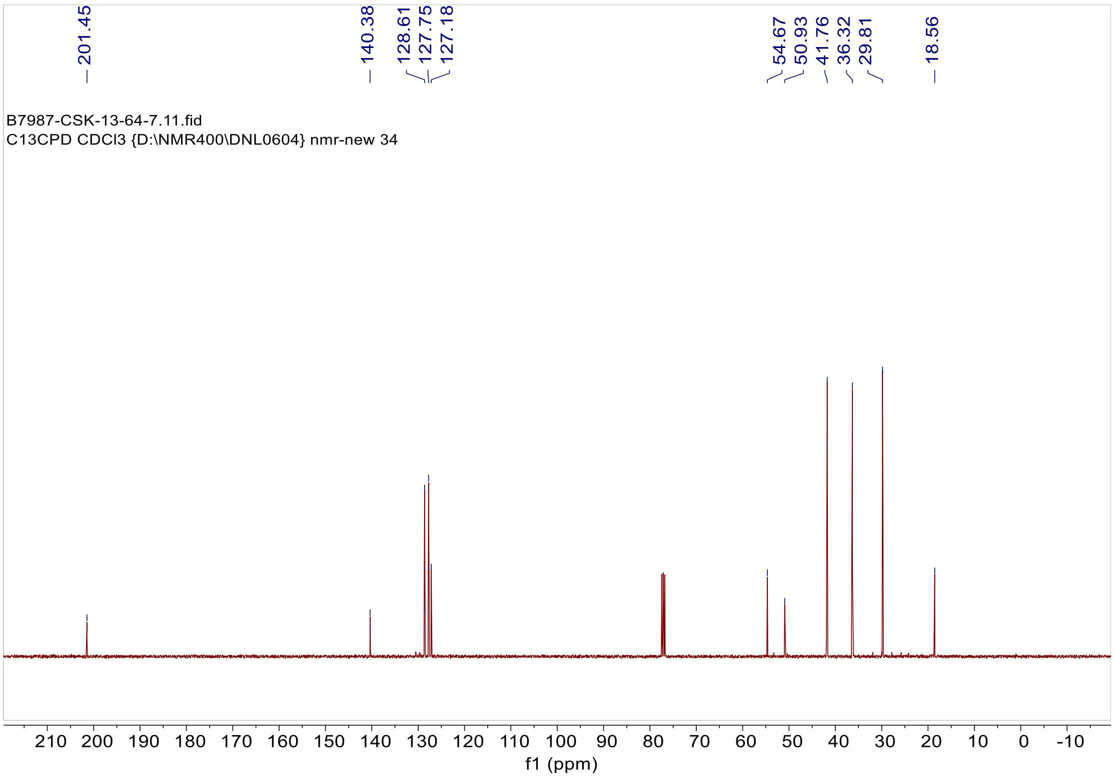

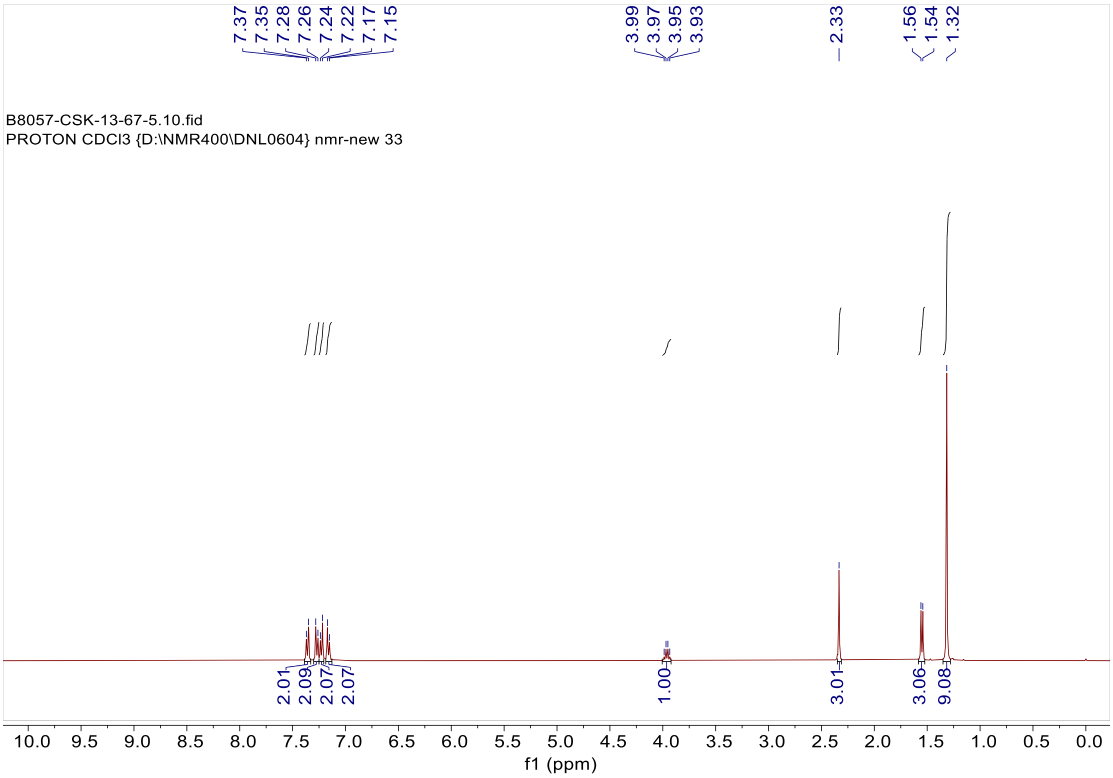

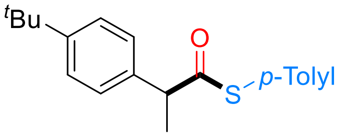

3ba

^{1}H NMR (400 MHz, $CDCl_3$)
^{13}C NMR (100 MHz, $CDCl_3$)

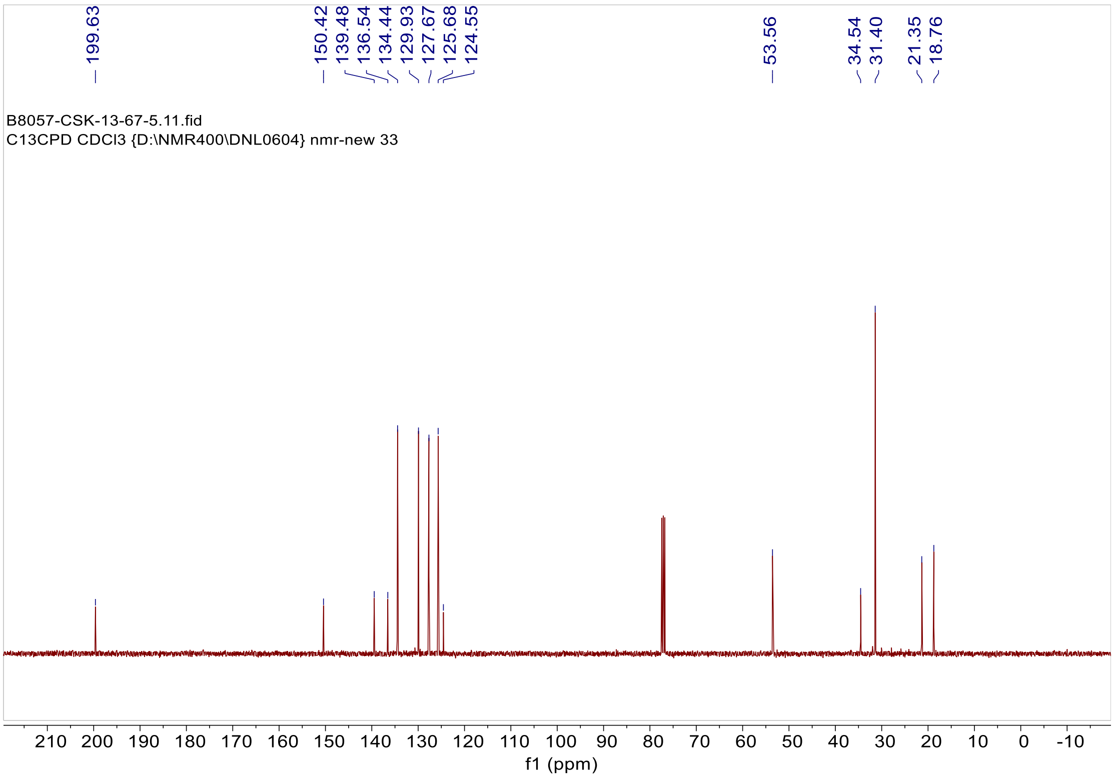

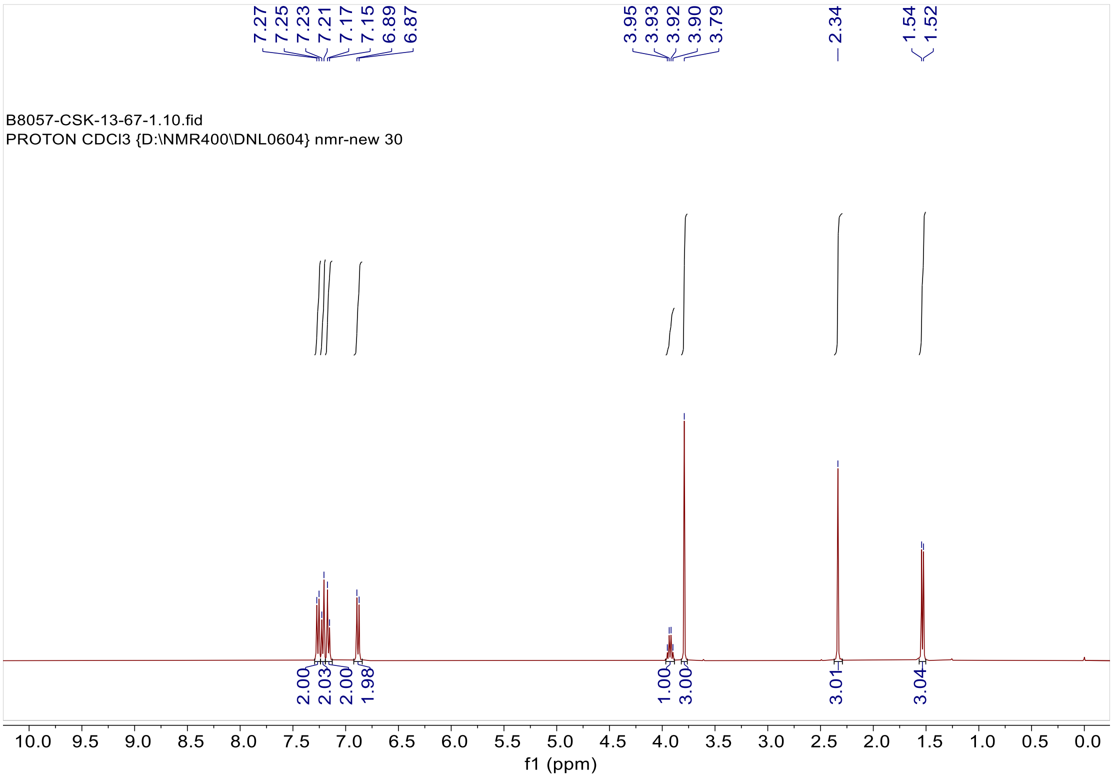

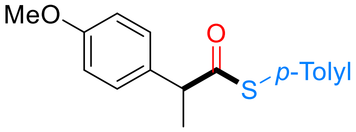

3ca

^{1}H NMR (400 MHz, $CDCl_3$)
^{13}C NMR (100 MHz, $CDCl_3$)

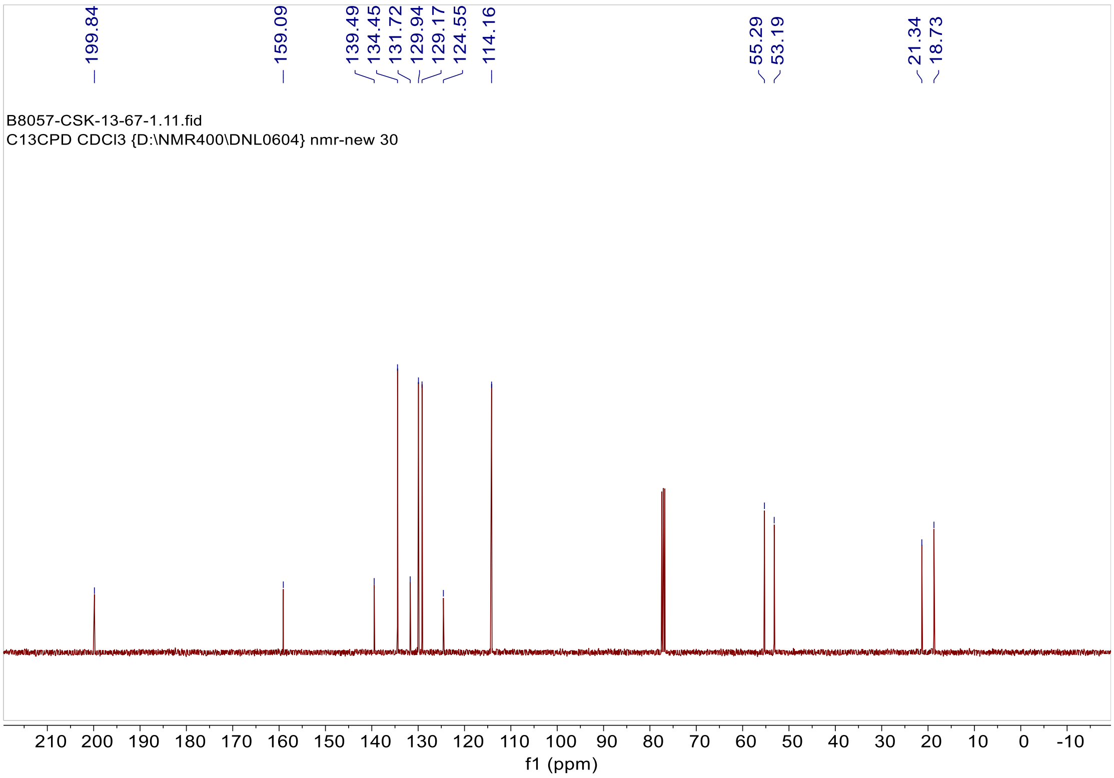

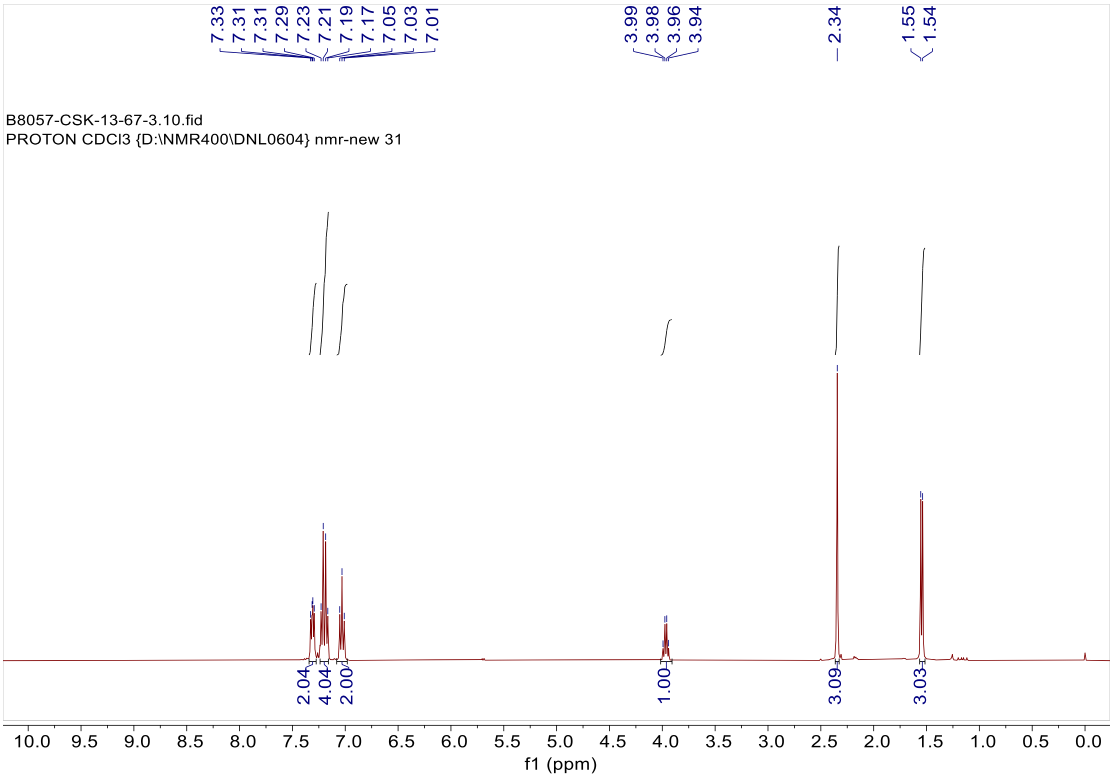

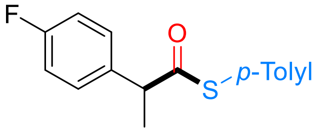

3da

^{1}H NMR (400 MHz, $CDCl_3$)
^{13}C NMR (100 MHz, $CDCl_3$)

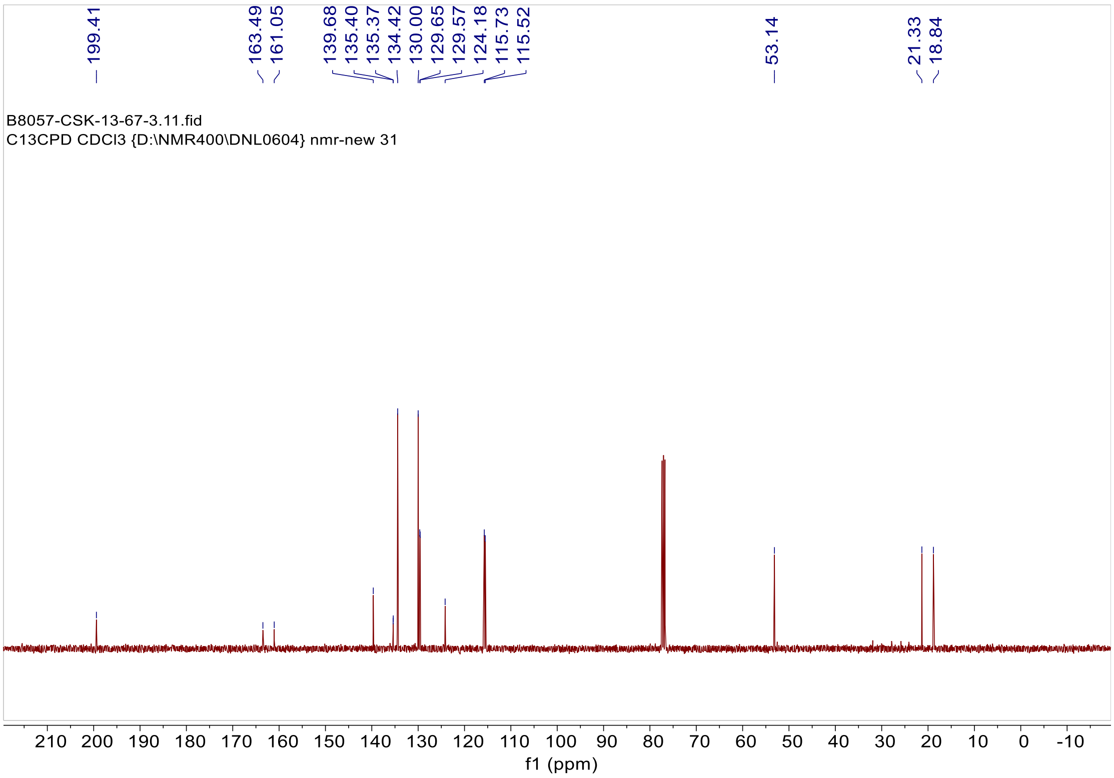

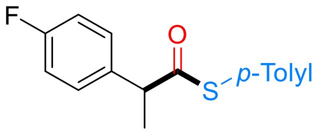

3da

^{19}F NMR (376 MHz, $CDCl_3$)

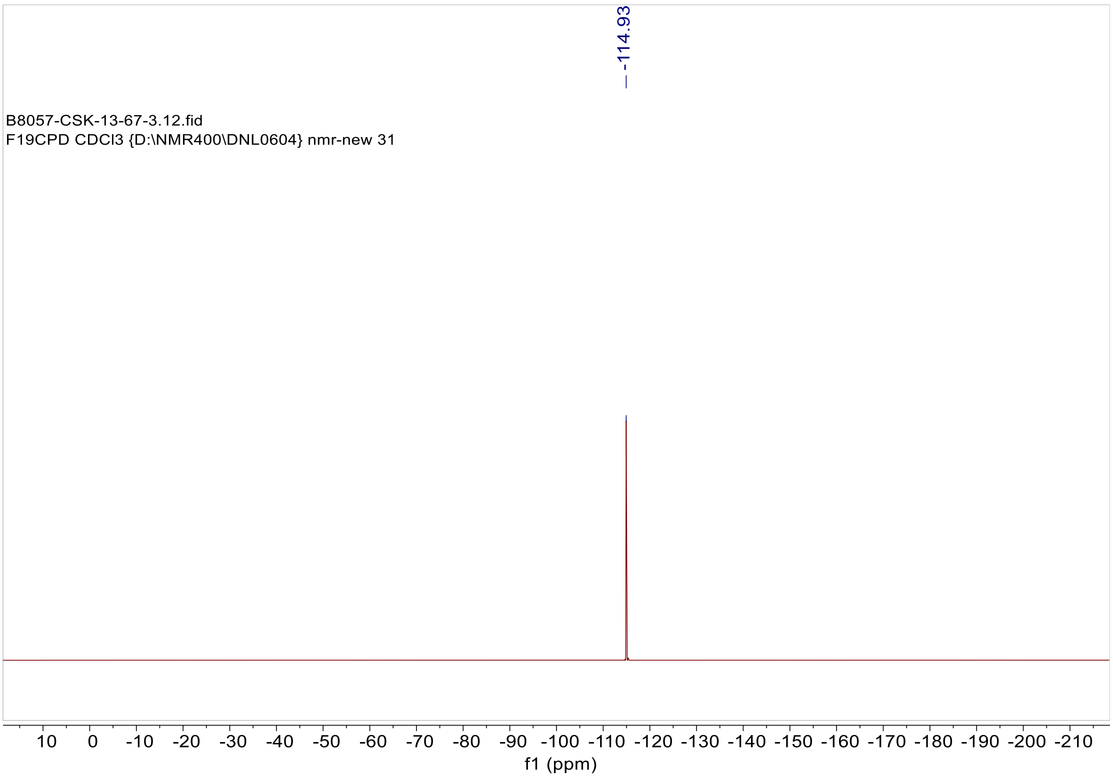

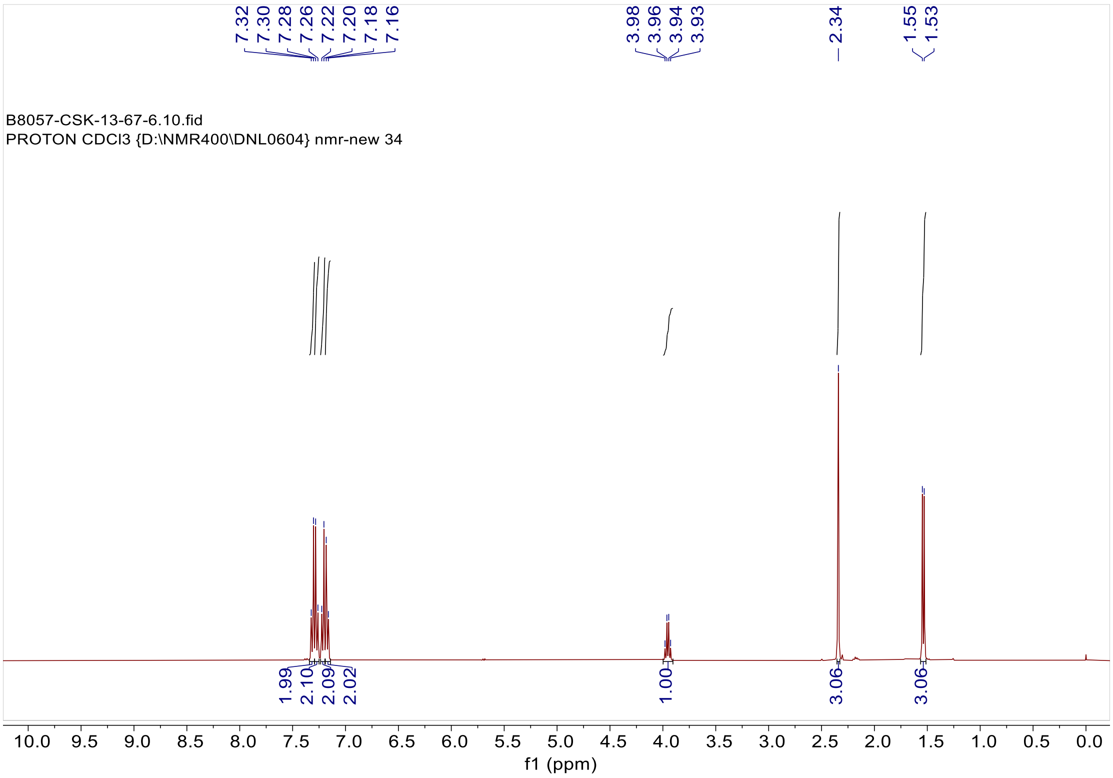

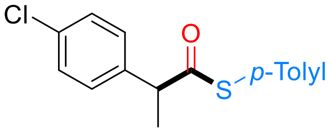

3ea

^{1}H NMR (400 MHz, $CDCl_3$)

^{13}C NMR (100 MHz, $CDCl_3$)

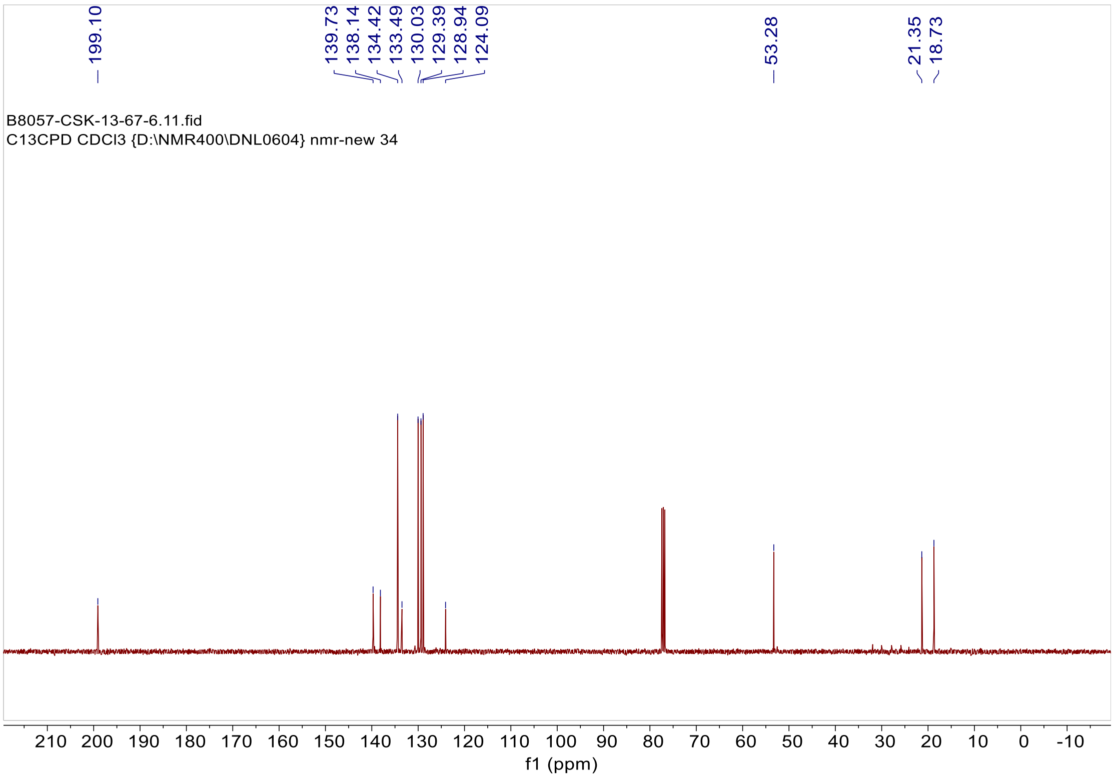

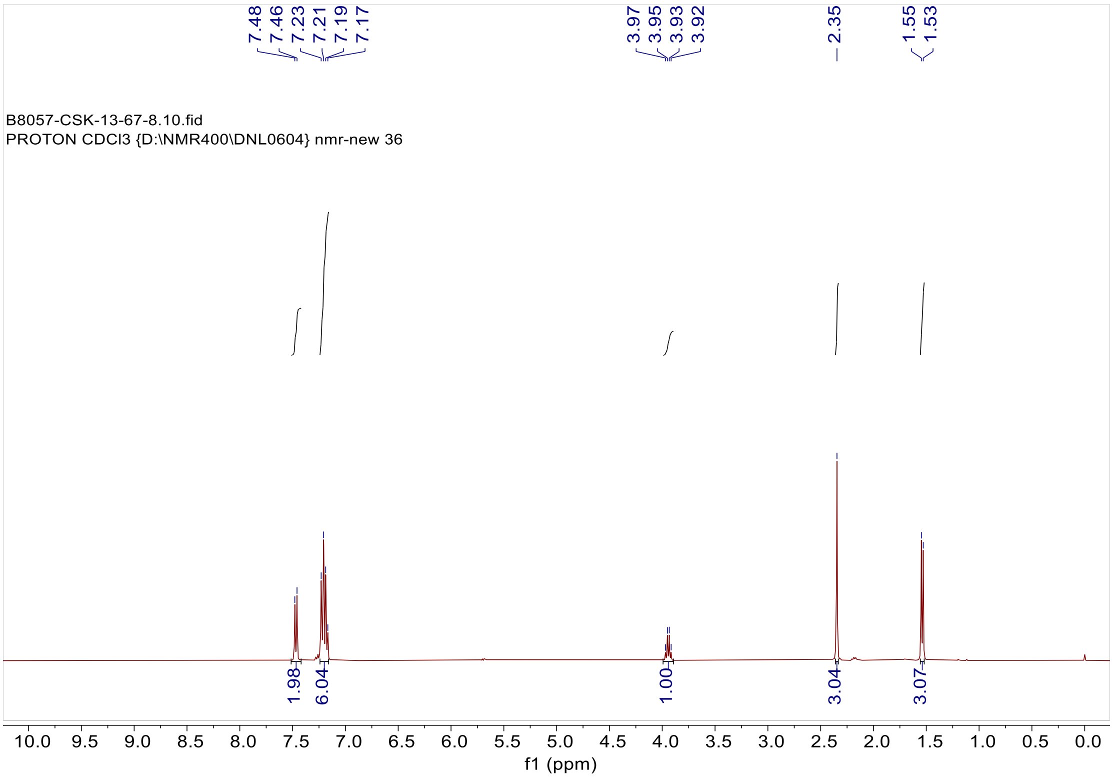

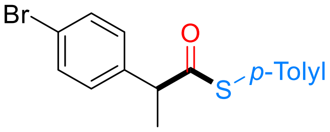

3fa

^{1}H NMR (400 MHz, $CDCl_3$)
^{13}C NMR (100 MHz, $CDCl_3$)

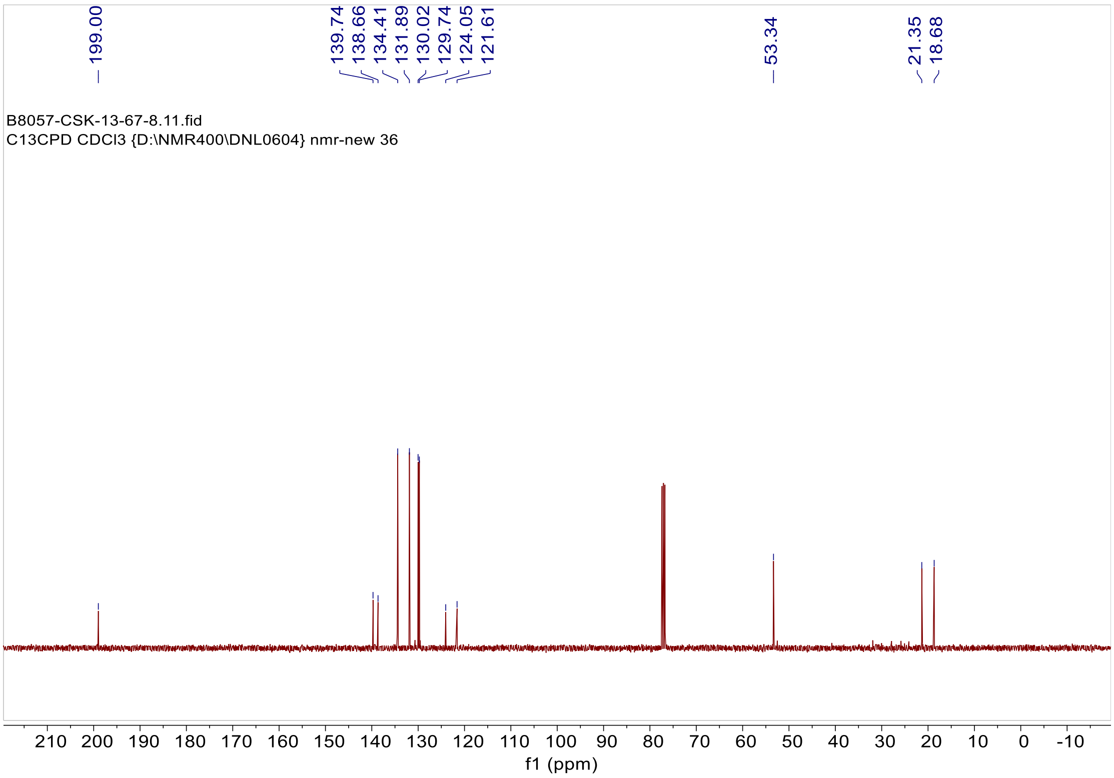

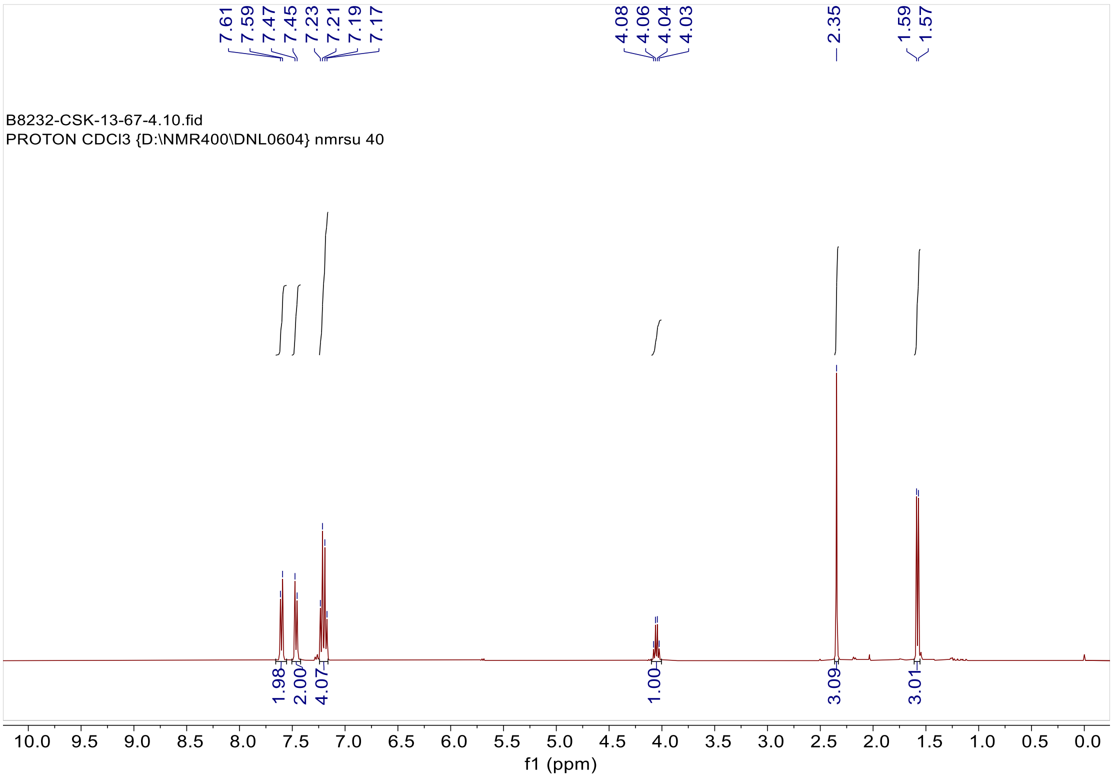

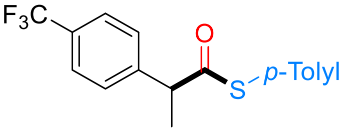

3ga

^{1}H NMR (400 MHz, $CDCl_3$)

^{13}C NMR (100 MHz, $CDCl_3$)

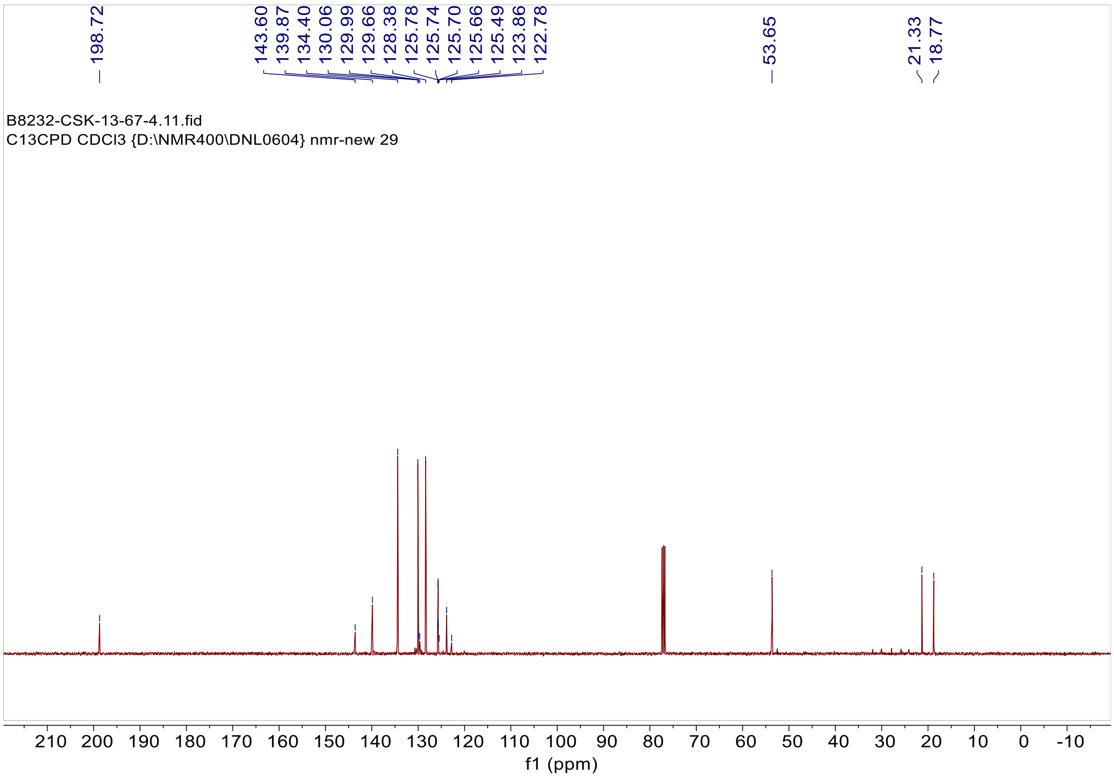

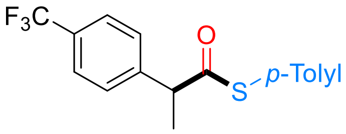

3ga

^{19}F NMR (376 MHz, $CDCl_3$)

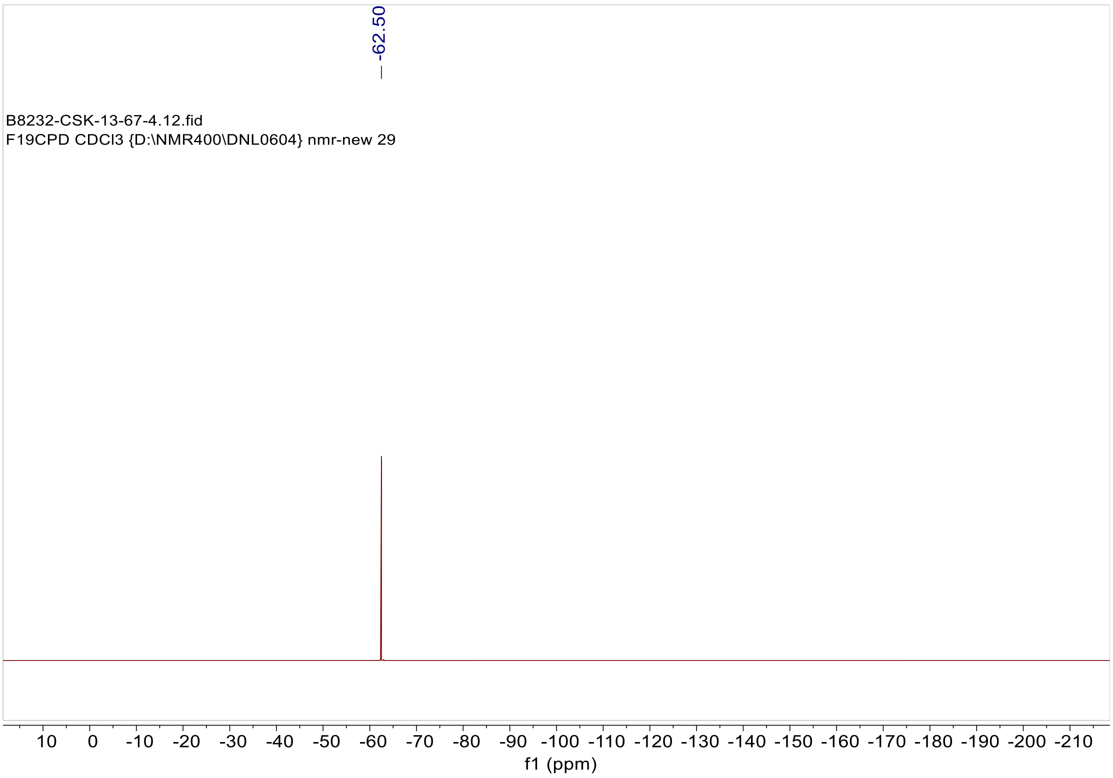

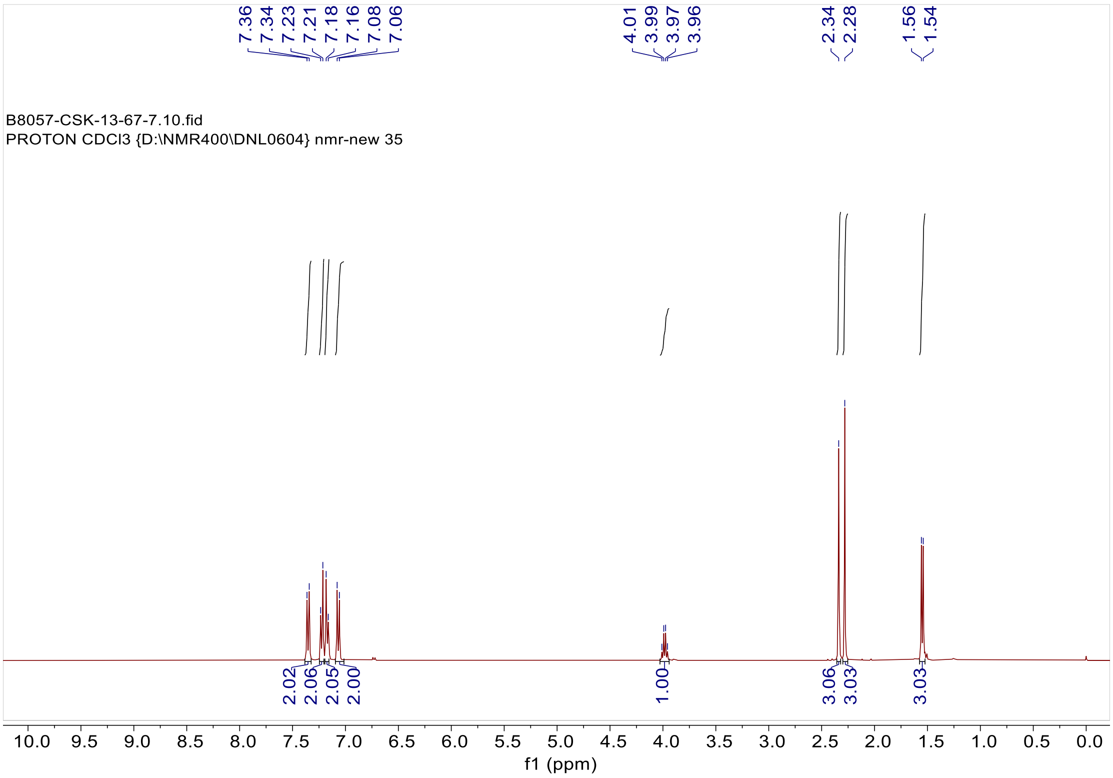

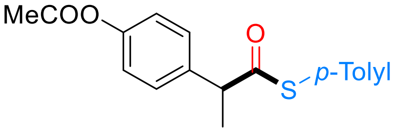

3ha

^{1}H NMR (400 MHz, $CDCl_3$)
^{13}C NMR (100 MHz, $CDCl_3$)

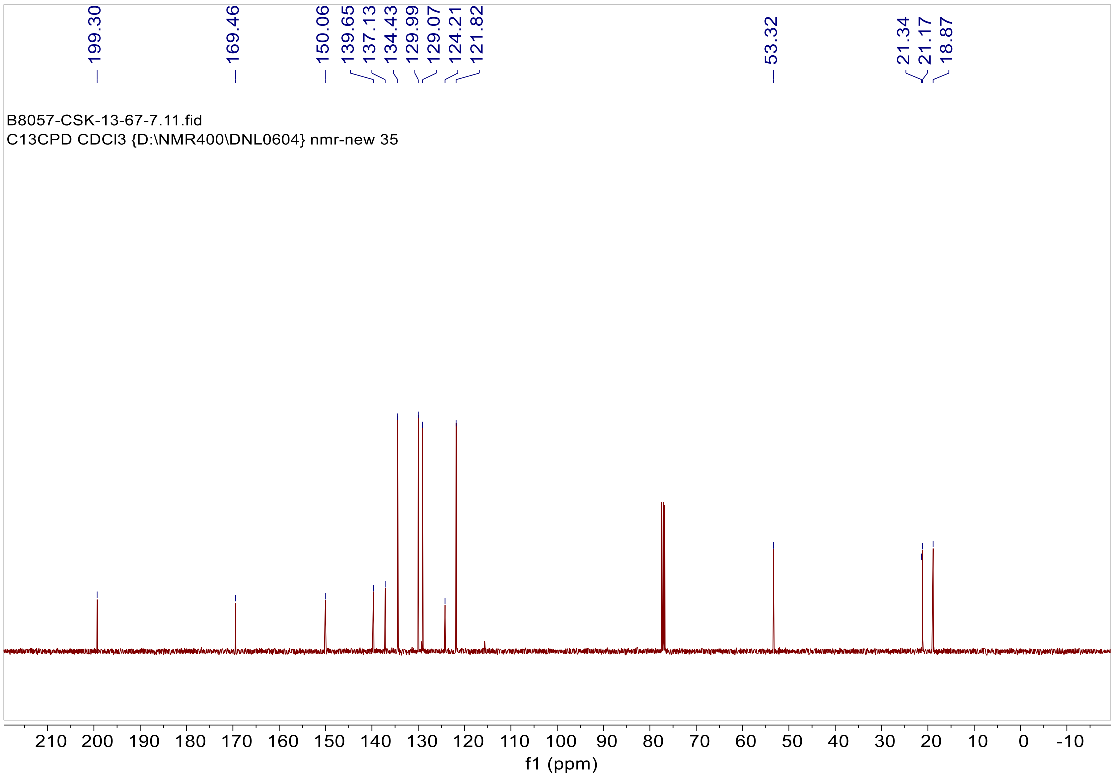

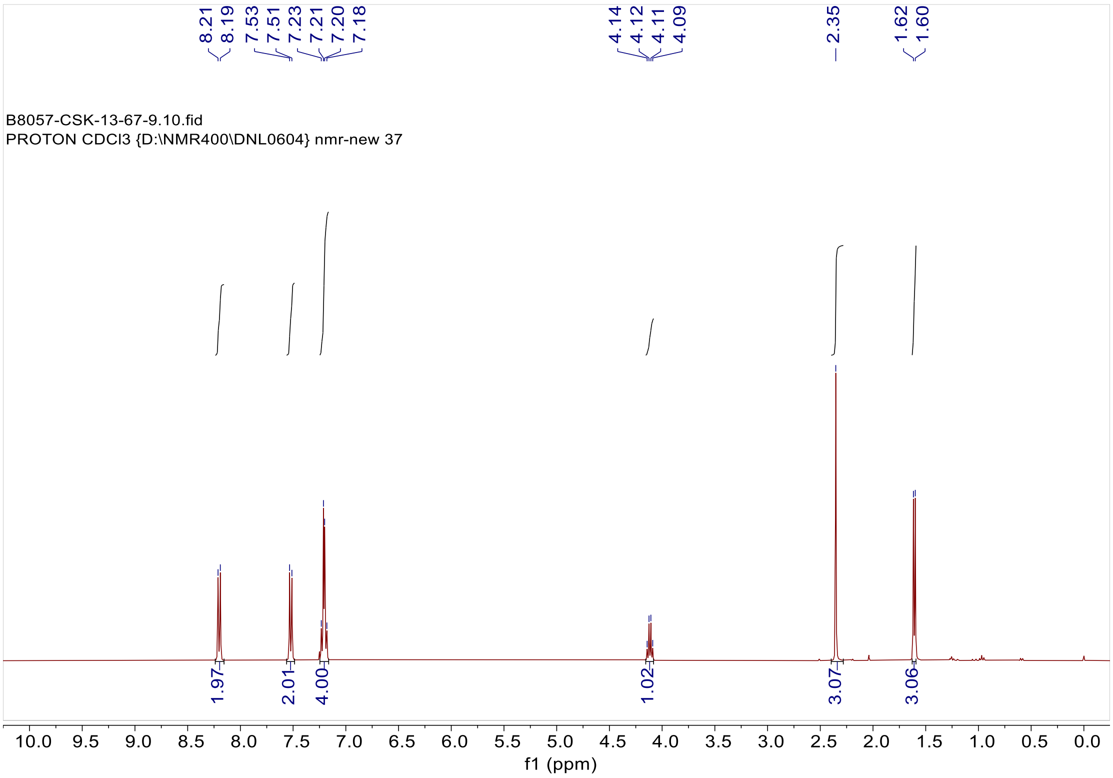

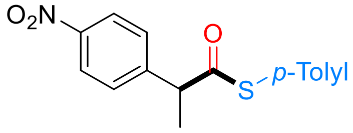

3ia

^{1}H NMR (400 MHz, $CDCl_3$)
^{13}C NMR (100 MHz, $CDCl_3$)

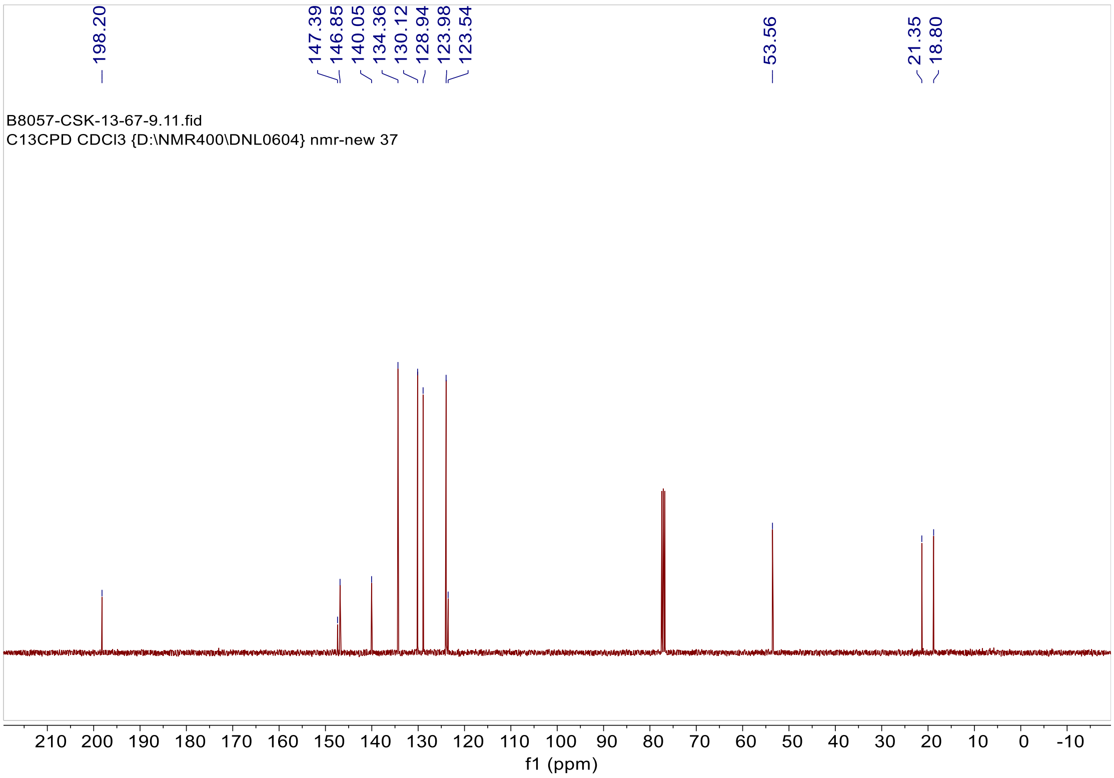

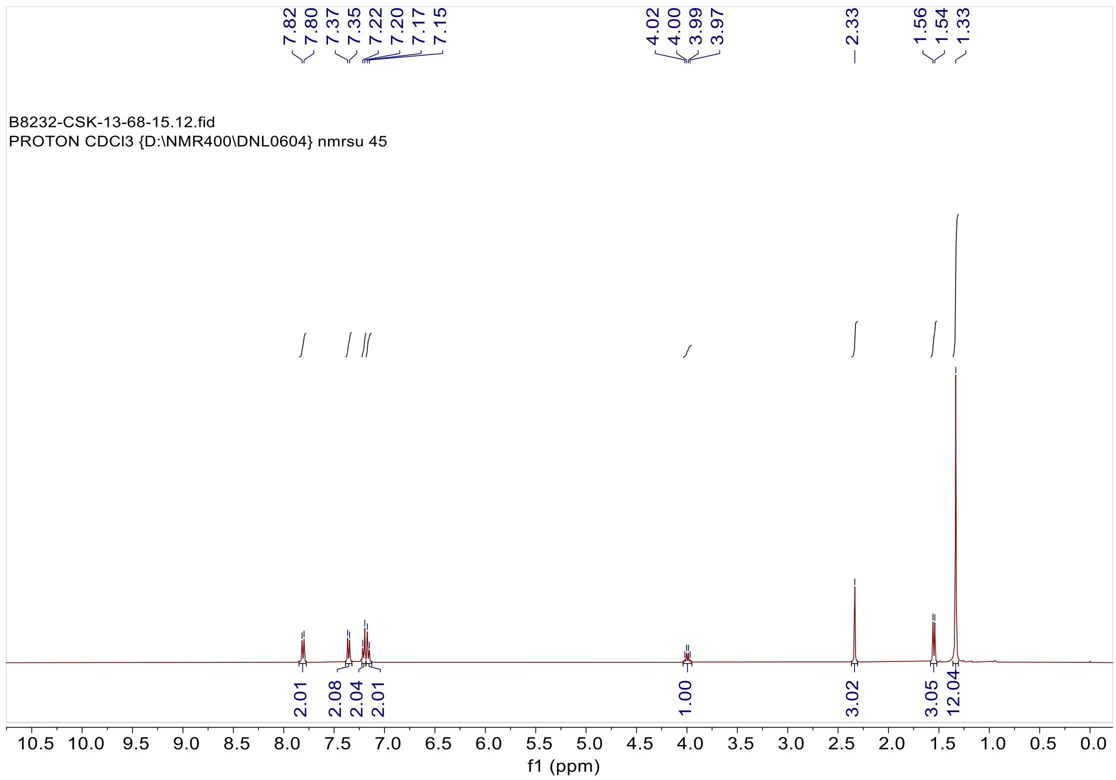

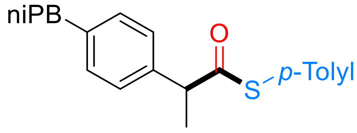

3ja

^{1}H NMR (400 MHz, $CDCl_3$)

^{13}C NMR (100 MHz, $CDCl_3$)

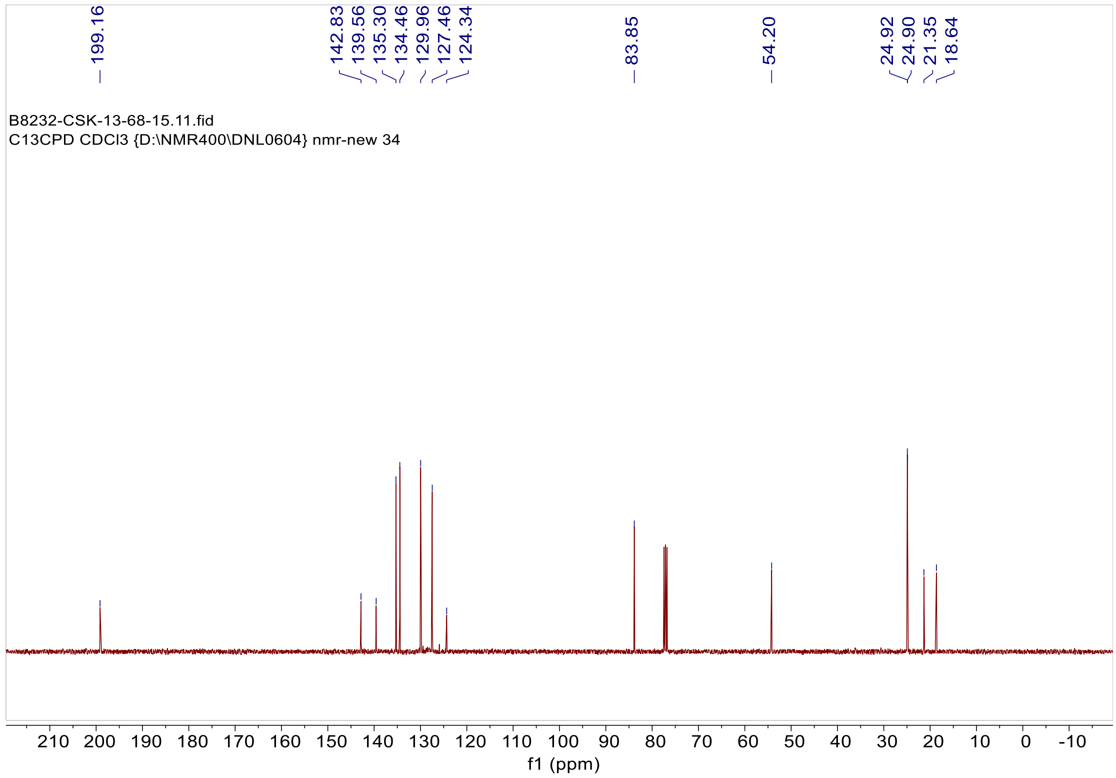

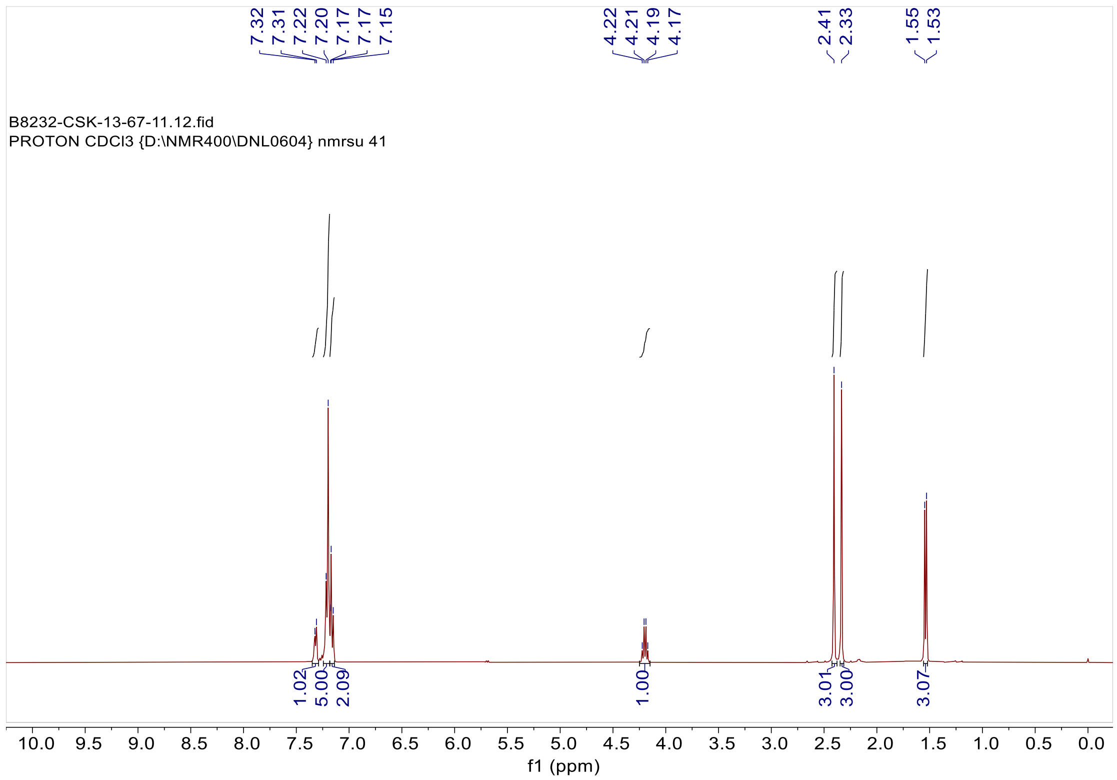

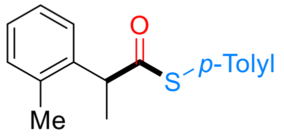

3ka

^{1}H NMR (400 MHz, $CDCl_3$)

^{13}C NMR (100 MHz, $CDCl_3$)

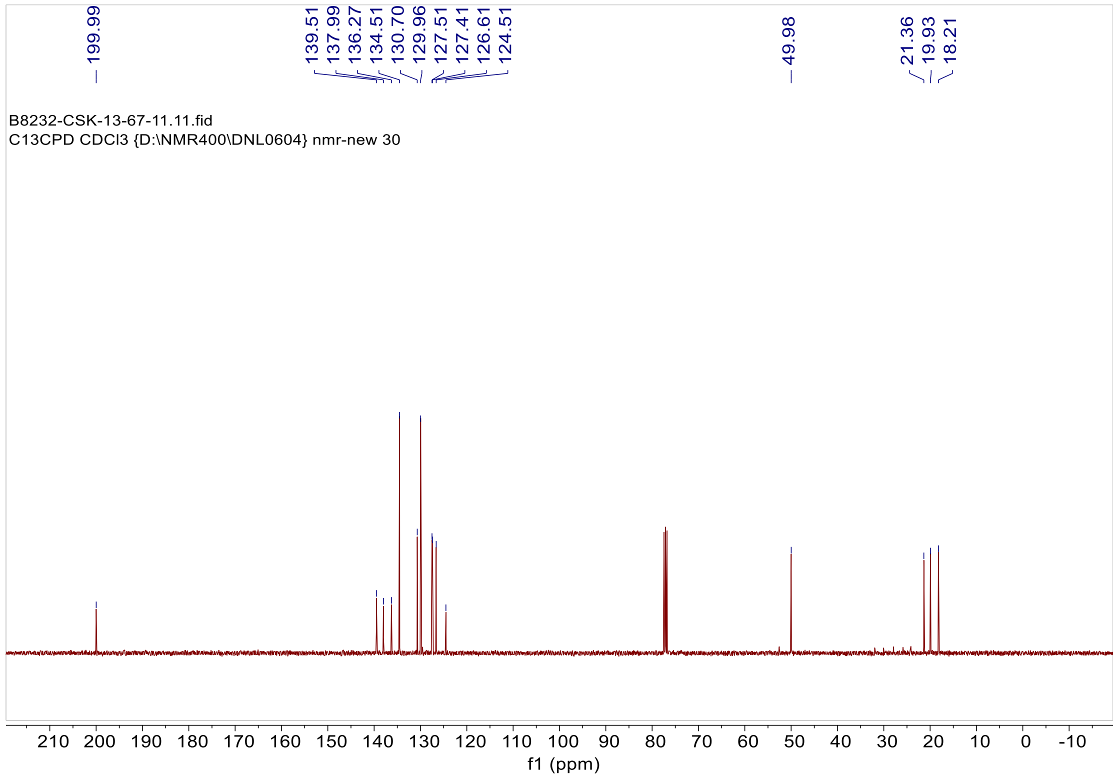

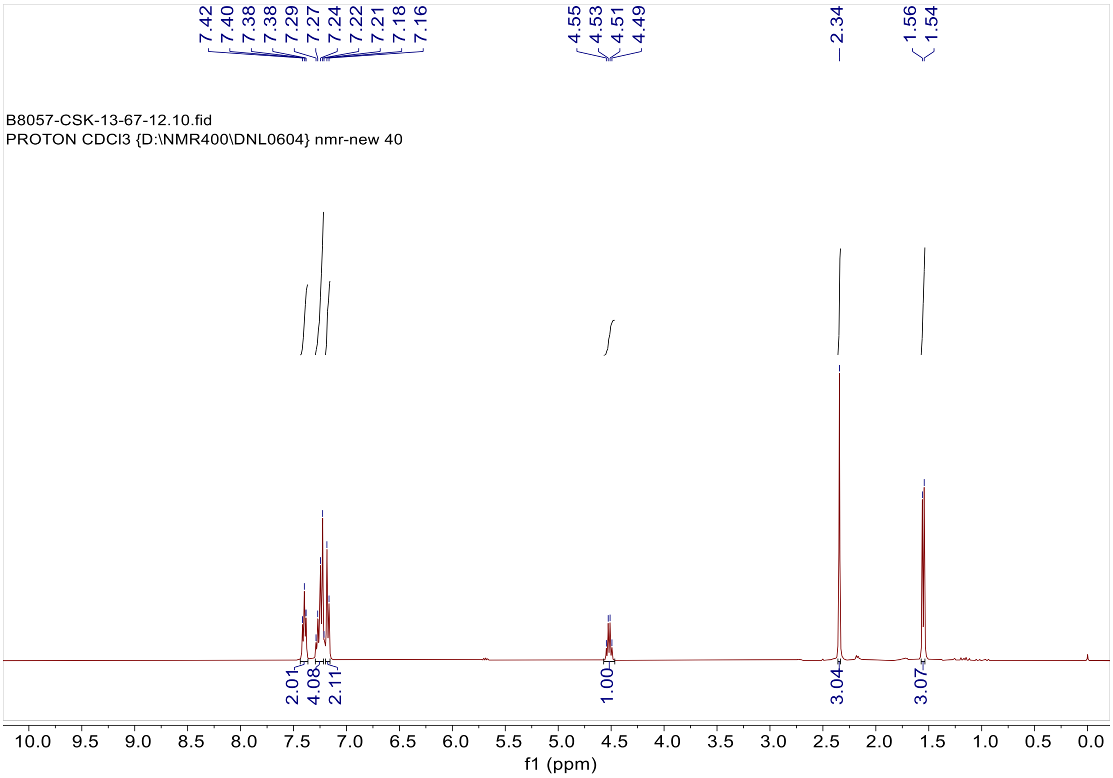

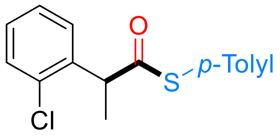

3la

^{1}H NMR (400 MHz, $CDCl_3$)

^{13}C NMR (100 MHz, $CDCl_3$)

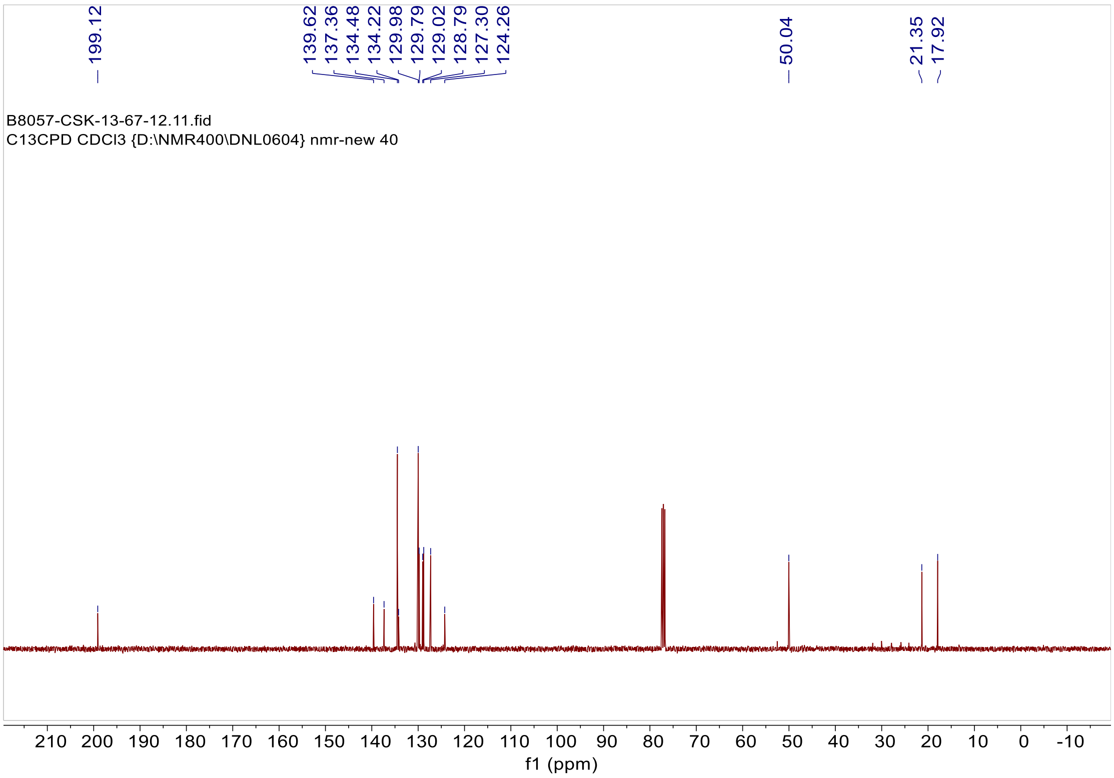

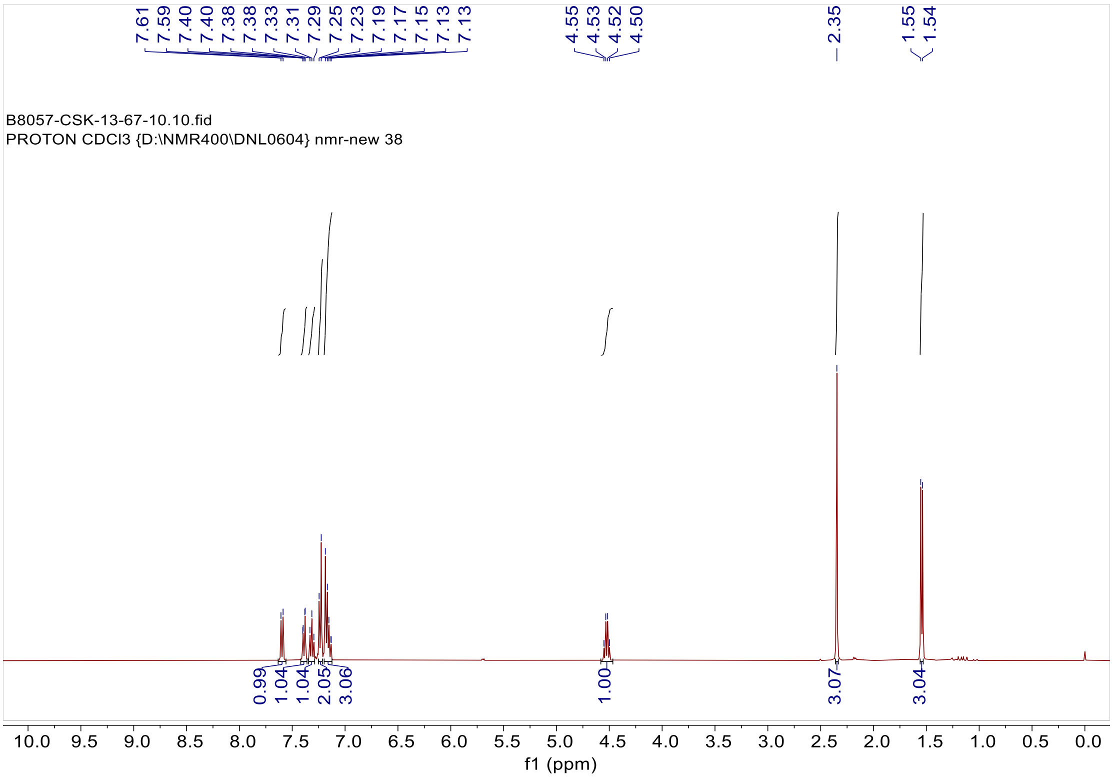

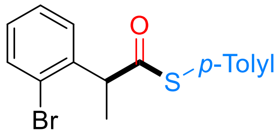

3ma

^{1}H NMR (400 MHz, $CDCl_3$)
^{13}C NMR (100 MHz, $CDCl_3$)

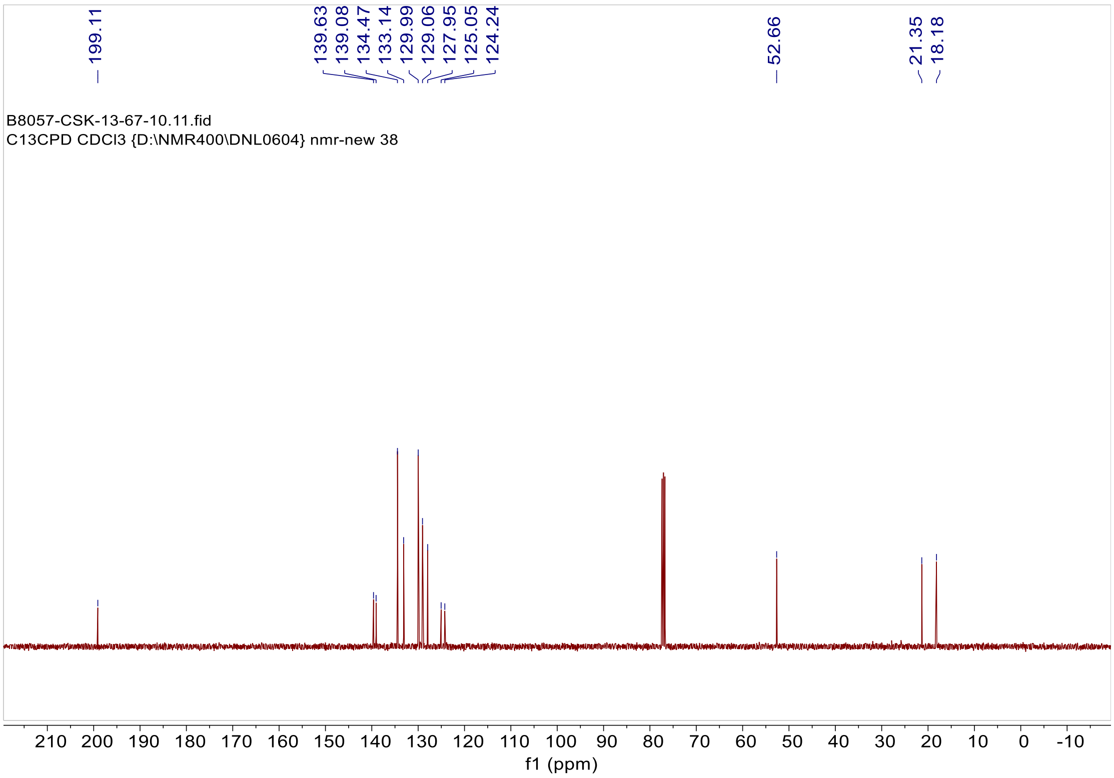

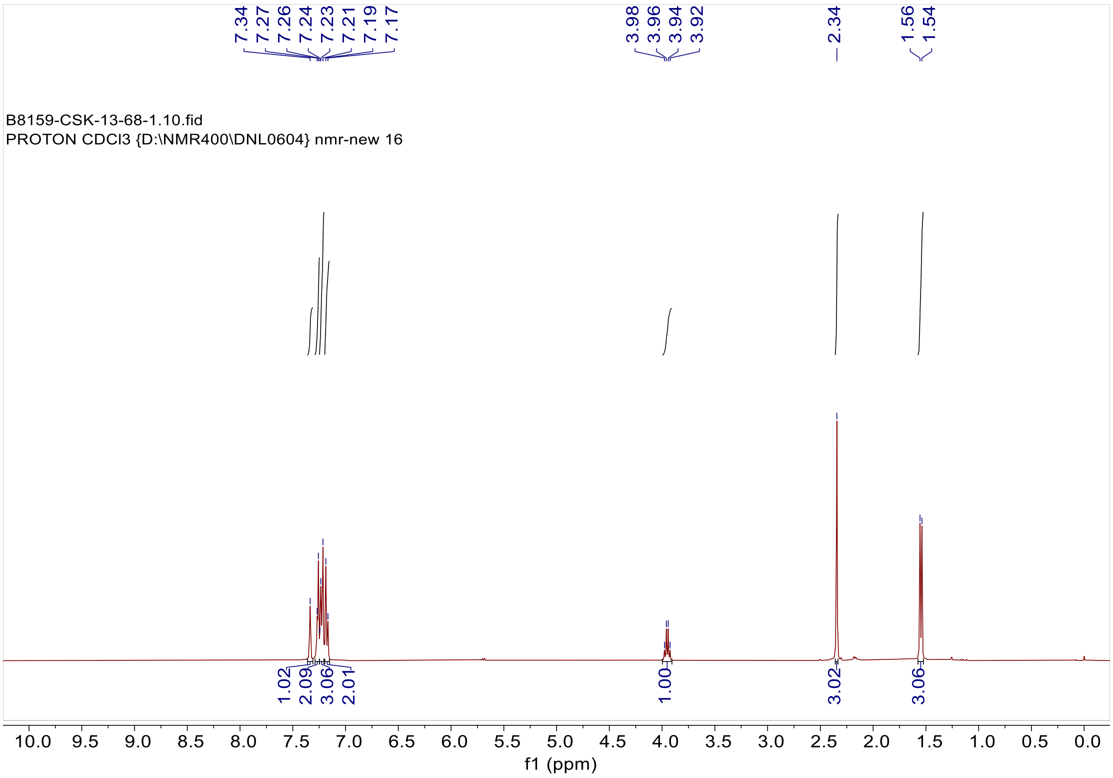

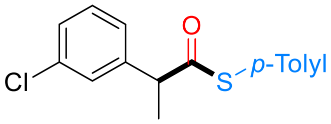

3na

^{1}H NMR (400 MHz, $CDCl_3$)
^{13}C NMR (100 MHz, $CDCl_3$)

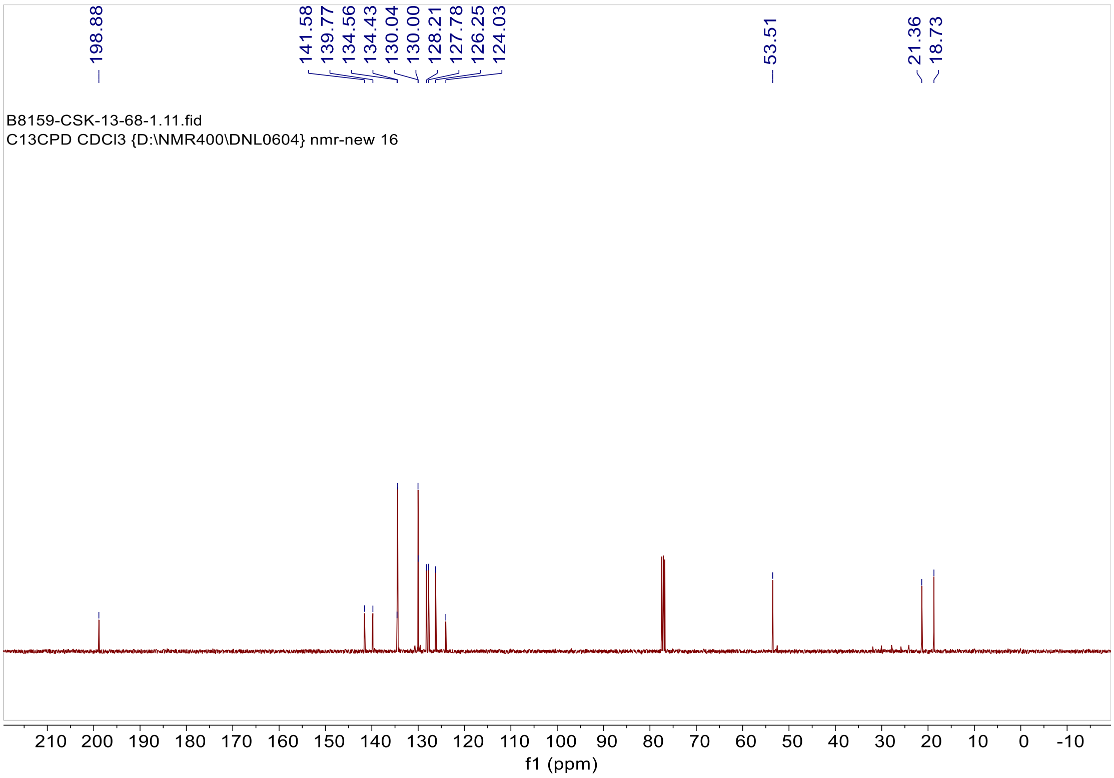

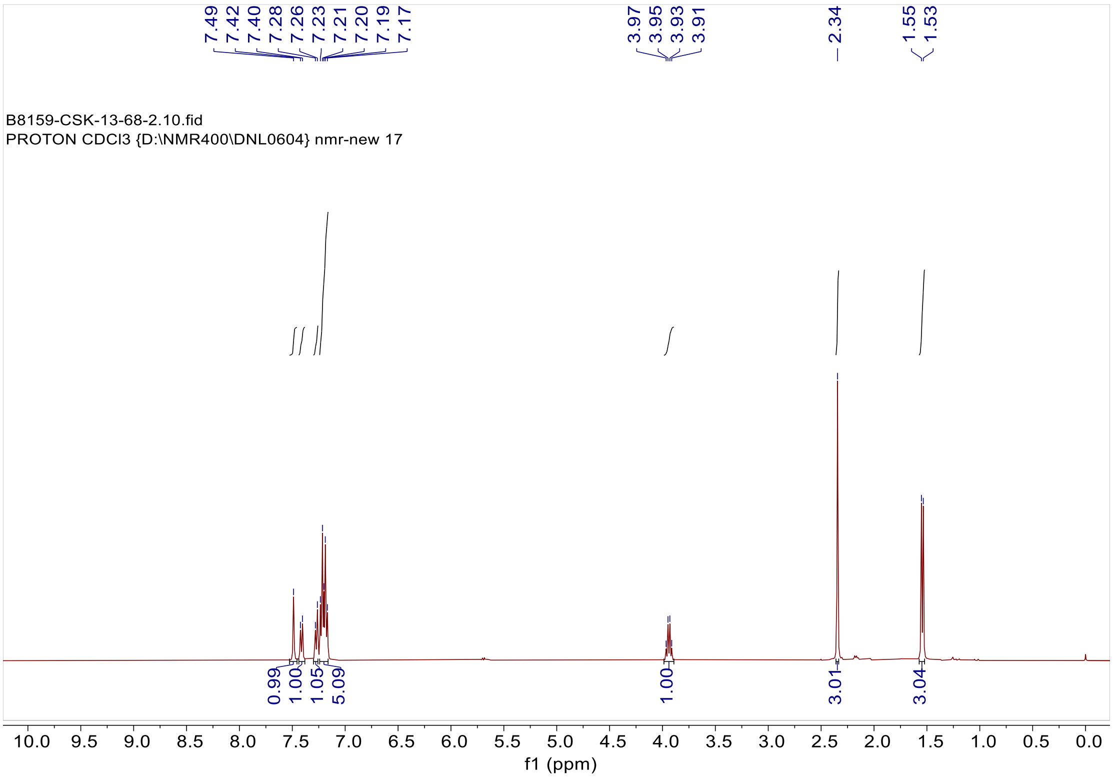

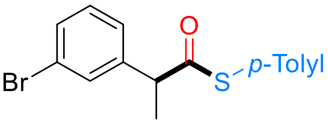

3oa

^{1}H NMR (400 MHz, $CDCl_3$)

^{13}C NMR (100 MHz, $CDCl_3$)

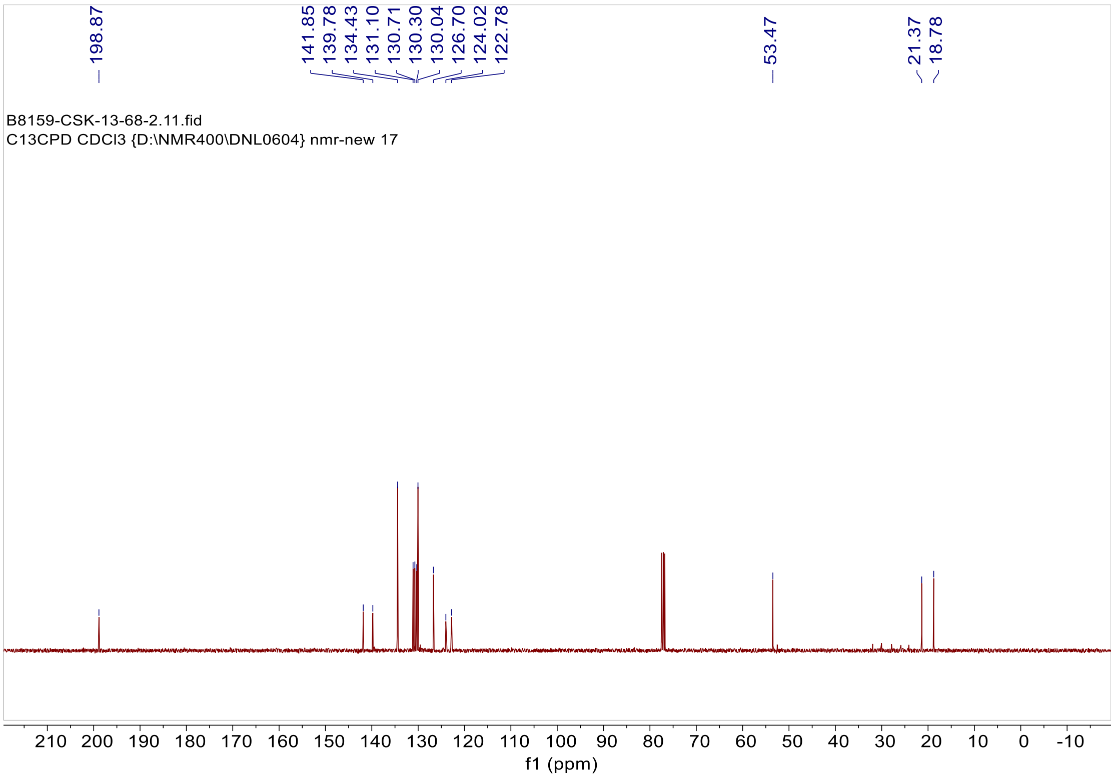

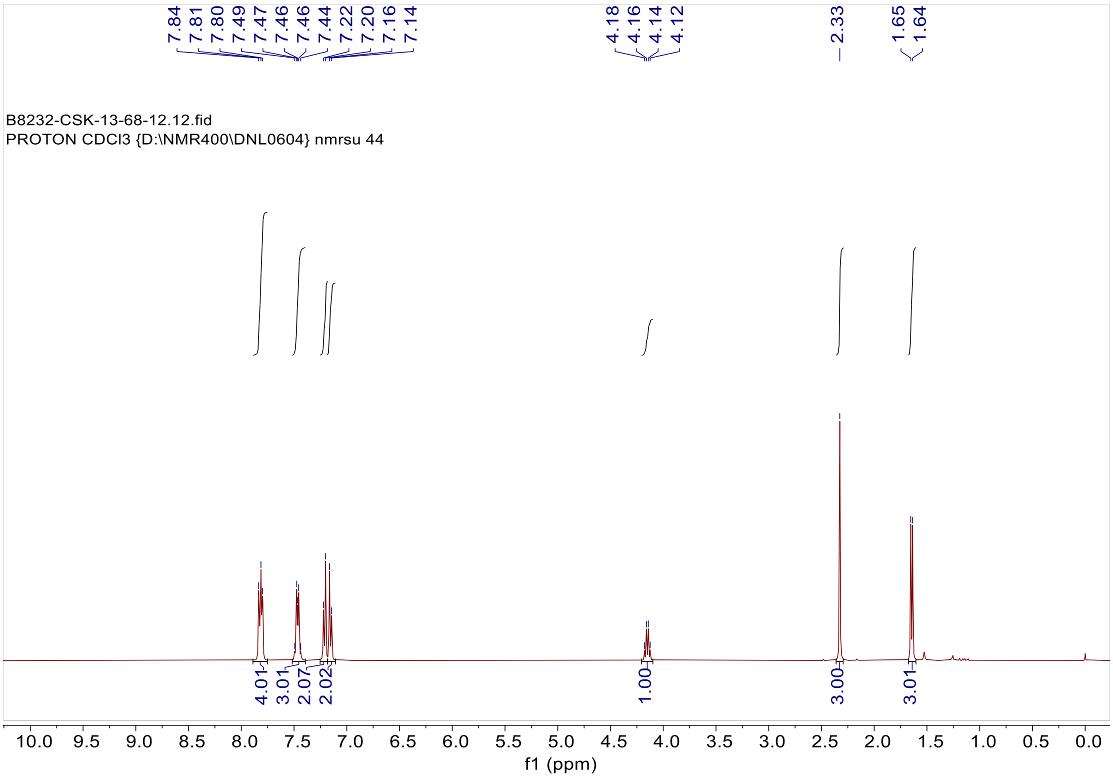

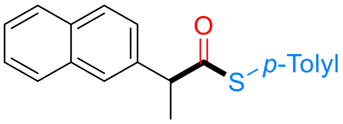

3pa

^{1}H NMR (400 MHz, $CDCl_3$)

^{13}C NMR (100 MHz, $CDCl_3$)

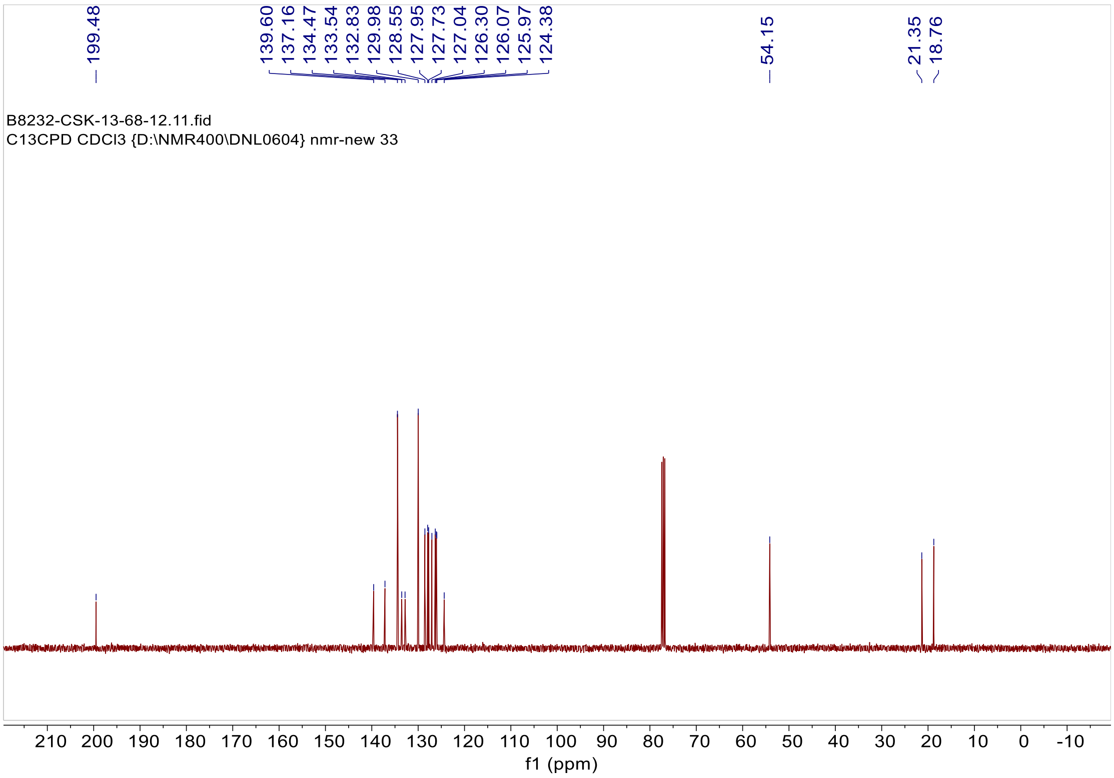

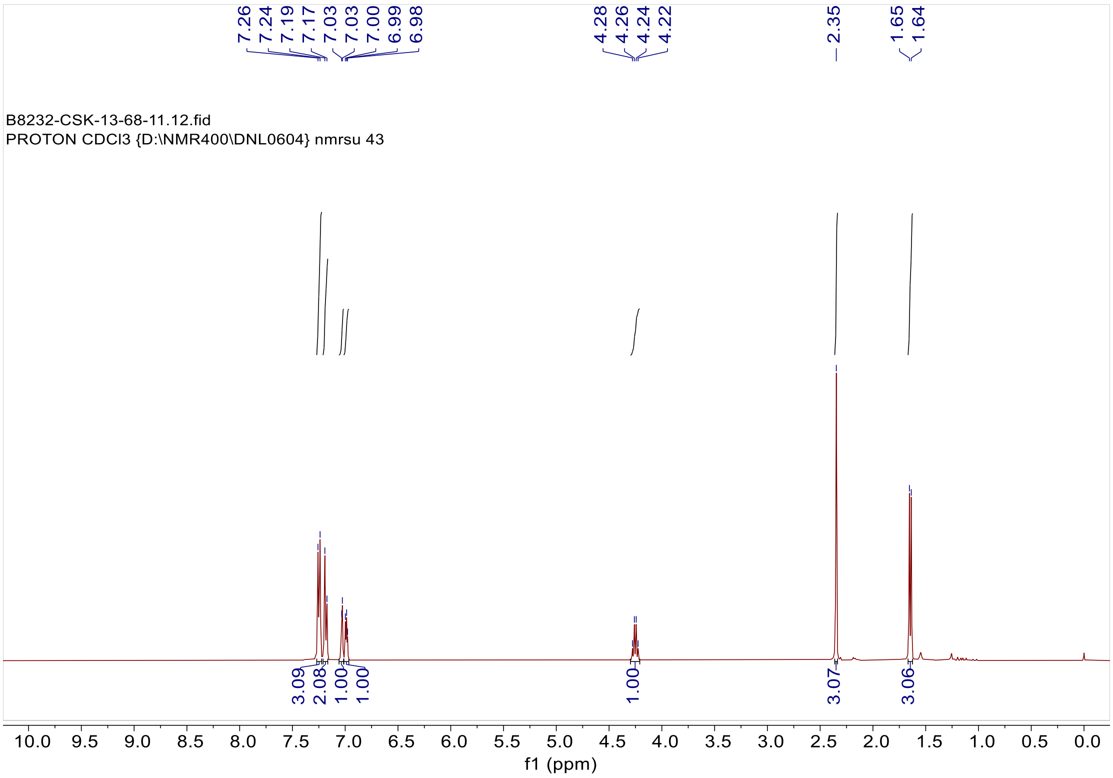

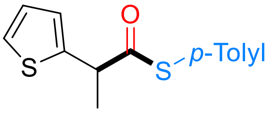

3qa

^{1}H NMR (400 MHz, $CDCl_3$)

^{13}C NMR (100 MHz, $CDCl_3$)

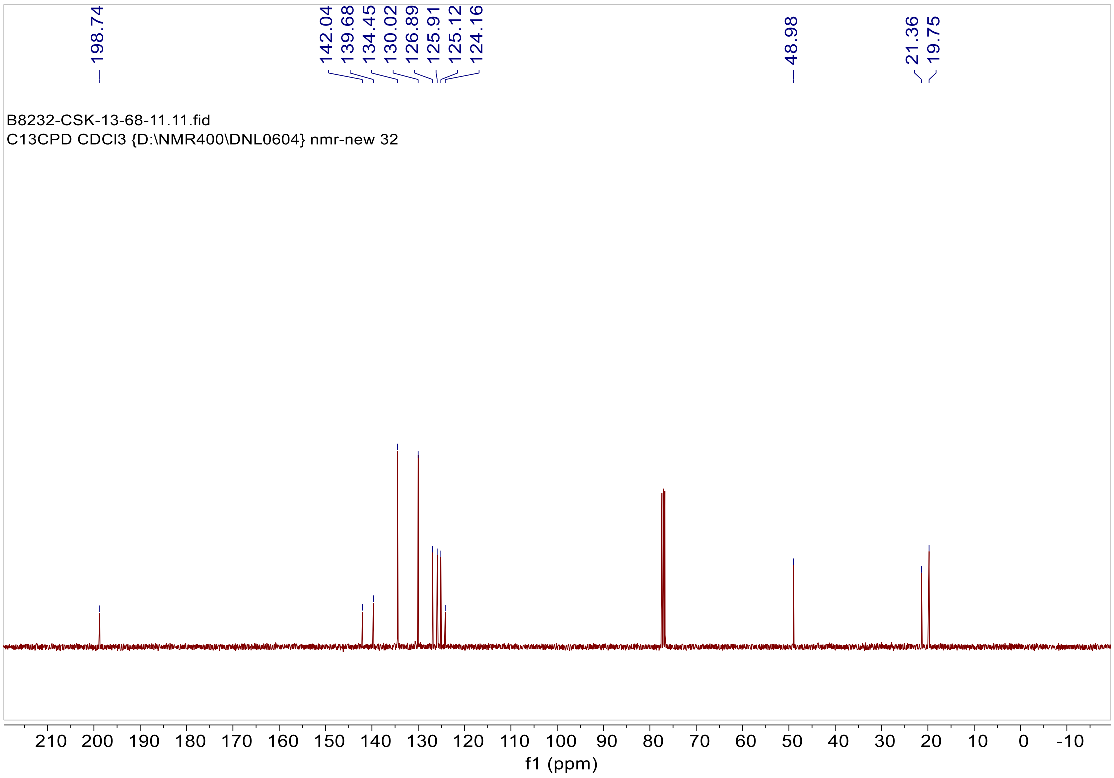

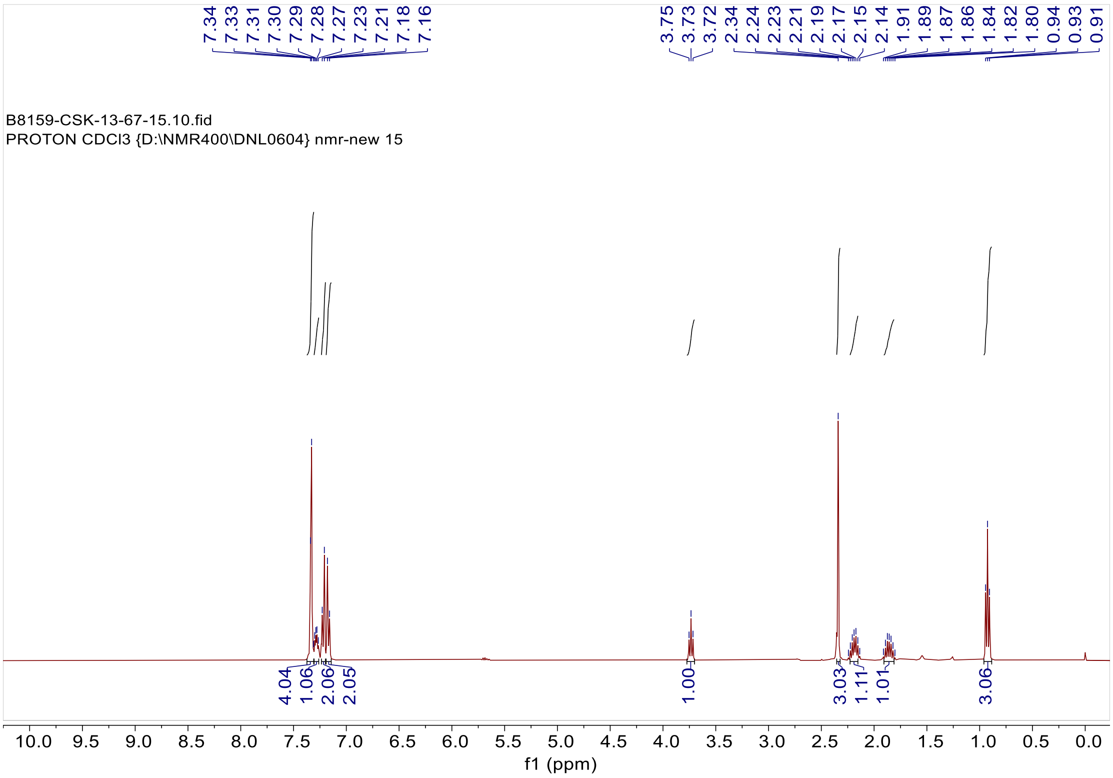

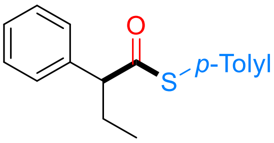

3ra

^{1}H NMR (400 MHz, $CDCl_3$)

^{13}C NMR (100 MHz, $CDCl_3$)

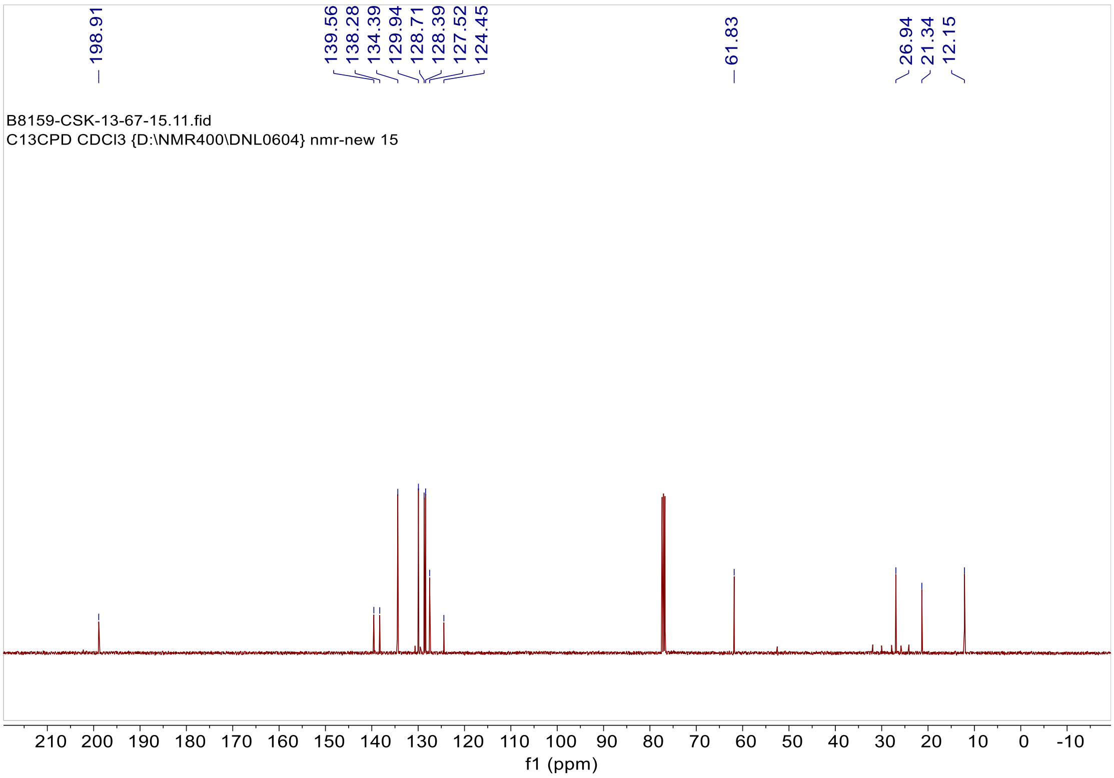

Supplement: Supplementary file 1 [file ol6c02424_si_001.pdf]
